# Immune remodeling via mitochondria-dependent STING activation enhances cabozantinib response in hepatocellular carcinoma

**DOI:** 10.1186/s13046-025-03632-z

**Published:** 2026-01-09

**Authors:** Patricia Rider, Anna Tutusaus, Carlos Cuño-Gómiz, Flavia Savino, Aida Marsal, Neus Llarch, Gemma Iserte, Anna Colell, Pablo García de Frutos, Tania Hernáez-Alsina, Marco Sanduzzi-Zamparelli, Montserrat Marí, María Reig, Albert Morales

**Affiliations:** 1 Department of Molecular and Cellular Biomedicine, Institute of Biomedical Research of Barcelona (IIBB), CSIC, Barcelona, 08036 Spain; 2 Institut d’Investigacions Biomèdiques August Pi i Sunyer (IDIBAPS), Barcelona, Spain; 3 Barcelona Clinic Liver Cancer (BCLC) Group, Liver Oncology Unit, Liver Unit, Hospital Clinic of Barcelona, Barcelona, Spain; 4 Centro de Investigación Biomédica en Red en Enfermedades Hepáticas y Digestivas (CIBEREHD), Madrid, Spain; 5 Departament de Biomedicina, Facultat de Medicina, Universitat de Barcelona, Barcelona, Spain; 6 Centro de Investigación Biomédica en Red en Enfermedades Neurodegenerativas (CIBERNED), ISCIII, Madrid, Spain; 7 Centro de Investigación Biomédica en Red en Enfermedades Cardiovasculares (CIBERCV), Madrid, Spain; 8 Unidad Asociada IMIM/IIBB-CSIC, Barcelona, Spain; 9 Digestive Diseases Department, Hospital Universitario San Pedro, Rioja Salud, Logroño, Spain; 10 Barcelona University, Barcelona, Spain

**Keywords:** Tyrosine kinase inhibitor, cGAS-STING pathway, Mitochondrial DNA, Innate immunity, Tumor microenvironment, HCC biomarkers

## Abstract

**Background:**

Cabozantinib, a tyrosine kinase inhibitor (TKI) approved for advanced hepatocellular carcinoma (HCC), has established clinical benefit although the underlying immunomodulatory mechanisms, particularly those involving mitochondrial stress and the cGAS/STING pathway, remain poorly defined.

**Methods:**

We assessed cabozantinib’s effects on mitochondrial integrity and innate immune signaling in hepatoma cells and macrophage cell lines, analyzing mitochondrial depolarization, reactive oxygen species production, cytosolic release of mitochondrial DNA (mtDNA), activation of the cGAS/STING pathway and induction of type I interferon-stimulated genes (ISGs). Functional relevance was tested by mtDNA depletion and CRISPR-mediated STING knockdown. The in vivo effects of cabozantinib and the STING agonist DMXAA were examined in immunocompetent mouse models. Translational relevance was evaluated by multiplex proteomic profiling of serum samples from 18 cabozantinib-treated HCC patients across two independent cohorts.

**Results:**

Cabozantinib induced mitochondrial depolarization, oxidative stress, and cytosolic mtDNA release, resulting in STING-dependent signaling and ISG upregulation in hepatoma cells. Disruption of mtDNA or STING abrogated these effects. In vivo, cabozantinib reduced tumor growth and promoted tumor-infiltrating lymphocyte activation, which were further enhanced by DMXAA co-treatment. Patient serum proteomics revealed consistent increases in immune and stress-related proteins (e.g., granzyme B, HO-1, CAIX, CXCL13) and decreases in angiogenic and immunosuppressive factors (e.g., VEGFR-2, ANGPT1/2, CCL17), paralleling the systemic immune remodeling observed in preclinical models. Both baseline immune signatures and treatment-induced protein shifts were associated with clinical outcome.

**Conclusions:**

Cabozantinib promotes tumor immunogenicity through mitochondrial disruption and cGAS/STING activation, leading to immune remodeling in HCC. These findings provide mechanistic insight into the immunomodulatory effects of cabozantinib, support rational combinations with STING agonists, and highlight candidate biomarkers for predicting therapeutic response in TKI-treated patients.

**Supplementary Information:**

The online version contains supplementary material available at 10.1186/s13046-025-03632-z.

## Background

Hepatocellular carcinoma (HCC) is the most common form of primary liver cancer and a leading cause of cancer-related mortality worldwide [1, 2]. Immune checkpoint inhibitors (ICIs) have improved outcomes in a subset of patients [3–5], but more than 70% of them are either ineligible or fail to respond. For these patients, tyrosine kinase inhibitors (TKIs) such as sorafenib [6] or cabozantinib [7] remain important therapeutic options [8]. Although no clear consensus exists for optimal second-line treatment, cabozantinib has emerged as a valuable choice, supported by evidence from recent clinical trials, including phase II studies [9]. Initially developed to target VEGFR, MET, and AXL signaling [7], cabozantinib exerts pleiotropic effects on both tumor growth and the tumor microenvironment; however, its immunomodulatory mechanisms remain incompletely defined.

Recent studies have highlighted mitochondria as critical sensors and immunomediators of stress-induced tumor cell death [10, 11]. The role of mitochondria extends beyond cell death induction to include modulation of antitumor immunity. In particular, mitochondrial dysfunction and the associated release of mitochondrial DNA (mtDNA) into the cytosol have been implicated in the activation of the cGAS/STING (cyclic GMP-AMP synthase/Stimulator of Interferon Genes) pathway [12, 13], which is a key sensor of cytosolic DNA that drives type I interferon responses, bridging innate and adaptive immunity.

In cancer therapy, STING signaling has recently emerged as a critical mediator of antitumor immunity [14], particularly in response to genotoxic stress or cytoplasmic DNA accumulation [15, 16]. Since STING activation has been shown to improve treatment efficacy in other tumors [17–20], it is important to investigate whether this pathway also contributes to the activity of cabozantinib in HCC. In this context, the interplay between cabozantinib-induced mitochondrial damage, innate immune activation, and the therapeutic response in HCC could be a feasible strategy to further increase cabozantinib effectiveness.

In this study, we revealed that cabozantinib promotes mitochondrial dysfunction and mtDNA-dependent activation of the STING pathway during HCC therapy. This activation is associated with increased type I interferon responses, immune cell recruitment, and reprogramming of the tumor immune microenvironment. We identified immune and inflammatory signatures indicative of STING pathway engagement, both in preclinical models and in serum samples from cabozantinib-treated HCC patients. Our findings highlight a previously underrecognized mechanism of action for cabozantinib and support the use of combination strategies with STING agonists in HCC therapy.

## Methods

### Reagents

Dulbecco’s Modified Eagle’s Medium (DMEM), Dulbecco’s phosphate-buffered saline (DPBS), trypsin-EDTA, penicillin-streptomycin and dimethyl sulfoxide (DMSO), MTT (3-(4,5-dimethylthiazol-2-yl)-2,5-diphenyl tetrazolium bromide, M2128), Dihydroethidium (DHE, D7008) and Hoechst 33258 (B1155) were purchased from Sigma–Aldrich (St. Louis, MO, USA). Proteinase inhibitors were from Roche (Madrid, Spain). ECL western blotting substrate, 2’,3’-Dideoxycytidine (L10619) and HEPES (15630) was from Thermo Fisher Scientific (Rockford, IL, USA). Novex Sharp Pre-Stained Protein Standard (LC5800) (T-3168) and JC-1 (C5,5′,6,6′-tetrachloro-1,1′,3,3′-tetraethylbenzimidazolyl-carbocyanine iodide, T-3168) was from Invitrogen Life Technologies (Carlsbad, CA, USA). Cabozantinib, Lenvatinib (HY-10981), H-151 (HY-112693), DMXAA (HY10964), SR-717 (HY131454), MSA-2 (HY136927), were from MedChem Express (Monmouth Junction, NJ, USA). Sorafenib (BAY 43-9006, Nexavar) is manufactured by Bayer (Leverkusen, Germany).

### Cell culture and 3D tumor liver spheroid generation

Liver tumor cell lines Hep3B, Hepa1-6 and PLC/PRF/5 (European Collection of Animal Cell Cultures (ECACC) were grown in DMEM culture medium supplemented with 10% fetal bovine serum (FBS, Gibco) at 37 °C and 5% CO2. The human liver cell line BCLC5 were derived from hepatocellular carcinoma tissues [21] and grown in 1:1 solution of DMEM high glucose and Ham’s F-12 Nutrient Mixture (Sigma-Aldrich, Sant Louis, MO, USA) supplemented with 10% FBS, 1% non-essential amino acids, 1 mM sodium pyruvate, 100 U/mL penicillin and 100 µg/mL streptomycin. Mouse Raw264.7 and THP-1 human monocytic cells (Sigma-Aldrich, St. Louis, MO, USA) were cultured in suspension in RPMI supplemented with 10% FBS at 37 °C in a 5% CO2 atmosphere. Macrophages were activated in the presence of 100 ng/mL phorbol 12-myristate 13-acetate (PMA) for two days. Afterwards, the PMA-containing media was removed; cells were washed and left with RPMI + 2.5% FBS before the start of each experiment. Hep3B/Hepa1-6 cell spheroids were generated and plated in BIOFLOAT™ round base 96 well plates (Ref 83.3925.400), allowing spheroids to aggregate for 48 h before treatments. Tumor liver spheroids were kept at 37 °C and 5% CO2 for 7 days and growth was monitored daily.

### Cell viability

Cell viability was determined by MTT assay; 1 × 10^4^ cells/well were seeded in a 96-well plate and incubated at 37 °C and 5% CO2. After overnight treatments, 10 µL of MTT reagent (5 mg/mL) were added and incubated for 2 h. After removal of the medium, formazan crystals from dried plates were dissolved with 100 µL of 1-propanol. Absorbance was measured in a plate reader (Multiskan^®^ Spectrum, Thermo Fisher Scientific, Rockford, IL, USA) at 570 nm and 630 nm and cell viability calculated with untreated cells. LDH activity was measured in medium of treated cells using the CyQUANT™ LDH Cytotoxicity Assay Kit (ThermoFisher Scientific) following manufacturer protocol.

### Cellular fractionation

In order to extract cytosolic mtDNA a protocol that utilizes detergent-based buffers and differential centrifugation was performed. First, 1 × 10^6^ cells were seeded in a 6-well plate. After treatments, cells were scrapped with DPBS and centrifuged at 200 g for 5 min at room temperature. Cell pellet was resuspended in digitonin lysis buffer (50ug/ml digitonin, 50mM HEPES,150mM NaCl) by pipetting gently up and down and incubated for 5–8 min to allow selective plasma membrane permeabilization. Lysate was centrifuged 3 min at 14,000 g at 4 °C and the resulting supernatant which corresponds to the cytosolic fraction was transferred to a new tube. Cell pellet was resuspended with lysis buffer and saved as whole-cell extract fraction (normalization control). Fractions are then immunoblotted to assess purity, and DNA is precipitated and quantified by SYBR Green-based qPCR approach.

### Reactive oxygen species (ROS) measurement

Cellular ROS generation was quantified using dihydroethidium (DHE) probe that mainly targets the superoxide anion; 1 × 10^4^ cells/well were seeded in 96-well plates. After treating cells with indicated drugs, DHE probe was added for 30 min. After probe internalization, 2 washes were performed with DMEM without phenol red and photos of random fields were taken using a Leica-CTR4000 microscope.

### Mitochondrial membrane potential assay

JC-1 is a fluorescent cationic dye used as an indicator of mitochondrial potential in cells. Mitochondrial depolarization is assessed by a decrease in the red (J-aggregates)/green (J-monomers) fluorescence intensity ratio. To determine the mitochondrial membrane potential, 1 × 10^4^ cells/well were seeded in 96-well plates and incubated at 37 °C and 5% CO2. Cells were treated, and after, JC-1 dye was incubated for 15 min. Photos were taken with a Leica-CTR4000 microscope and LAS software.

### Immunoblot analysis

Tumor and cell lysates were prepared in RIPA buffer plus proteinase inhibitors. Samples containing 10 to 30 µg were separated by 10%–15% SDS-PAGE. Proteins were transferred to nitrocellulose membranes, blocked in 5% nonfat milk or 5% BSA for 1 h at room temperature, and incubated overnight at 4 °C with the primary antibodies: STING Rabbit 1/1000 (mAb 50494), phospho-STING Rabbit 1/1000 (mAb 72971), cGAS Rabbit 1/1000 (mAb 31659), TBK/NAK Rabbit 1/1000 (mAb3504), Phospho-TBK1/NAK Rabbit 1/1000 (mAb 54839), IRF3 Rabbit 1/1000 (mAb 4302); Phospho-IRF3 Rabbit 1/1000 (mAb 29047) from Cell Signaling Technology. NFkB p65 Mouse 1:250 (sc8008), and TOM20 Rabbit 1/500 was purchased from Santa Cruz Biotechnology. Membranes were then washed and incubated with appropriate HRP-conjugated secondary antibodies (anti-rabbit IgG 1:3000 (#7074, Cell Signaling), anti-mouse IgG2a (sc-2061, Santa Cruz Biotechnology) or β-Actin 1:40000 (#A3854, Sigma-Aldrich) and imaged using the ChemiDoc Touch Imaging.

### RNA isolation and real time RT-PCR

Total RNA was isolated with TRIzol reagent and 1 µg of RNA was reverse transcribed with iScript™ cDNA Synthesis Kit (Biorad, Berkeley, CA, USA). Real-time PCR was performed with iTaq™ Universal SYBR^®^ Green Supermix (Biorad) following the manufacturer’s instructions. The primers sequences used are listed in Table 1.

**Table 1:** Primers used with their sequences

Primer	Fw 5’-3’	Rv 5’-3’
*h- 18 S*	CCGAAGATATGCTCATGTGG	TCTTGTACTGGCGTGGATTC
*h- IFNB*	CTTGGATTCCTACAAAGAAGCAGC	TCCTCCTTCTGGAACTGCTGCA
*h- RSAD2*	CCAGTGCAACTACAAATGCGGC	TCGGTCTTGAAGAAATGGCTCTCC
*h- CXCL10*	GGTGAGAAGAGATGTCTGAATCC	GTCCATCCTTGGAAGCACTGCA
*h- MX1*	GGCTGTTTACCAGACTCCGACA	CACAAAGCCTGGCAGCTCTCTA
*h-STING*	CCTGAGTCTCAGAACTGCC	GGTCTTCAAGCTGCCCACAGTA
*h-ACTIN*	AGAAAATCTGGCACCACACC	AGAGGCGTACAGGGATAGCA
*h- Mt- D-loop*	CTAAATAGCCCACACGTTCCC	AGAGCTCCCGTGAGTGGTTA
*h-B2M*	GCTGGGTAGCTCTAAACAATGTATTCA	CCATGTACTAACAAATGTCTAAAATGGT
*m-Actin*	GACGGCCAGGTCATCACTAT	CGGATGTCAACGTCACACTT
*m-18s*	CGCGGTTCTATTTTGTTGGT	AGTCGGCATCGTTTATGG TC
*m-Oas1*	TGCGAGGTTCAGCATGAGAG	CCGAAGCCCCATAAAGCAGA
*m-Cxcl10*	GGAGTGAAGCCACGCACAC	ATGGAGAGAGGCTCTCTGCTGT
*m-Cxcl9*	CCTAGTGATAAGGAATGCACGATG	CTAGGCAGGTTTGATCTCCGTTC
*m- Ifnb*	CTCCAGCTCCAAGAAAGGAC	TGGCAAAGGCAGTGTAACTC
*m-Ifi44*	CTGATTACAAAAGAAGACATGACAGAC	AGGCAAAACCAAAGACTCCA
*m-Zbp1*	TCAAAGGGTGAAGTCATGGA	GTGGAGTGGCTTCAGAGCTT
*m-Sting*	GGAACACCGGTCTAGGAAGC	CAAGTGTCCGGCAGAAGAGT
*m- Nkp46/Ncr1*	TAGGGCTCACAGAGGGACATAC	GTAGGTGCAAGGCTGCTGTTCT
*m-Nkg2d-Klrk1*	CCTATCACTGGATGGGACTGGT	GCTTGAGCCATAGACAGCACAG
*m-Tnf*	CTGAACTTCGGGGTGATCGGT	ACGTGGGCTACAGGCTTGTCA
*m-Il 15*	GCTGAGTTCCACATCTAACAGC	AGCAAGGACCATGAAGAGGC
*m-Fasl*	GAAGGAACTGGCAGAACTCCGT	GCCACACTCCTCGGCTCTTTTT
*m-Gzmb*	CAGGAGAAGACCCAGCAAGTCA	CTCACAGCTCTAGTCCTCTTGG
*m-Cxcr3*	TACGATCAGCGCCTCAATGCCA	AGCAGGAAACCAGCCACTAGCT
*m-Pd1*	CGGTTTCAAGGCATGGTCATTGG	TCAGAGTGTCGTCCTTGCTTCC
*m-Pdl1*	TGCGGACTACAAGCGAATCACG	CTCAGCTTCTGGATAACCCTCG
*m-mtDloop*	AATCTACCATCCTCCGTGAAACC	TCAGTTTAGCTACCCCCAAGTTTAA
*m-tert*	CTAGCTCATGTGTCAAGACCCTCTT	GCCAGCACGTTTCTCTCGTT

### Immunofluorescence

Hep3B cells were seeded onto glass coverslips and stimulated with cabozantinib for the indicated times. Cells were then fixed in 4% PFA for 15 min, permeabilized with 0,2% Triton X-100 for 10 min, blocked in a BSA 3% solution for 30 min and incubated overnight at 4 °C in a dark chamber with primary antibodies diluted in blocking buffer as follows: mouse anti-DNA (Progen, 61014, 1:250) and rabbit anti-TOM20 (Santa Cruz, sc-11415, 1:500). Following two PBS washes, one-hour incubation at room temperature with secondary antibodies (goat antimouse 488 Santa Cruz Biotechnology (1:200), and mouse anti-rabbit 594 Santa Cruz Biotechnology (1:200)) was performed. After 15 min Hoechst 1/1000 incubation, coverslips were mounted onto the microscope slides using Fluoromount-G (Invitrogen). Pictures, ten random fields per sample, were taken at the Andor Dragonfly Confocal Microscope with the 60× oil objective.

### Mitochondrial DNA-depleted cells

Depletion of mtDNA was performed in DMEM medium + 10% FBS containing 150 µM 2’,3′-Dideoxcytidine (ddC), a chain terminating nucleoside analog that inhibits mtDNA replication. After 48 h incubation, the depletion was analyzed using real time qPCR to measure expression of mitochondrial genes or nuclear genes. The primer sequences are provided in Table 1.

### Generation of CRISPR-Cas9 STING Knockout cell lines

Murine (Hepa1-6) and human (Hep3B) hepatoma cell lines were transduced with custom CRISPR-Cas9 lentiviral particles (Sigma-Aldrich) carrying guide RNA (gRNA) targeting the *Tmem173* or *TMEM173* gene, along with a puromycin resistance cassette. Transductions were performed according to the manufacturer’s Sigma-Aldrich Lentiviral Transduction protocol using polybrene (8 µg/mL), followed by puromycin selection (2 µg/mL for murine, 1 µg/mL for human cells) for 5–7 days. STING knockout was confirmed by Western blot and RT-qPCR, showing complete or strongly reduced expression at both protein and mRNA levels. The CRISPR constructs were pre-designed and validated by the supplier.

### Immunohistochemical staining

Tumors were fixed with 10% paraformaldehyde for 48 h, paraffin-embedded, and cut in 5-µm sections. Heat-induced antigen retrieval was performed in citrate buffer, and after blocking endogenous peroxidase, slides were incubated with the following antibodies: anti-Granzyme B (E5V2L) (1:100, Rabbit mAb #44153, Cell Signaling Technology), anti-CD8α (D4W2Z) (1:100, Rabbit mAb #98941, Cell Signaling Technology) and anti-FoxP3 (D6O8R) (1:100, Rabbit mAb, #12653, Cell Signaling Technology). Then, slides were incubated with a biotinylated antibody and developed with the ABC-HRP Kit (Vector Laboratories, Burlingame, CA, USA) and peroxidase substrate DAB (Sigma-Aldrich, St. Louis, MO, USA). Afterward, slices were examined with a Zeiss Axioplan microscope equipped with a Nikon DXM1200F digital camera. The CD8α, Granzyme B and FoxP3 cell count was quantified in five randomly selected fields from each animal and analyzed using ImageJ 1.54e software.

### Plasma sample preparation and quantitative analysis of mtDNA content

To measure mtDNA and nuclear DNA directly in plasma of mice, without nucleic acid purification, samples were solubilized with a proportion of 1:1 (volume/volume) of DNA/RNA/protein solubilization reagent 100ST (#DCQ100ST, DirectQuant, Lleida, Spain), incubated for 3 min at 90 °C with agitation at 750 rpm to complete the solubilization. The resulting sample mixture was used to measure the absolute copy number of nucleic acids by real-time RT-PCR. mtDNA content was determined by quantification of m-D-Loop by relative cycle threshold (ΔΔCt). Results were corrected for differences in starting DNA concentration by measurement of the single-copy nuclear genes m-TERT.

### Animal procedures

All animal procedures were performed according to protocols approved by the Animal Experimentation Ethics Committee from the University of Barcelona. For initial tumor inoculation, in vitro expanded Hepa1-6 cells (2.5 × 10^6^ suspended in DMEM/Matrigel) were subcutaneously injected into the right and left flank of 5-week-old male C57BL/6 mice. After 10 to 20 days of subcutaneous growth and mouse euthanasia, tumors were collected and cut into small fragments (70 or 100 mg) which were then subcutaneously engrafted on recipient mice. When tumors reached a volume of 200–300 $\:{\mathrm{m}\mathrm{m}}^{3}$, animals were randomly assigned to receive: (a) Placebo: saline solution; (b) Cabozantinib: 10 mg/kg via oral gavage 3 times per week; (c) DMXAA i.p. at 10 mg/kg once a week for a total of 4 doses (d) Combination of cabozantinib with DMXAA. Tumor measurements were taken every 2–3 days using a vernier caliper, and volumes were calculated based on the formula length×width^2^ × 0.5. In addition, tumors collected at the time of sacrifice were fixed in 10% paraformaldehyde in order to carry out immunohistochemistry and frozen for RNA/protein analysis.

For isolation of mouse Peritoneal Macrophages (PMs), male C57BL6/J mice aged 8–12 weeks were treated with 3% thioglycolate broth (Sigma-Aldrich) 4 days before sacrifice. PMs were isolated by lavage using ice-cold Ca2 + and Mg2+-free PBS and plated in 12-well plates at a density of 10^6^ cells per well in complete RPMI medium.

### Isolation of tumor infiltrating leukocytes

Subcutaneous tumors were harvested and stored overnight in MACS Tissue Storage solution (Miltenyi Biotec) at 4 °C. Tumors were then washed twice in PBS and digested with 0.04 mg/mL Liberase TM and 0.1 mg/mL DNAse I (Roche) in RPMI 1640 medium (Sigma-Aldrich) supplemented with 10% FBS for 30 min at 37 °C. Samples were then incubated for 10 more minutes on ice and filtered through 70 μm cell strainers. Leukocytes were then purified by lysing contaminating red blood cells with ACK buffer (150 mM NH4Cl, 10 mM KHCO3, 0.01 mM EDTA; pH 7.2) for 5 min at room temperature; and by density gradient centrifugation in 40 and 80% Percoll (Cytiva).

### Leukocyte staining for flow cytometry analysis

Isolated leukocytes were transferred to FACS tubes and stained with LIVE/DEAD Blue Viability staining kit (Invitrogen). Cells were then blocked with an α-CD16/CD32 antibody (BioLegend) and fresh stained with 1:100 α-CD69 antibody (Supplementary Table 1). Following fresh staining, cells were fixed and permeabilized with Foxp3 Transcription Factor Staining Buffer Set (Invitrogen), blocked with 2% mouse serum, and then stained overnight for the rest of surface and intracellular markers (Supplementary Table 1), as recommended by manufacturer and other authors [22]. Stained cells were analyzed in a 5-laser Cytek Aurora spectral flow cytometer (Cytek Biosciences).

Spectral unmixing was performed using the AutoSpectral 0.8.4 R package, developed by Burton and colleagues (10.1101/2025.10.27.684855), in R 4.5.1. Unmixed .fcs files were analyzed using Flowjo v10.10 (BD Biosciences).

### Assessment of Immunogenic cell death

Extracellular ATP was measured by bioluminescent assays (RealTime-Glo™ Extracellular ATP Assay, Promega), following manufacturer instructions. Briefly, 6000 cells were plate in black plates (Brand), treated with cabozantinib with ATP Assay Reagent and luminescence was collected at different time points for 24 h. HMGB1 secretion in the supernatant of cells was measured by western blot analysis with the primary rabbit antibody HMGB1 [EPR3507] (1:10000, ab79823, Abcam). Media from cabozantinib treated cells was first concentrated with Pierce™ Protein Concentrators, PES, 3 K (Thermo Scientific).

### Olink analysis

Proteomic profile of patients’ serum before and after 4 weeks of cabozantinib treatment was performed using Olink^®^ Immuno-Oncology panel (Olink Proteomics AB). Analysis was conducted at the Immune Response and Biomarker Core Facility at ISGlobal, an Olink^®^ Certified Service Provider. Data was normalized and expressed as NPX, Olink’s relative protein quantification unit on a log2 scale. Analyses were performed in R 4.5.0 using Olink^®^ Analyze. Fold change calculations were performed relative to baseline measurements and compared with paired T-test.

### Human samples

Serum samples were obtained from 18 patients diagnosed with HCC who were treated at the Hepatic Oncology Unit (BCLC), Liver Unit of the Hospital Clinic of Barcelona (Table 2). The study was reviewed and approved by the Hospital Clinic of Barcelona Board of Clinical and Experimental Research (HCB/2017/1016 and HCB/2022/0652) and complied with the Good Clinical Practice guidelines. Patients provided informed consent according to the principles of the Declaration of Helsinki.

**Table 2:** Characteristics of the patients with HCC included in the study

Patient’s Characteristics	All (*n* = 18)	Cohort 1 (*n* = 7)	Cohort 2 (*n* = 11)
Age (years), mean ± SD	71.2 ± 8.1	70.4 ± 6.8	71.6 ± 9.1
Gender (Male), n (%)	12 (66.67%)	6 (85.7%)	6 (54.6%)
Etiology, *n* (%)			
HCV	7 (38.9%)	3 (42.9%)	4 (36.4%)
HBV	1 (5.6%)	0 (0%)	1 (9.1%)
Alcohol	6 (33.3%)	3 (42.9%)	3 (27.3%)
MASH	1 (5.6%)	0 (0.0%)	1 (9.1%)
PBC	1 (5.6%)	0 (0.0%)	1 (9.1%)
No	2 (11.1%)	1 (14.3%)	1 (9.1%)
Overall survival, median [IQR]	10.17 [7.9–14.3]	10.73 [7.2–15.3]	10.07 [8-11.5]
Previous treatment, *n* (%)			
Sorafenib	16 (38.9%)	5 (71.4%)	11 (100%)
Sorafenib/Regorafenib	2 (5.6%)	2 (28.6%)	0 (0%)
Inter-treatment interval, median weeks [IQR]	4.07 [3.1–12.5]	2.43 [2.0-8.4]	7 [3.9–15.0]

### Statistical analysis

Statistical analyses were performed using the GraphPad Prism software version 4.0.2. The results are representative of at least three independent experiments, and data are expressed as the mean ± SEM. Comparisons between two groups were analyzed by unpaired t test for data following a Gaussian distribution or Mann-Whitney test for nonparametric data. For comparisons involving more than two groups one-way ANOVA with a Newman-Keuls post hoc test was used to detect significant differences.

## Results

### Cabozantinib induces mitochondrial-dependent STING activation

Our previous findings have demonstrated that TKIs such as sorafenib and regorafenib induce mitochondrial stress [23, 24]. However, it remains unclear whether cabozantinib exerts similar effects on mitochondrial function, particularly in pathways related to inflammation and immune signaling. To address this, we examined whether cabozantinib activates the cytosolic DNA-sensing pathway, specifically the cGAS/STING axis, which links mitochondrial dysfunction to innate immune activation.

**Fig. 1:**
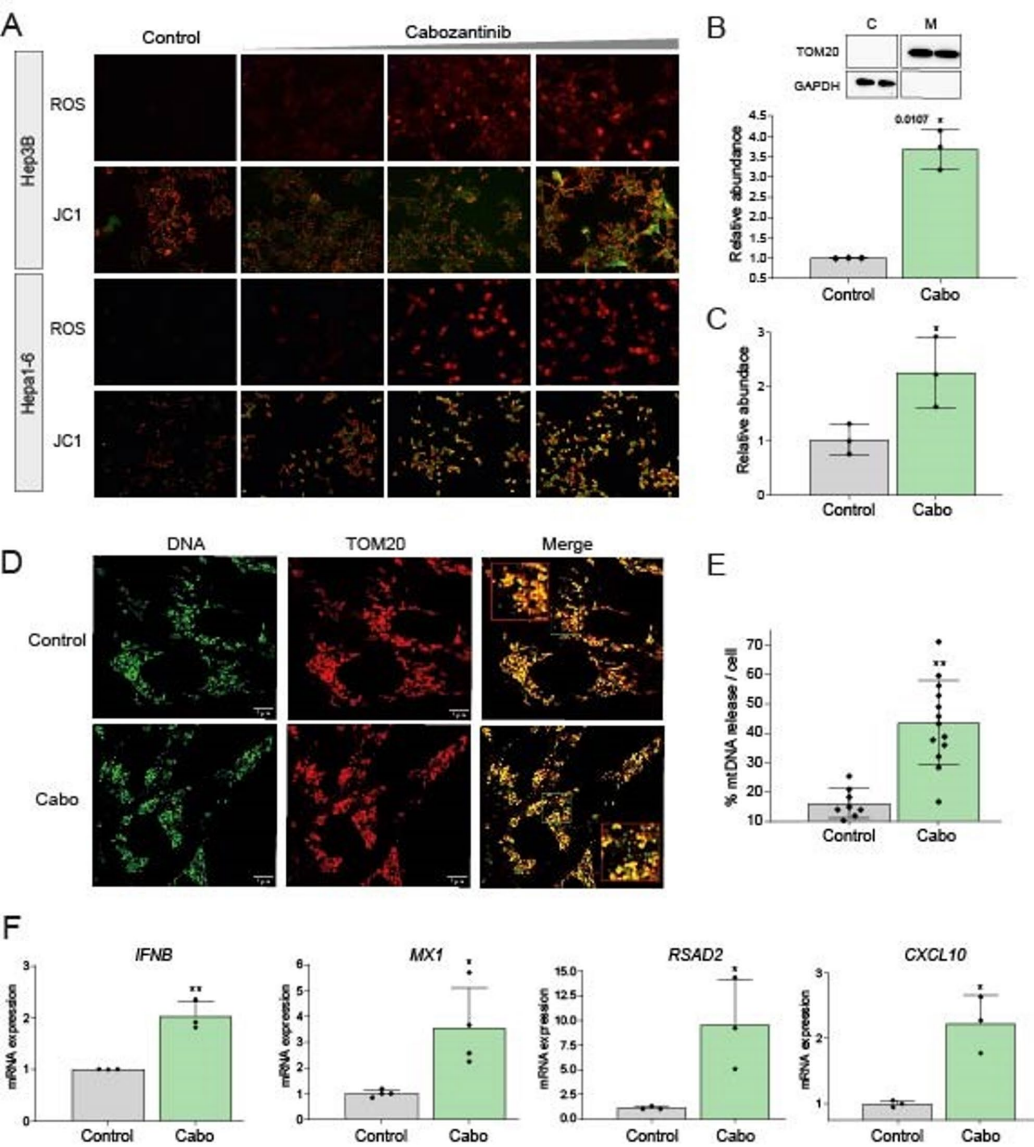
Cabozantinib disrupts mitochondrial integrity, leading to activation of the cGAS/STING pathway. **A** Hep3B cells treated with cabozantinib (5-25-50µM, 8 h) exhibit increased mitochondrial ROS by dihydroethidium (DHE) staining, and loss of mitochondrial membrane potential, shown by JC-1 dye shift from red (polarized) to green (depolarized) mitochondria. **B** Cytosolic mtDNA accumulation after 24 h treatment measured by RT-qPCR after selective digitonin-based cell fractionation (*n* = 3). **C** Quantification of mtDNA in culture supernatants reveals extracellular mtDNA release after cabozantinib treatment (10µM, 24 h) (*n* = 3). **D** Immunofluorescence microscopy shows extranuclear DNA (green) outside TOM20⁺ mitochondria (red), indicating mtDNA leakage into the cytosol. **E** Quantification of extranuclear DNA localization using ImageJ confirms increased mtDNA release. **F** RT-qPCR analysis of type I IFN responses reveals upregulation of *IFNB1* and ISGs (*RSAD2*, *CXCL10*, and *MX1*) following 24 h cabozantinib exposure (*n* = 3). Student t test with *p < 0.05, **p < 0.01

Consistent with prior observations that TKIs disrupt mitochondrial homeostasis and promote mitochondrial membrane permeabilization [25], we observed that cabozantinib treatment significantly increased mitochondrial reactive oxygen species (ROS) levels in hepatoma cells, as detected by DHE staining. This was accompanied by a loss of mitochondrial membrane potential, as indicated by a shift in JC-1 staining from red (polarized mitochondria) to green (depolarized mitochondria) (Fig. 1A).

To determine whether mitochondrial damage leads to the release of mitochondrial damage-associated molecular patterns (DAMPs), we assessed cytosolic mtDNA accumulation in Hep3B cells. Following selective cell fractionation using digitonin lysis buffer and validation by immunoblotting (Fig. 1B), cytosolic DNA was extracted and quantified using RT-qPCR. We observed an increase in mtDNA in the cytosol of cabozantinib-treated cells compared to that in untreated controls (Fig. 1B). Notably, mtDNA was detected in the culture supernatant after 16 h of treatment (Fig. 1C). These results were further supported by immunofluorescence microscopy, which revealed increased extranuclear DNA localized outside the TOM20-positive mitochondria (Fig. 1D), quantified using ImageJ (Fig. 1E). Given that cytosolic mtDNA is a known activator of type I interferon (IFN) responses, we assessed downstream immune activation. Cabozantinib treatment induced a marked increase in *IFNB1* and multiple interferon-stimulated genes (ISGs), including *RSAD2*, *CXCL10*, and *MX1* (Fig. 1F).

**Fig. 2:**
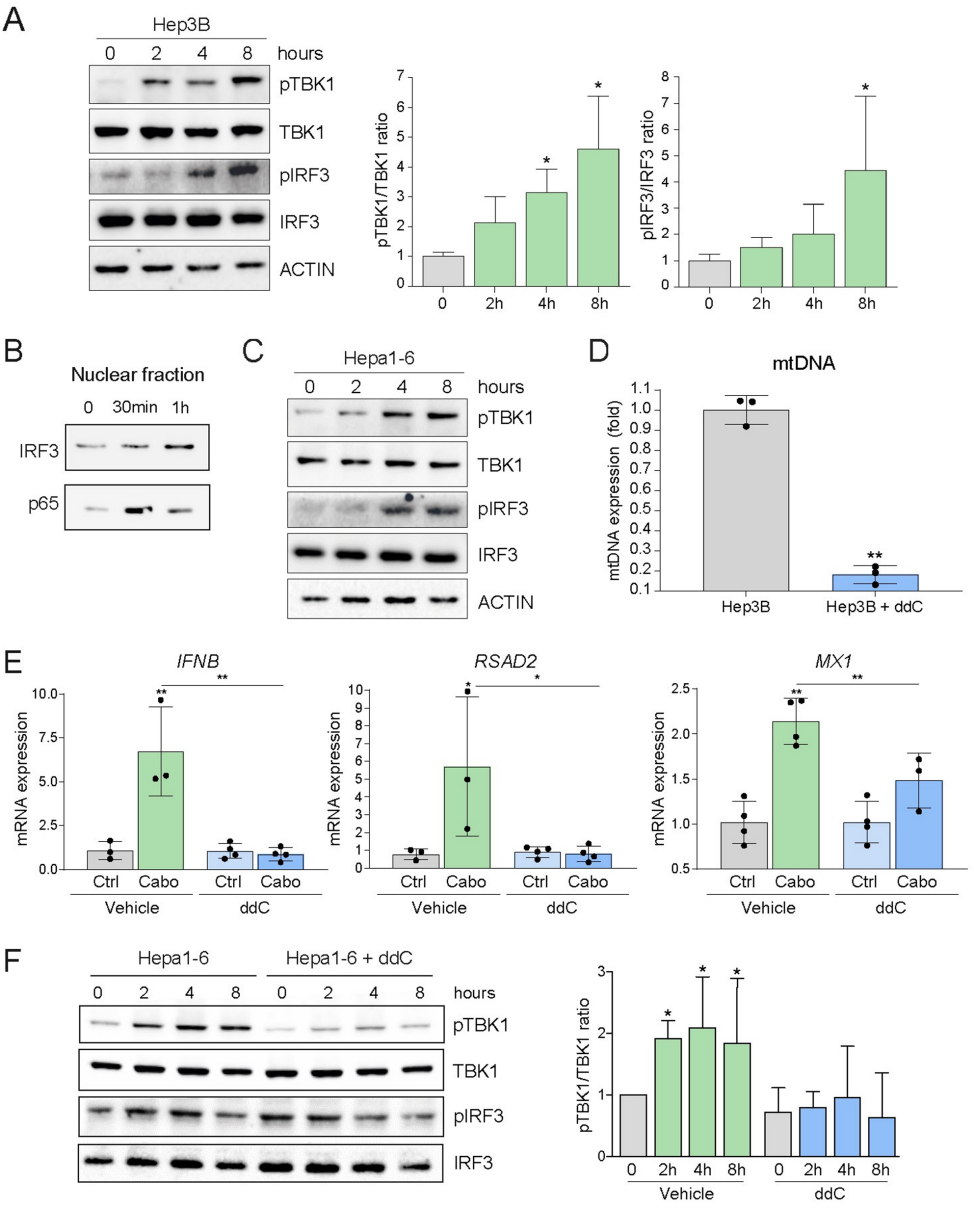
Cabozantinib activates the cGAS/STING signaling cascade in hepatoma cells via mtDNA-dependent mechanisms. **A** Immunoblot analysis of Hep3B cells treated with cabozantinib (50µM) shows time-dependent phosphorylation of TBK1 and IRF3 (*n* = 3). **B** Nuclear translocation of IRF3 and NF-κB p65 after cabozantinib treatment (*n* = 3). **C** STING pathway activation in murine Hepa1-6 cells treated with cabozantinib (50µM) (*n* = 3). **D** Pre-treatment with 2’,3′-dideoxycytidine (ddC, 150µM) for 48 h depletes ~ 80% of mtDNA in Hep3B cells, as quantified by RT-qPCR (*n* = 3). **E** ddC-mediated mtDNA depletion abolishes ISG expression induced by cabozantinib (10µM) as observed for *INFB*, *RASD2*, and *MX1* genes at 24 h (*n* = 3). **F** In Hepa1-6 cells, mtDNA depletion suppresses TBK1 phosphorylation induced by cabozantinib (50µM) (*n* = 3). Student t test or one-way ANOVA with a Newman-Keuls post hoc test with *p < 0.05, **p < 0.01

These findings prompted us to directly assess activation of the cGAS/STING pathway. In cabozantinib-treated Hep3B cells, time-dependent phosphorylation of the STING downstream effectors TBK1 and IRF3 was observed (Fig. 2A). Nuclear translocation of both IRF3 and NF-κB p65 confirmed the dual activation of type I IFN and NF-κB signaling pathways (Fig. 2B). Similar results were observed in murine Hepa1-6 cells (Fig. 2C) and in BCLC5 cells, an HCC patient-derived cell line (Supplementary Fig. 1A), indicating that cabozantinib-induced cGAS/STING activation was conserved across species.

To determine whether these effects depended on mitochondrial DNA, Hep3B cells were pretreated with 2’,3′-dideoxycytidine (ddC), a nucleoside analog that selectively depletes mtDNA. After 48 h, mtDNA levels were reduced by ~ 80% (Fig. 2D). In mtDNA-depleted cells, cabozantinib-induced ISG expression was completely abrogated (Fig. 2E). Moreover, TBK1 phosphorylation was suppressed in Hepa1-6 (Fig. 2F), in PLC/PRF/5 cells (Supplementary Fig. 1B), and in BCLC5 cells (Supplementary Fig. 1A), confirming that mtDNA release is a necessary upstream event for STING activation in response to cabozantinib in HCC cells.

### STING signaling enhances the immunogenic and cytotoxic effects of cabozantinib in HCC models

Given that our earlier findings identified STING activation as a key mediator of cabozantinib-induced signaling, we assessed whether pharmacological activation of STING could enhance cabozantinib’s antitumor efficacy. To this end, murine Hepa1-6 cells were treated with the STING agonist DMXAA (vadimezan), either alone or in combination with cabozantinib.

**Fig. 3:**
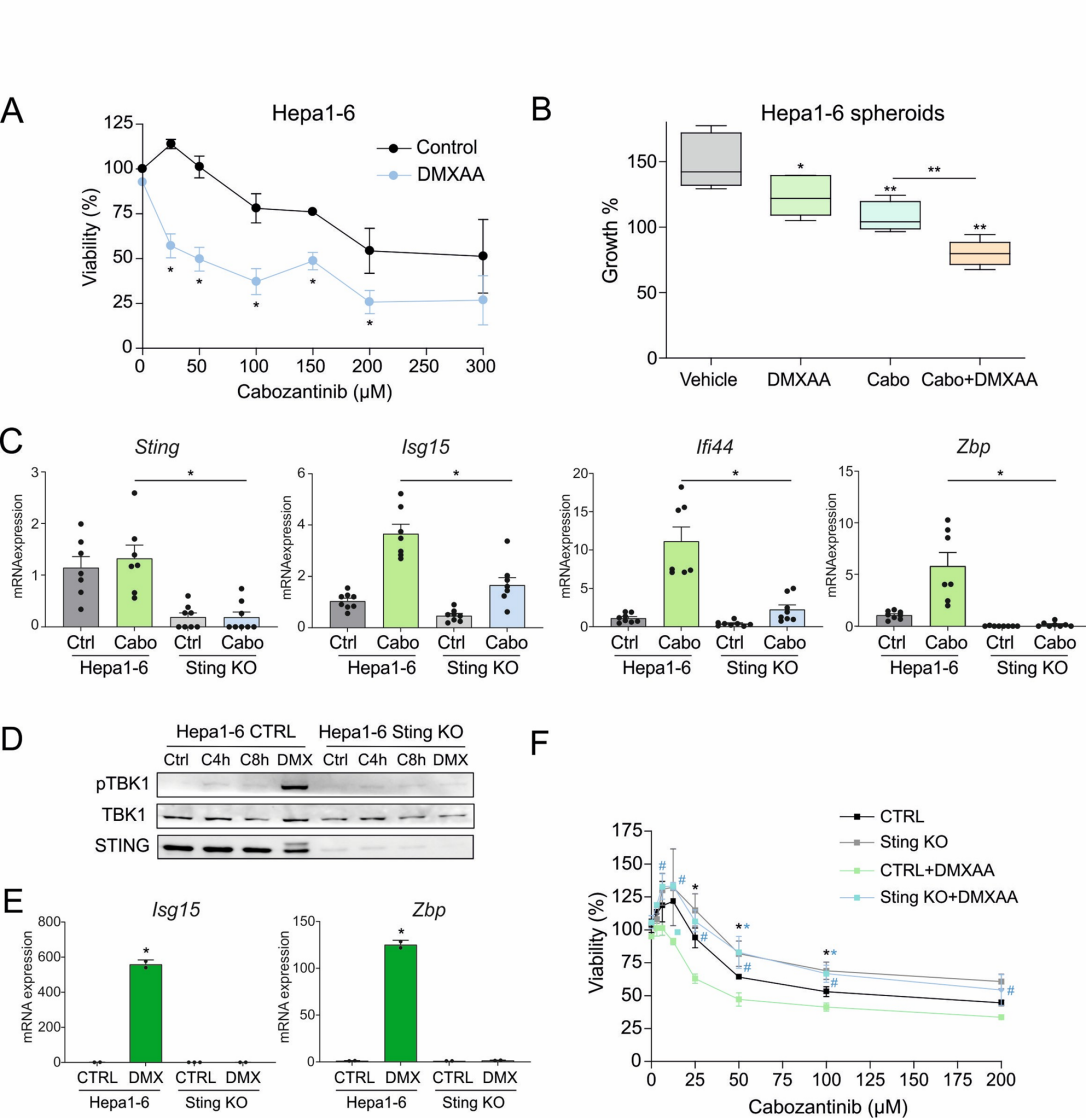
STING signaling enhances cabozantinib-induced cytotoxicity and immunogenicity in HCC models. **A** Viability assessed by MTT assay after combination treatment of murine Hepa1-6 cells with cabozantinib and the murine STING agonist DMXAA (100µM) in 16 h experiments (*n* = 3). **B** Tumor growth in 3D spheroid Hepa1-6 cultures treated with DMXAA and cabozantinib (10µM) for 72 h (*n* = 6). **C** STING knockout (Sting-KO) Hepa1-6 cells generated via CRISPR-Cas9 fail to upregulate interferon-stimulated genes (ISGs) in response to cabozantinib, unlike control cells (*n* = 4). **D** Immunoblotting shows that DMXAA induces TBK1 phosphorylation in control cells but not in Sting-KO cells (*n* = 3). **E** DMXAA-induced ISGs expression is blocked in Sting-KO cells. **F** MTT assays at 16 h in Sting-KO and Hepa1-6 control cells after DMXAA (100µM) and cabozantinib treatments (*n* = 3). One-way ANOVA with a Newman-Keuls post hoc test with *p < 0.05, **p < 0.01

Interestingly, DMXAA, which on its own has no significant effect on Hepa1-6 cells, potentiated cabozantinib-induced cytotoxicity as early as overnight treatment in 2D cultures (Fig. 3A). Similarly, in 3D spheroid cultures, cabozantinib and DMXAA combination significantly reduced tumor spheroid growth, indicating a synergistic interaction between TKI therapy and STING activation (Fig. 3B).

To further dissect the role of STING, Sting-knockout (KO) Hepa1-6 cells were generated using CRISPR-Cas9. In these cells, cabozantinib failed to induce the expression of ISGs, in contrast to the robust ISG upregulation observed in the control cells (Fig. 3C). Likewise, DMXAA treatment led to strong TBK1 phosphorylation and ISG expression in control cells but had no effect in Sting-KO cells (Fig. 3D-E), confirming the specificity and requirement of STING for this response. More importantly, Sting-KO cells displayed increased resistance to cabozantinib-induced cell death, highlighting the critical contribution of STING signaling to drug sensitivity (Fig. 3F). Moreover, while DMXAA enhanced cabozantinib-induced cytotoxicity in wild-type cells, this synergistic effect was completely abolished in the absence of STING (Fig. 3F). Similarly, CRISPR-Cas9 editing diminished STING expression in Hep3B cells (100.0 ± 12.3 vs. 27.7 ± 8.9 in STING KO cells), reduction that was sufficient to confer significant protection against cabozantinib-induced cytotoxicity (Supplemental Fig. 2, upper panel), despite being less pronounced than the strong depletion (100.0 ± 10.6 vs. 7.2 ± 4.6) achieved in Hepa1-6 cells. Confirming STING relevance in this signaling, cabozantinib-induced TBK phosphorylation was reduced in Hep3B STING KO. Moreover, the growth reduction of Hep3B spheroids after cabozantinib exposure for 72 h was partially blocked in STING KO spheroids (Supplemental Fig. 2, lower panel), further supporting the relevance of this pathway in the cytotoxic effect of cabozantinib in human hepatoma cells.

**Fig. 4:**
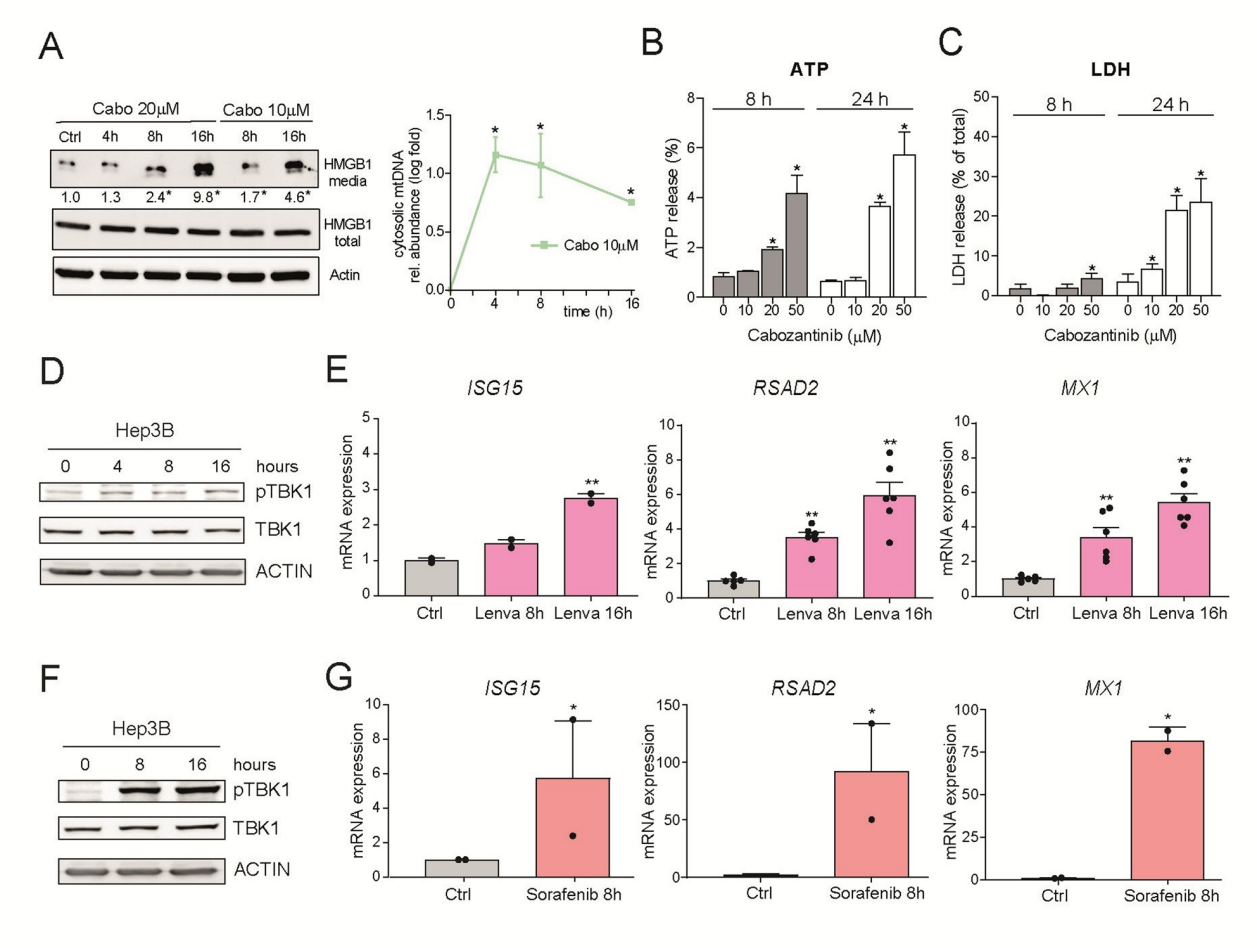
Cabozantinib induces Immunogenic cell death, while lenvatinib and sorafenib are also inducers of STING signaling and ISGs. **A**-**C** HMGB1, mtDNA, ATP and LDH are released by Hep3B cells at different time-points and concentrations of cabozantinib. **D** and **F** Lenvatinib (20µM) and sorafenib (20µM) induce TBK phosphorylation in Hep3B cells. **E** and **G** Lenvatinib (5µM) and sorafenib (10µM) increase the transcriptional expression of ISGs in Hep3B cells. (*n* = 3–6) *p < 0.05

To test the hypothesis that cabozantinib causes immunogenic cell death (ICD) in HCC cells, as recently reported in prostate cancer cells [26], we searched for the cytosolic release of ICD-associated molecules such as HMGB1 or ATP. Of note, we detected in the medium of cabozantinib-treated Hep3B cells the significant increase of HMGB1 and mtDNA (Fig. 4A) and ATP (Fig. 4B) after 8 h at 20 µM concentration, while LDH release (Fig. 4C), indicative of cellular death with membrane rupture, was not yet detected. Therefore, the release of these immunogenic molecules, following the early activation of the cGAS/STING pathway (Fig. 2), was not a consequence of the cell lysis but was instead likely associated with an immunogenic mechanism, as previously suggested, involving STING. Of note, at these concentrations of cabozantinib, no relevant increase in caspase-3 activation was detected, and pan-caspase inhibition did not affect cabozantinib-induced cell death in MTT assays (data not shown).

To further demonstrate the importance of this pathway in HCC treatment, we tested the effect of other TKIs such as lenvatinib or sorafenib, previously used as first-line therapy against advanced HCC. Lenvatinib also activated the cGAS/STING signaling as denoted by TBK phosphorylation (Fig. 4D) and enhanced ISG expression (Fig. 4E). Moreover, STING agonism potentiated lenvatinib cytotoxicity in Hepa1-6 and Hep3B cells (Supplemental Fig. 3A-B). In addition, sorafenib exposure induced a strong TBK phosphorylation in Hep3B cells (Fig. 4F) with concomitant ISGs upregulation (Fig. 4G). Together, these results demonstrate that STING is essential for both the transcriptional activation and efficacy of cabozantinib and suggest STING activators as rational strategy to boost TKI-induced immunogenic stress in HCC.

### Cabozantinib activates STING signaling in macrophages and is potentiated by pharmacologic agonists

**Fig. 5:**
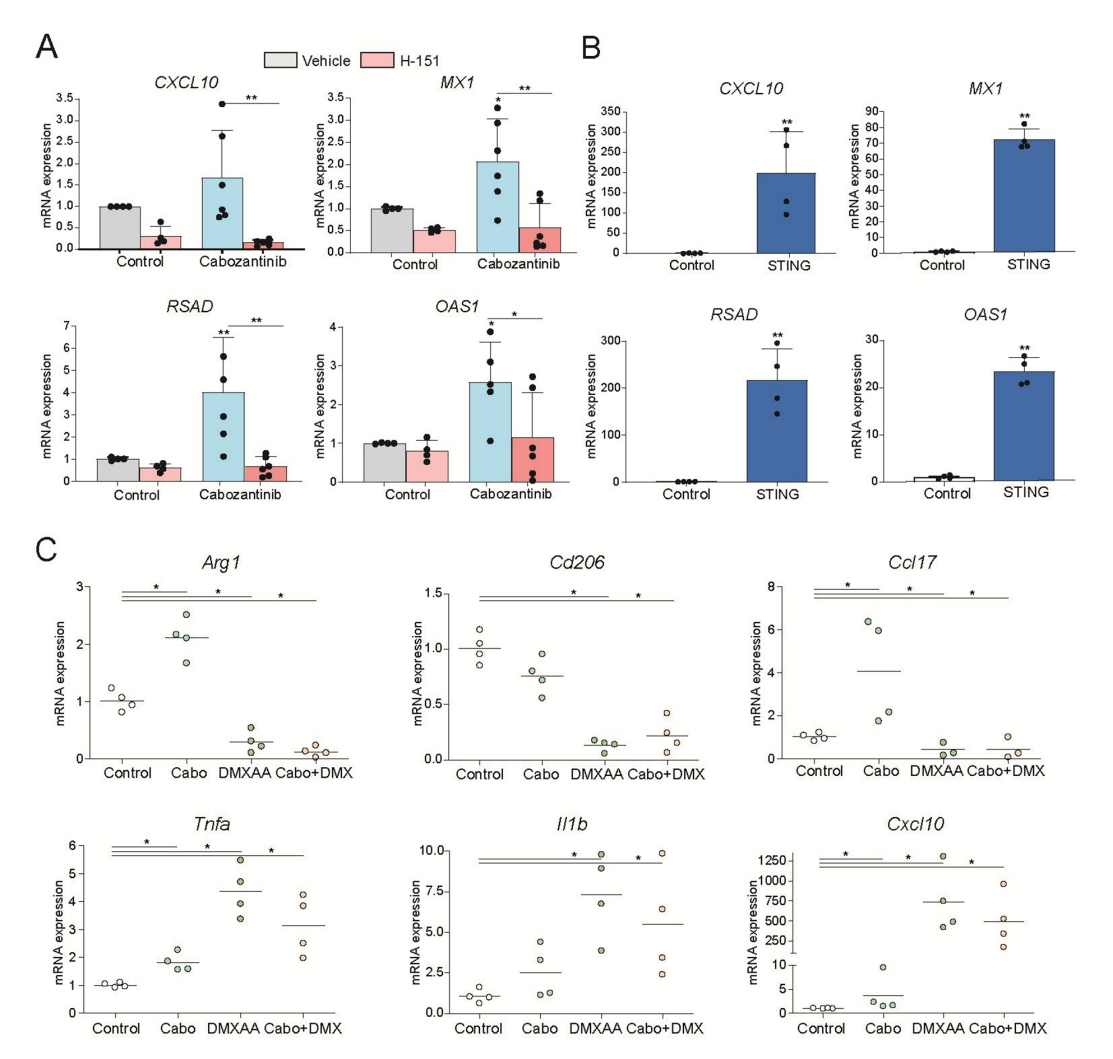
Cabozantinib and STING activation in macrophages favor macrophage reprograming enhancing antitumor immunity. **A** In THP-1–activated macrophages, cabozantinib (5µM) induced expression of type I interferon-stimulated genes (ISGs) *CXCL10*, *MX1*, *RSAD*, and *OAS1*, being abrogated by STING inhibitor H-151 (1µM) (*n* = 3). **B** STING agonist SR-717 (5µM) strongly induction ISGs in THP-1 cells (*n* = 3). **C** Murine peritoneal macrophages were obtained and treated with cabozantinib (10µM) and STING agonist DMXAA. mRNA levels indicative of M1/M2 polarization were determined after 24 h. (*n* = 4). Student t test or one-way ANOVA with a Newman-Keuls post hoc test with *p < 0.05, **p < 0.01. *p < 0.05,

To better understand the implications of STING signaling within the tumor microenvironment, we examined myeloid cells, which play a central role in coordinating innate and adaptive immunity. In THP-1–derived macrophages, cabozantinib treatment induced the expression of type I interferon response genes, indicating that cabozantinib-induced STING activation extends beyond tumor cells to include immune cells within the tumor microenvironment. This induction was abrogated by co-treatment with the selective STING inhibitor H-151, confirming the specificity of the response (Fig. 5A).

Strikingly, STING activation was even more pronounced when THP-1 macrophages were treated with the synthetic STING agonist SR-717 [27], indicating that STING signaling in macrophages can be further pharmacologically amplified (Fig. 5B). Similarly, treatment of murine RAW264.7 macrophages with cabozantinib or the murine STING agonist DMXAA resulted in different degrees of TBK phosphorylation, much higher in DMXAA-treated cells (Supplementary Fig. 4). Consistently, a robust CXCL10 secretion was observed in RAW264.7 cells treated with the STING agonist, further confirming the responsiveness of the STING pathway in innate immune cells (Supplementary Fig. 4). Notably, in both human and mouse macrophages, the magnitude of STING pathway activation achieved with agonists far exceeded that induced by cabozantinib alone.

To gain additional insight into the effect of cabozantinib and STING agonist combination on the tumor’s response and their effectiveness targeting the TME, we isolated peritoneal macrophages and treated them. While cabozantinib effect on macrophages seems to promote a weak induction of an anti-tumor (M1-like) phenotype, DMXAA co-administration greatly potentiated macrophage polarization towards M1-like response as denoted by the strong increases detected in *Cxcl10*, *Il1b* o *TnfF* (Fig. 5C). In contrast, *Ccl17*, *Arg1*, and *Cd206*, all key markers and functional molecules associated with alternatively activated (M2) macrophages, were reduced after treatment with the STING agonist (Fig. 5C). These findings indicate that, while cabozantinib is capable of activating STING signaling in immune cells, this response can be significantly enhanced through pharmacological agonism, supporting the rationale for combining STING agonists with TKIs in immunotherapeutic strategies for HCC.

### STING agonism enhances cabozantinib antitumor activity and promotes cytotoxic T cell infiltration in a syngeneic murine HCC model

Given that our results identify STING as a key mediator of the immunogenic and cytotoxic effects of cabozantinib, supporting STING activation as a combinatorial strategy to enhance TKI therapeutic efficacy, we next evaluated this approach in a syngeneic murine model. Immunocompetent C57BL/6 mice bearing subcutaneous Hepa1-6 tumors were treated for 21 days with cabozantinib, the STING agonist DMXAA, or a combination of both.

**Fig. 6:**
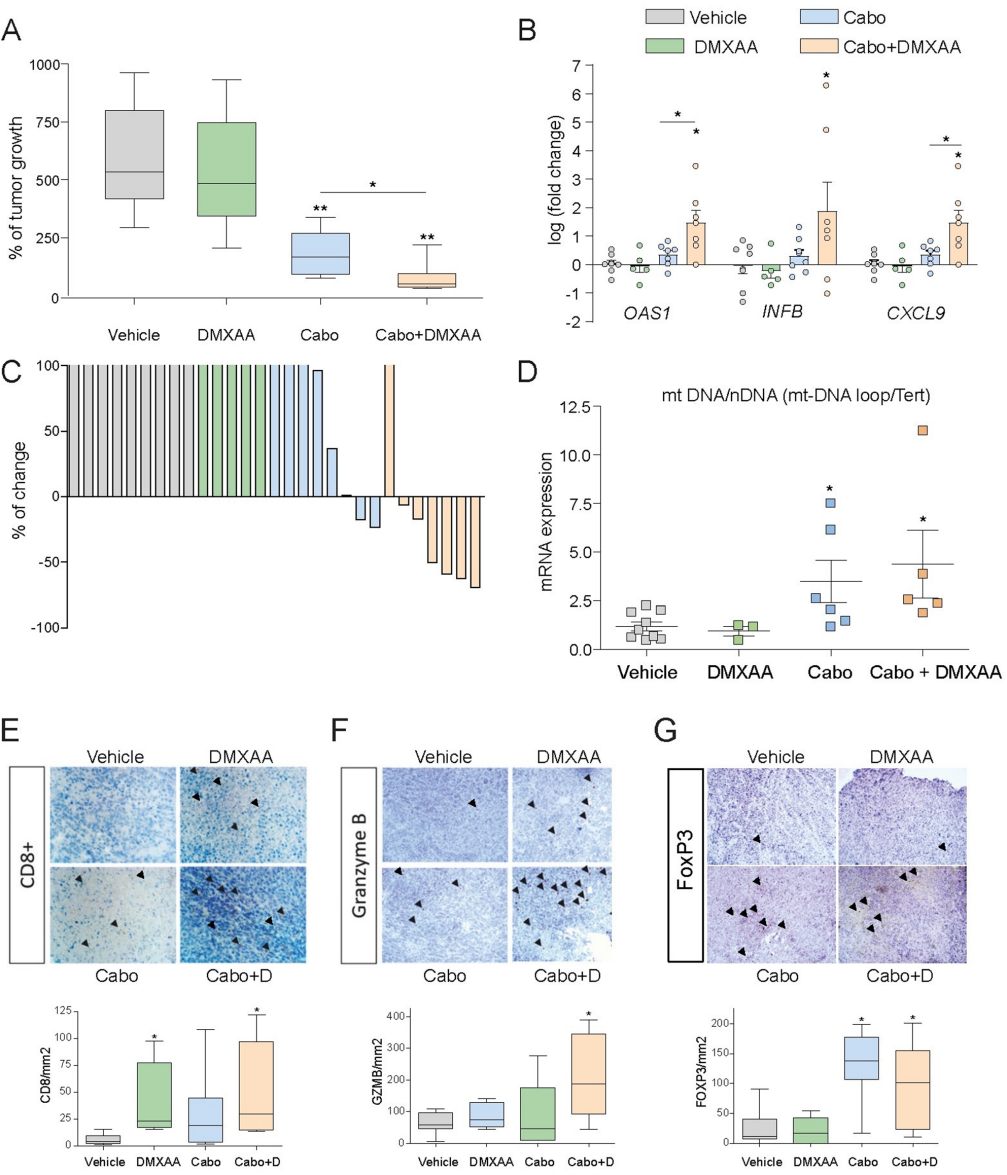
STING activation potentiates cabozantinib-induced anti-tumor efficacy in a murine HCC model enhancing immune cell infiltration and cytotoxic responses in vivo. **A**-**C** In a syngeneic subcutaneous murine HCC model (Hepa1-6), cabozantinib/DMXAA combination induced frequent tumor regression (Veh *n* = 9, DMXAA *n* = 5, Cabo *n* = 8, combo *n* = 7). **B** Gene expression analysis (*Cxcl9*, *Oas1*, *Ifnb1*) in tumor tissue after cabozantinib (10 mg/kg) and DMXAA (10 mg/kg) treatments. **D** Analysis of circulating mtDNA/nDNA ratio by qPCR in serum samples from cabozantinib-DMXAA treated mice (Veh *n* = 9, DMXAA *n* = 3, Cabo *n* = 6, combo *n* = 5). **E** Immunohistochemistry (IHC) staining of Hepa1-6 tumors from cabozantinib/DMXAA-treated mice reveals increased intratumoral CD8⁺ T cell infiltration. **F** Upregulation of Granzyme B expression, indicative of cytotoxic T cell activity after cabozantinib/DMXAA treatment. **G** FOXP3⁺ regulatory T cells (Tregs) increased in cabozantinib-treated tumors. Student t test or one-way ANOVA with a Newman-Keuls post hoc test with *p < 0.05, **p < 0.01

Although cabozantinib monotherapy significantly delayed tumor growth (Fig. 6A and Supplementary Fig. 5), the combination treatment demonstrated superior antitumor efficacy, resulting in tumor regression in six out of seven treated mice (Fig. 6C). Notably, DMXAA alone had no significant effect on tumor growth and did not cause detectable inflammatory infiltration or liver damage, either alone or in combination with cabozantinib, as confirmed by hematoxylin and eosin staining of the liver sections and liver expression of inflammatory genes (Supplementary Fig. 6). To further characterize the underlying mechanisms, we analyzed the excised tumors at the transcriptomic, molecular, and histopathological levels. Gene expression profiling of tumors from cabozantinib-treated mice revealed upregulation of ISGs such as *Cxcl9*, *Oas1*, and *Ifnb1*, an effect that was further amplified in tumors from mice receiving combination therapy with DMXAA (Fig. 6B).

Since cabozantinib induces mitochondrial dysfunction and mtDNA leakage in vitro, and given that circulating mtDNA levels may serve as biomarkers for disease progression and therapeutic response, we next analyzed serum mtDNA levels in mice treated with cabozantinib and DMXAA. To assess this, we first compared the circulating nuclear DNA (nDNA) profile between healthy and tumor-bearing mice, as circulating tumor DNA is commonly used as a surrogate for the tumor burden, and the circulating mtDNA. Total nucleic acids were isolated from mouse serum collected at the time of sacrifice and analyzed by qPCR using specific primers (Table 1) targeting the mitochondrial D-loop (displacement loop) region. Cabozantinib-treated mice showed a significant 3-fold increase in the serum mtDNA/nDNA ratio compared to untreated controls (Fig. 6D).

Immunohistochemical analyses of tumor tissues revealed that cabozantinib treatment led to a 2.5-fold increase in intratumoral CD8 + T cell infiltration, which was further elevated in tumors from the combination-treated mice (Fig. 6E). Consistent with the enhanced cytotoxic immune activity, granzyme B expression significantly increased following combination therapy, as confirmed by IHC staining (Fig. 6F). Interestingly, cabozantinib alone increased FOXP3 + regulatory T cell (Treg) infiltration, which was not further increased after DMXAA co-treatment, suggesting that STING activation improved the immune context by limiting immunosuppressive cell populations (Fig. 6G).

**Fig. 7:**
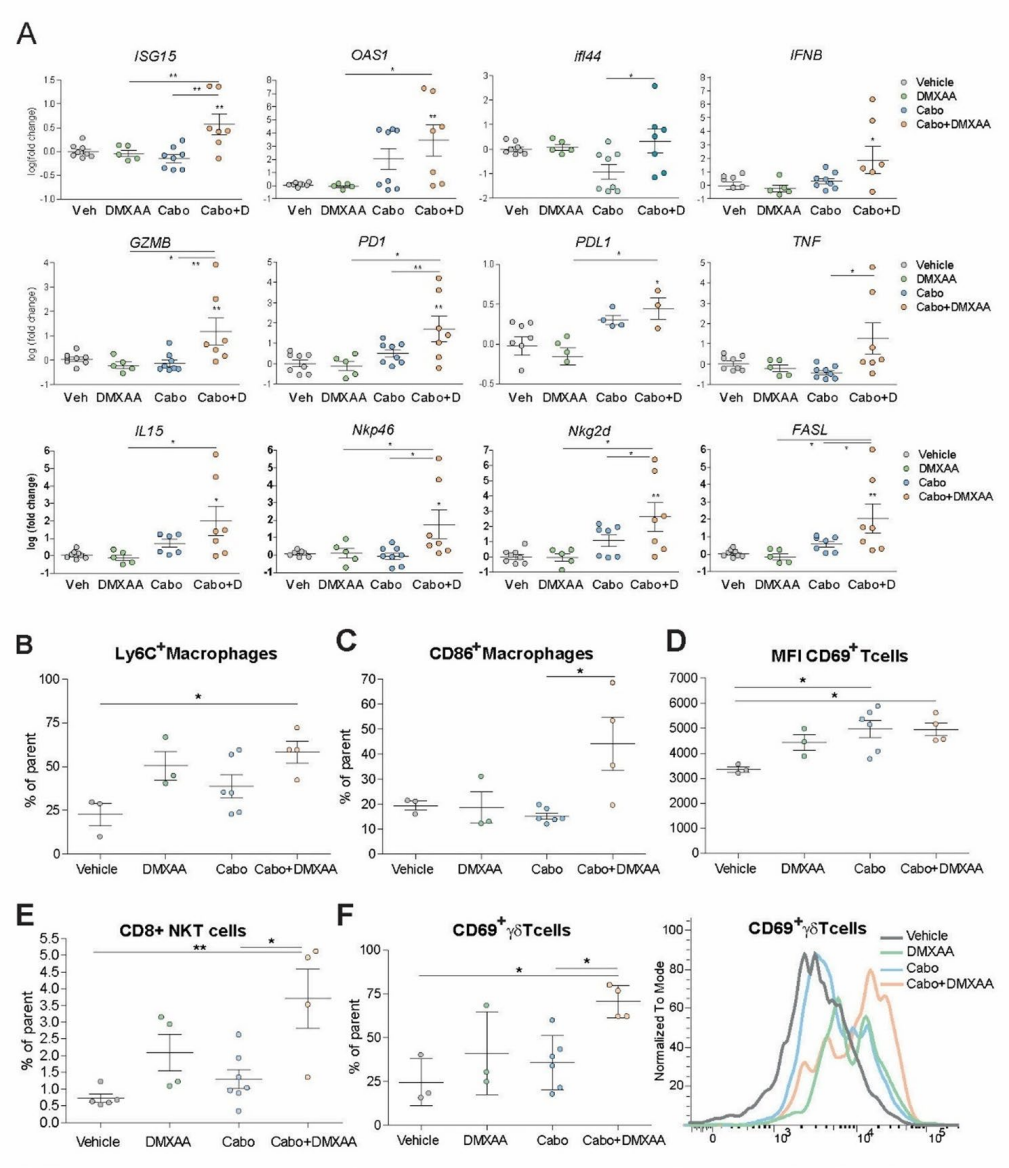
STING activation enhances cabozantinib-induced immune cell infiltration and cytotoxic responses in vivo in a syngeneic subcutaneous murine HCC model (Hepa1-6). **A** Quantitative RT-PCR analysis in tumor tissue of genes related to innate and adaptive cytotoxic responses in cabozantinib-DMXAA-treated mice. Vehicle *n* = 9, DMXAA *n* = 5, Cabozantinib *n* = 8, combination *n* = 7. Student t test or one-way ANOVA with a Newman-Keuls post hoc test with *p < 0.05, **p < 0.01. **B** Frequency of Ly6C⁺ inflammatory macrophages, (**C**) frequency of CD86⁺ macrophages, (**D**) mean fluorescence intensity (MFI) of CD69 on T cells, (**E**) frequency of CD8 + NKT cells, (**F**) frequency of CD69⁺ γδ T cells and representative flow cytometry histogram of CD69 expression on γδ T cells. Data are presented as mean ± SEM; each point represents an individual mouse. Statistical significance was determined by one-way ANOVA followed by Tukey’s post-hoc test (*p < 0.05, **p < 0.01)

Additional qRT-PCR analysis showed increased expression of NK cell markers such as NKp46 and NKG2D, as well as cytolytic effectors including granzyme B, FasL, and TNFα in tumors treated with cabozantinib and DMXAA (Fig. 7A). Notably, PD-L1 transcript levels were elevated in these tumors, possibly reflecting a compensatory feedback mechanism aimed at dampening excessive T cell activation.

Due to the efficacy of the treatments, not much tumor sample was left from the treated animals. Despite that, western blotting analysis on the remaining material allow us to validate an increase in TBK and STING phosphorylation after cabozantinib treatment (Supplemental Fig. 7), and a clear tendency in the Cabo + DMXAA group, in agreement with our in vitro data.

To further define the immune mechanisms underlying the antitumor activity of cabozantinib and its combination with STING agonist, we performed multiparametric spectral flow cytometry analysis (Supplementary Fig. 8) of tumor-infiltrating immune cells in subcutaneous Hepa1-6 tumors after 10 days of treatment. Combination treatment of cabozantinib and DMXAA induced a marked increase in Ly6C⁺ inflammatory macrophages within the tumor microenvironment, indicating enhanced recruitment of monocyte-derived innate immune cells characteristic of an immunogenic inflammatory response (Fig. 7B), a tendency that was also observed in cabozantinib- or DMXAA-treated alone. This was accompanied by a significant rise in CD86⁺ macrophages (Fig. 7C), consistent with functional activation and increased antigen-presenting capacity. In parallel, a robust enhancement of global T-cell activation was observed, as reflected by elevated CD69 on tumor-infiltrating T cells (Fig. 7D) in all treated groups. Moreover, the combination therapy significantly increased the abundance of CD8⁺ NKT cells, an innate-like cytotoxic lymphocyte population that rapidly responds to inflammatory and type I interferon–dependent signals and contributes to early antitumor immune responses (Fig. 7E). Activation of γδ T cells, a population critically involved in early tumor immune surveillance and immunogenic cell death, was further confirmed by representative CD69 expression histograms, showing enhanced activation in the combination-treated group (Fig. 7F). However, while combined treatment was associated with increased expression of NK-associated genes (Fig. 7A) in bulk tumor tissue, flow cytometric analysis did not reveal a significant increase in NK cell frequency or number, suggesting that these changes likely reflect enhanced NK cell activation or transcriptional modulation rather than increased infiltration.

In addition, and supporting this, analysis of T cell memory subsets revealed a treatment-associated reduction in CD8⁺ central memory T cells across all treatment groups relative to vehicle, consistent with mobilization and differentiation toward effector phenotypes, whereas CD4⁺ central memory T cells were selectively increased in the combination group, suggesting productive helper T-cell priming and the establishment of long-term immune memory (Supplementary Fig. 9). Altogether, these data demonstrate that cabozantinib combined with STING agonism induces a coordinated innate and adaptive immune activation program consistent with robust immunogenic, pro-inflammatory, and durable antitumor immunity. Together, these findings demonstrate that cabozantinib induces mitochondrial damage and promotes the release of mtDNA, a key trigger for the activation of the STING pathway in vivo. STING activation contributes to antitumor immunity, inflammatory myeloid recruitment, enhancing antigen presentation, and driving robust activation of cytotoxic lymphocyte populations. Moreover, STING agonism further amplified these effects, supporting its potential as a combinatorial strategy to improve the efficacy of cabozantinib in HCC treatment.

### Cabozantinib induces systemic immune remodeling and cytotoxic signaling in HCC patients, revealing potential biomarkers of response

**Fig. 8:**
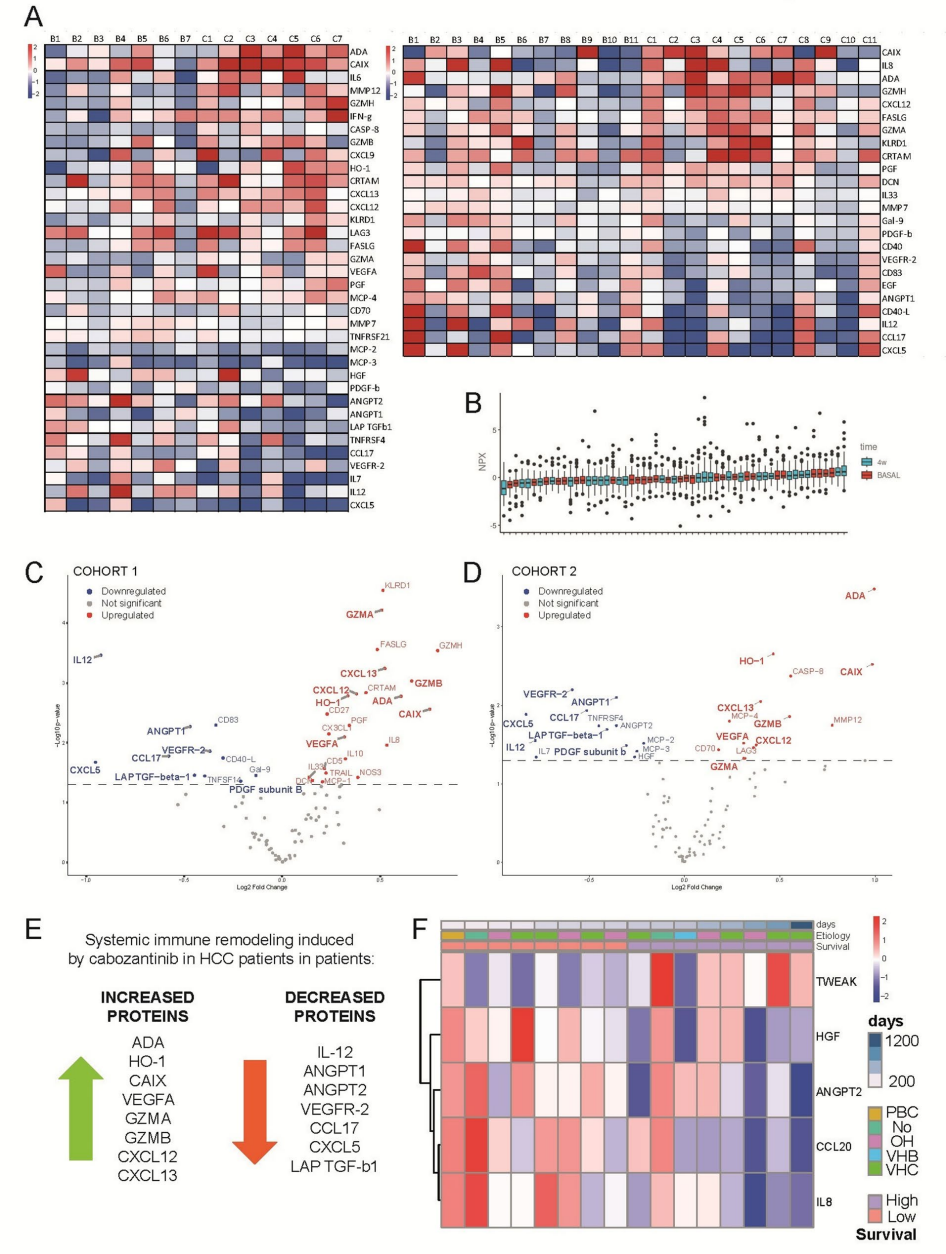
Cabozantinib induces systemic immune remodeling and cytotoxic signaling in HCC patients. **A** Heatmap (Z-score normalization) of significantly altered serum proteins in Cohort 1 (*n* = 7; patients previously treated with sorafenib, left) and Cohort 2 (*n* = 11; sorafenib-intolerant patients, right). Serum samples were collected before and one month after initiation of cabozantinib. **B** Comparison of normalized protein expression (NPX) values between cohorts. **C**, **D** Volcano plot of serum proteomic changes following cabozantinib (4 weeks). Coincident proteins, squared. **E** Schematic summary of cabozantinib-induced systemic immune changes. Paired Student t test, volcano plot threshold *p* < 0.05. **F** Heatmap of serum proteins with basal levels associated with cabozantinib efficacy

To characterize cabozantinib-induced systemic immune remodeling and explore its potential synergy with STING agonists, we performed multiplex proteomic profiling of serum samples from two independent cohorts of patients with advanced HCC using the Olink^®^ Immuno-Oncology panel (Fig. 8A, left and right images). Normalized protein expression (NPX) values were comparable between the two cohorts, validating the consistency of the results across different patient groups and supporting reliable data integration (Fig. 8B). In the first cohort (Fig. 8A left), with seven patients with advanced HCC previously treated with sorafenib, we analyzed serum samples collected one month after cabozantinib initiation and visualized the results using a Volcano plot (Fig. 8C). Proteomic analysis revealed significant increases in immune- and inflammation-related proteins, including adenosine deaminase (ADA), carbonic anhydrase IX (CAIX), caspase-8 (CASP8), CXCL13, granzyme B (GZMB), heme oxygenase-1 (HO-1), monocyte chemotactic protein-4 (MCP-4), and matrix metalloproteinase-12 (MMP12). These changes are consistent with enhanced cytotoxic immune activity, cellular stress, and hypoxia-associated signaling. In parallel, we observed decreases in several factors linked to angiogenesis and immunosuppression, including angiopoietin-1 (ANGPT1), CCL17, CXCL5, TNFRSF4 (OX40), and vascular endothelial growth factor receptor 2 (VEGFR-2).

In a second independent cohort (Fig. 8A right), with eleven patients who initiated cabozantinib due to intolerance to sorafenib, we observed a similar proteomic profile one month after treatment (Fig. 8D). Increases were noted in ADA, CAIX, CXCL12, CXCL13, FAS ligand (FASLG), granzymes A, B, and H (GZMA, GZMB, and GZMH), HO-1, and placental growth factor (PGF), consistent with ongoing cytotoxic immune activation and cellular stress. Concurrently, the levels of ANGPT1, CD83, interleukin-12 (IL-12), and VEGFR-2 were reduced, supporting a shift away from immunosuppressive and angiogenic signaling.

When combining data from both cohorts, we observed consistent and significant changes, including increases in HO-1, ADA, CAIX, VEGFA, GZMA, GZMB, CXCL12, and CXCL13, and decreases in IL-12, ANGPT1/2, VEGFR-2, CCL17, CXCL5, and LAP TGF-β1, as reflected in this schematic figure (Fig. 8E). These serum-based immune signatures, consistent with the STING-driven cytotoxic immune responses and apoptotic priming observed, support cabozantinib’s ability to remodel the tumor microenvironment toward a more immunostimulatory and antiangiogenic phenotype.

To identify potential biomarkers of clinical benefit, we stratified patients based on treatment outcomes. Among the proteins most relevant to cabozantinib efficacy, CCL20, HGF, IL8, TWEAK, and ANGPT2 emerged as key factors, reflecting their established roles in angiogenesis, inflammation, and immune regulation (Fig. 8F). Individual analyses for each marker showed that higher levels of CCL20, HGF, IL8 and ANGPT2 are more common among patients with lower survival times, while TWEAK was positively associated with survival (Supplementary Fig. 10). Consistently, data from The Human Protein Atlas indicate that CCL20, IL8 and ANGPT2 are considered prognostic markers in HCC, in line with our findings (Supplementary Fig. 11). In contrast, TWEAK, associated to better prognosis, and HGF did not reach enough significance for prognostic value in the global HCC population, suggesting that they may be more specific indicators of cabozantinib’s efficacy.

Notably, these proteins associated with poor prognosis under cabozantinib therapy were not significantly altered in a separate cohort of eight patients treated with atezolizumab–bevacizumab who developed severe intolerance to immunotherapy (Reig et al., unpublished). Since intolerance to immunotherapy often indicates poor clinical benefit, this observation suggests that the identified serum markers may represent cabozantinib- or TKI-specific response signatures rather than general prognostic indicators.

In summary, our proof-of-concept study revealed consistent systemic immune remodeling in HCC patients treated with cabozantinib across two independent cohorts. Importantly, the observed modulation of angiogenic and inflammatory mediators, together with increased cytotoxic effectors, aligns with our experimental data showing mitochondrial damage–induced STING pathway activation. These findings support the concept that cabozantinib efficacy is partly mediated by STING-driven immune remodeling, and they provide clinical rationale for exploring combinatorial regimens with STING agonists to enhance therapeutic outcomes in HCC.

## Discussion

Immunotherapy has transformed the therapeutic landscape of advanced hepatocellular carcinoma (HCC) over the past decade [1, 2]. However, nearly 70% of patients eventually discontinue immune checkpoint inhibitor (ICI) therapy owing to disease progression or immune-related toxicity, highlighting the urgent need for effective post-ICI treatment strategies [28, 29], an area that remains largely unexplored. In this context, TKIs such as cabozantinib are frequently employed, although the clinical outcomes remain modest [9]. A deeper understanding of the immunological mechanisms underlying both activity and resistance to TKIs could facilitate the development of evidence-based drug combinations aimed at addressing this unmet clinical need.

The cGAS/STING (cyclic GMP-AMP synthase/Stimulator of Interferon Genes) pathway has been implicated in liver inflammation and fibrosis and may also influence HCC development [30–33]. Here, we identified mitochondrial stress and STING activation as key mechanisms through which cabozantinib enhances tumor immunogenicity and promotes antitumor immune responses in HCC. We show that cabozantinib disrupts mitochondrial integrity and induces membrane depolarization, reactive oxygen species (ROS) production, and cytosolic release of mitochondrial DNA (mtDNA). These events activate the cGAS/STING signaling cascade, as evidenced by TBK1 and IRF3 phosphorylation and induction of interferon-stimulated genes (ISGs). Interestingly, mitochondrial damage has also been reported for other TKIs, including sorafenib and regorafenib [23–25], where it has been linked to inflammasome activation [34]. These findings raise the possibility that mitochondrial disruption may occur more broadly with antiangiogenic therapies, potentially contributing to immune activation and microenvironmental remodeling through different mechanisms. Consistent with this notion, we observed upregulation of ISGs after lenvatinib and sorafenib exposure, suggesting that mitochondrial-dependent STING activation might also play a role in the activity of some TKIs in HCC therapy, although this requires further validation.

Pharmacological STING activation using the murine agonist DMXAA further amplified cabozantinib’s effects and may facilitate the involvement of other immune cell types [35]. In immunocompetent murine models, pharmacological STING stimulation using DMXAA synergized with cabozantinib to enhance antitumor activity. Combination therapy led to a pronounced increase of inflammatory macrophages and upregulation of cytotoxic molecules (granzyme B, FasL, TNFα), and expression of CD69 demonstrating robust engagement of both adaptive and innate immunity. While NK-associated transcripts were increased in bulk tumor analysis, this was not accompanied by a measurable increase in NK cells by flow cytometry, probably reflecting that NK receptor expression can be dynamically regulated on a per-cell basis. Although DMXAA alone showed limited efficacy, its capacity to remodel the tumor immune microenvironment enhanced the immunostimulatory effect of cabozantinib. These results underscore the potential synergy between mitochondrial stress and STING activation as a strategy to reprogram the tumor microenvironment.

Cabozantinib treatment induced ICD in HCC cells, as evidenced by HMGB1 and ATP secretion, which may contribute to tumor microenvironment (TME) remodeling. Similar effects have been reported in murine prostate cancer, where cabozantinib increased HMGB1 and CXCL12 levels, promoting neutrophil infiltration [36], consistent with observations in HCC models with cabozantinib used in combination with anti-PD1 [37]. ICD is known to activate cytotoxic T lymphocytes and establish immunological memory [38]. Indeed, cabozantinib-treated murine HCC tumors, especially in combination with anti-PD1, increased neutrophil infiltration and reduced intratumor CD8 + PD1 + T-cell proportions [37], thereby improving overall anti-tumor response. While our study did not specifically address the role of neutrophils in this context, our findings support a broader immunomodulatory effect of cabozantinib mediated through mitochondrial stress and STING activation. However, compensatory upregulation of immunosuppressive factors, including PD-L1 and FOXP3⁺ regulatory T cells, has also been observed following cabozantinib monotherapy, highlighting the need for rational combination strategies to overcome adaptive immune resistance. Similar adaptive resistance has been documented in other “cold” tumor types, such as pancreatic [39, 40] and glioblastoma [41, 42] cancers, where the initial immune activation is often counterbalanced by feedback inhibition. These findings support the combination of STING agonists and TKIs, such as cabozantinib, with ICI therapy to sustain antitumor immune responses. Although DMXAA is limited to murine models due to its species specificity [43], the development of clinically viable human STING agonists will be crucial for translating these insights into effective therapies for HCC. Although we observed no overt hepatotoxicity or systemic toxicity attributable to cabozantinib ± DMXAA at the doses used, combination regimens involving cabozantinib and STING agonists may increase the risk of immune-related adverse events. Although their implementation will require careful toxicity monitoring and close collaboration between liver oncologists and multidisciplinary clinical teams, this would be similar to the approach already established for managing immune-related toxicities under current ICI treatments. In addition, dissecting the relative contribution of tumor-intrinsic versus immune-cell STING signaling will be important for anticipating potential issues that may arise during therapies involving STING agonists. Follow-up studies using immune-cell depletion strategies and conditional STING knockout models will be essential to more clearly delineate the roles of each compartment.

To validate the translational relevance of our findings, we analyzed the serum proteomic profiles of advanced HCC patients treated with cabozantinib from two independent cohorts: (1) sorafenib-treated and (2) sorafenib-intolerant. Proteomic alterations closely align with the key features of STING pathway activation. Increases in granzyme A and B (GZMA and GZMB), CXCL12, and CXCL13 reflect enhanced cytotoxic lymphocyte activity and chemotactic signaling, both downstream of type I interferon responses [44, 45]. Elevated levels of heme oxygenase-1 (HO-1), adenosine deaminase (ADA), and carbonic anhydrase IX (CAIX) indicate oxidative and metabolic stress, which trigger mtDNA release and innate immune activation via cGAS/STING [46]. Conversely, downregulation of angiogenic factors (ANGPT1/2 and VEGFR-2) and immunosuppressive mediators (IL-12, CCL17, CXCL5, and LAP TGF-β1) is consistent with STING-driven reprogramming of the tumor microenvironment by cabozantinib toward an inflamed, less tolerogenic state [47].

Importantly, we found that baseline enrichment of pro-angiogenic and immunosuppressive mediators was associated with clinical responses to cabozantinib, with CCL20, HGF, IL8, TWEAK, and ANGPT2 being the most significant. Of special interest: CCL20, a chemokine known to recruit immunosuppressive regulatory T cells via CCR6, is implicated in immune evasion mechanisms in HCC [48]; HGF (hepatocyte growth factor) is the ligand for MET, a key target of cabozantinib, which promotes tumor progression and resistance; and IL-8, a potent neutrophil chemoattractant, has been shown to promote the recruitment of myeloid-derived suppressor cells (MDSCs) and tumor-promoting macrophages, thereby dampening antitumor immunity. These observations suggest that selective immune engagement, rather than global inflammation, motivates effective cabozantinib responses and highlight these proteins as potential biomarkers. Related to this point, potential confounding effects of prior systemic therapies and disease stage on circulating immune signatures cannot be excluded, underscoring the need to evaluate larger cohorts of patients treated with cabozantinib or other TKIs to better identify serum markers specifically associated with TKI efficacy.

Serum-based biomarker discovery for systemic therapies in HCC management is particularly important. Advanced HCC patients, most of them with comorbidities and risk of hemorrhages, are frequently not biopsied after treatments. Therefore, identifying novel molecules that may guide therapy selection is highly relevant. In this sense, consistent with our in vitro findings, cabozantinib-treated mice exhibited increased circulating mitochondrial DNA (mtDNA), supporting its potential as a non-invasive biomarker of mitochondrial stress and STING pathway activation [49]. However, due to the inherent lability of mtDNA, our observations, derived from fresh plasma in our murine cancer model, could not be replicated in our frozen human serum samples. Future validation in prospectively collected human samples will be pursued, as our murine observations align with emerging evidence indicating that tumor-derived mtDNA, including oxidized or fragmented species, modulates immune sensing, drives inflammation, and influences therapeutic responses. Aberrant mtDNA fragmentomic patterns [50], already documented in cancer, can provide therapy-induced fingerprints with diagnostic or prognostic relevance. Cancer-associated mtDNA alterations, including copy number changes and oxidative modifications, can modulate immune signaling, metabolism, and apoptotic susceptibility [51]. Similarly, oxidized mtDNA may engage both the STING and inflammasome pathways [52], leading to transient antitumor immunity or, in some contexts, chronic immunopathology. Identifying TKI-induced mtDNA alterations could help to refine biomarker strategies and guide personalized treatment approaches.

## Conclusions

In summary, cabozantinib activated mitochondrial stress responses and the STING pathway, reshaping the immune landscape in HCC through enhanced cytotoxicity, inflammatory remodeling, and modulation of immunosuppressive and angiogenic signals. These effects are mirrored in patients and associated with clinically meaningful outcomes. Our data provide a strong mechanistic rationale for combining cabozantinib with STING agonists and/or immune checkpoint inhibitors and identify candidate biomarkers, including mtDNA and soluble immune factors, that may guide precision therapy in liver cancer; however, the therapeutic benefit of these combinations and the clinical validity of these biomarkers require confirmation in larger, prospective clinical studies due to the limited size, heterogeneity, and observational nature of the current patient cohorts.

## Supplementary Information

Supplementary Material 1.

Supplementary Material 2.

Supplementary Material 3.

## Data Availability

Data and materials from this study are available upon reasonable request from the corresponding authors (Anna Tutusaus or Albert Morales).

## Abbreviations

ADA: Adenosine Deaminase
ANGPT1: Angiopoietin-1
CAIX: Carbonic Anhydrase IX
CASP8: Caspase-8
cGAS/STING: cyclic GMP-AMP synthase/Stimulator of Interferon Genes
DAMPs: Damage-Associated Molecular Patterns
ddC: 2’,3′-Dideoxcytidine
DHE: Dihydroethidium
D-Loop: Displacement Loop
FASLG: FAS Ligand
GZMA: Granzymes A
GZMB: Granzyme B
GZMH: Granzymes H
HCC: Hepatocellular Carcinoma
HGF: Hepatocyte Growth Factor
HO-1: Heme Oxygenase-1
ICD: Immunogenic Cell Death
ICI: Immune Checkpoint Inhibitor
IHC: Immunohistochemical Staining
IFN: Type I Interferon
ISG: Interferon-Stimulated Genes
MCP-4: Monocyte Chemotactic Protein-4
MMP12: Matrix Metalloproteinase-12
mtDNA: Mitochondrial DNA
nDNA: Circulating Nuclear DNA
PGF: Placental Growth Factor
PMA: 12-Myristate 13-Acetate
PMS: Peritoneal Macrophages
ROS: Reactive Oxygen Species
TKI: Tyrosine Kinase Inhibitor
TME: Tumor Microenvironment
VEGFR-2: Vascular Endothelial Growth Factor Receptor 2
