# Supplementary material for: Immune remodeling via mitochondria-dependent STING activation enhances cabozantinib response in hepatocellular carcinoma
Source: J Exp Clin Cancer Res. 2026 Jan 9;45:42. doi: 10.1186/s13046-025-03632-z (PMC12882139; doi:10.1186/s13046-025-03632-z)
Supplement: Supplementary file 1 — Supplementary Material 1. [file 13046_2025_3632_MOESM1_ESM.pdf]

IMMUNE REMODELING VIA MITOCHONDRIA-DEPENDENT STING ACTIVATION ENHANCES CABOZANTINIB RESPONSE IN HEPATOCELLULAR CARCINOMA

Patricia Rider[1,2,3,4,5 *], Anna Tutusaus[1,2,3,4 * #], Carlos Cuño-Gómiz[1,2,5], Flavia Savino[1,2,5], Aida Marsal[2,3,4], Neus Llarch[2,3,4], Gemma Iserte[2,3,4], Anna Colell[1,2,6], Pablo García de Frutos[1,2,7,8], Tania Hernáez-Alsina[9], Marco Sanduzzi-Zamparelli[2,3,4,10], Montserrat Marí[1,2,3,4], María Reig[2,3,4,10], Albert Morales[1,2,3,4 #]

SUPPLEMENTAL FIGURES

Supplemental Figure 1. A) Cabozantinib (50µM) induced-TBK1 phosphorylation was suppressed in BCLC5 cells after mtDNA depletion with ddC (150µM) for 48 h. (n=2). Cabozantinib induced the mRNA levels of *MX1*, *RSAD2* and *CLCX10* in BCLC5 cells. (n=3). B) Cabozantinib (50µM) induced-TBK1 phosphorylation was suppressed in PLC/PRF/5 cells after mtDNA depletion with ddC (150µM) for 48 h. (n=2) *p<0.05.

A

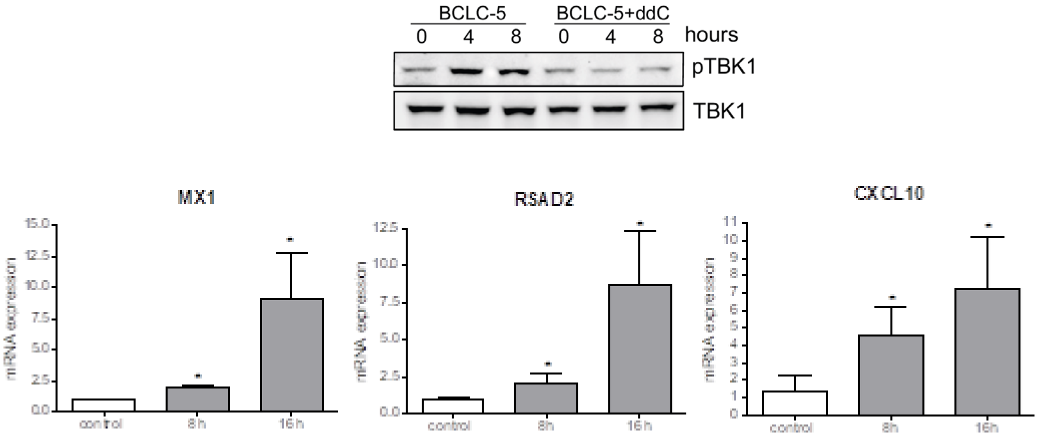

B

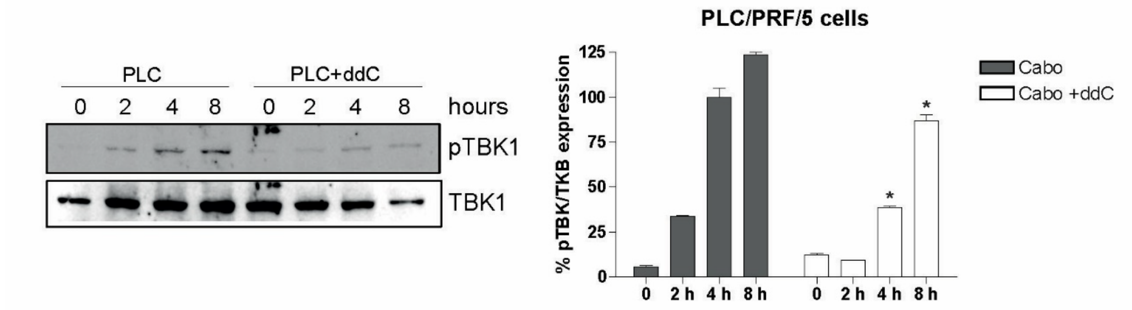

Supplemental Figure 2. Upper panel, reduction in STING expression by CRISPR-Cas9 editing in Hep3B cells confers protection against cabozantinib-induced cytotoxicity (n=3) *p<0.05. Lower panel, reduction in STING expression by CRISPR-Cas9 editing in Hep3B cells reduces TBK phosphorylation and reduces cabozantinib (5 μM, 72 hours) effect on tumor growth of Hep3B spheroids. (n=3) *p<0.05.

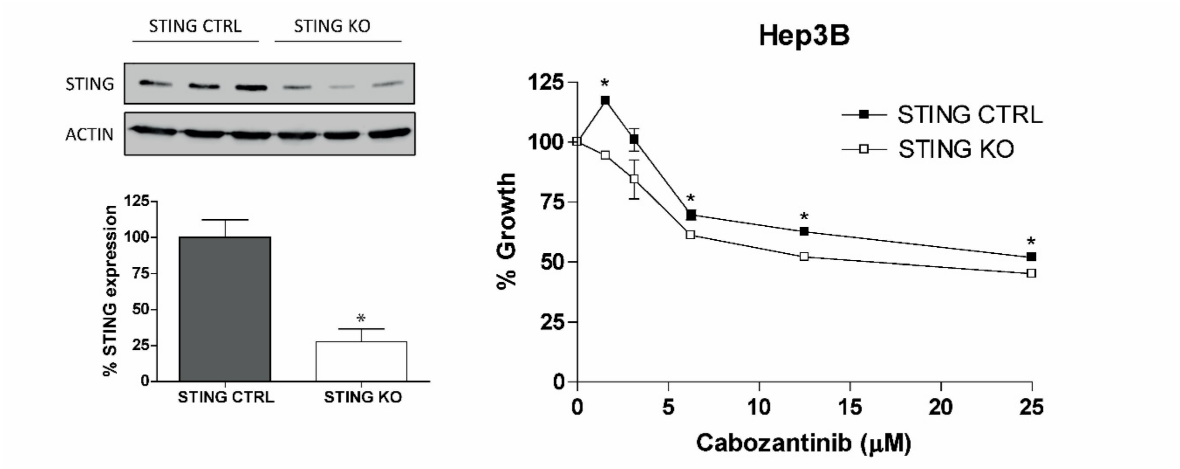

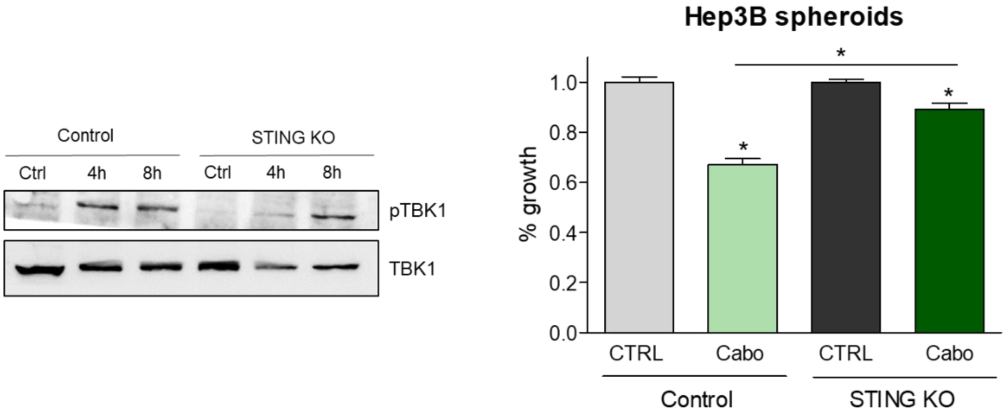

Supplemental Figure 3. STING activation increases lenvatinib-induced cell death in hepatoma cells. A) Hepa1-6 cells were treated with lenvatinib and the murine STING agonist DMXAA (100μM) and cell viability analyzed by MTT assay at 24h. A) Hep3B cells were treated with lenvatinib and the STING agonist SR-717 (5μM) and cell viability analyzed by MTT assay at 24h. (n=2) *p<0.05 vs. control cells.

A

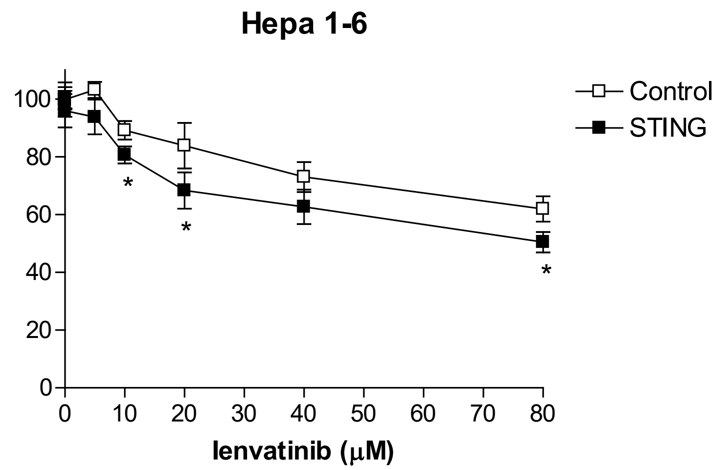

B

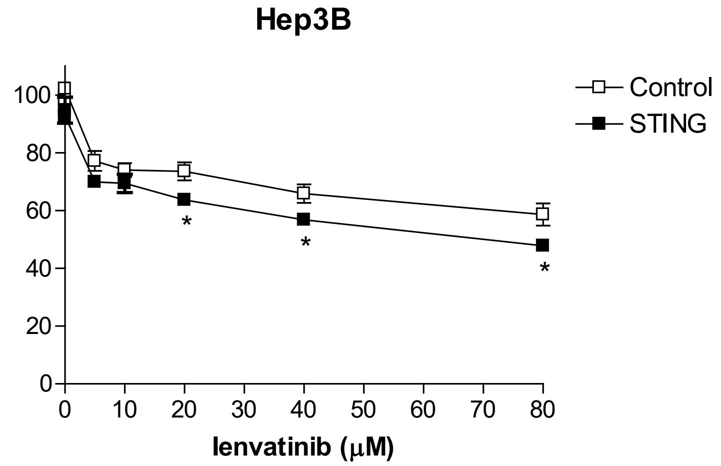

Supplemental Figure 4. TBK phosphorylation in RAW264.7 macrophages treated with cabozantinib (50µM) or the murine STING agonist DMXAA (100µM). CXCL10 secretion in RAW264.7 cells treated with the STING agonist or cabozantinib. (n=2) *p<0.05 vs. control cells. #p<0.05 vs. cabozantinib-treated cells.

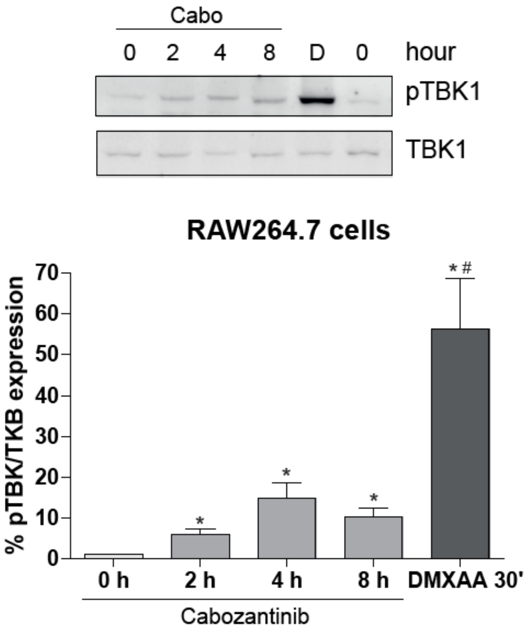

Supplemental Figure 5. Tumor growth of subcutaneous Hepa1-6 tumors in immunocompetent mice treated with cabozantinib and STING agonist DMXAA. (Veh n=9, DMXAA n=5, Cabo n=8, combo n=7). *p<0.05, **p<0.01.

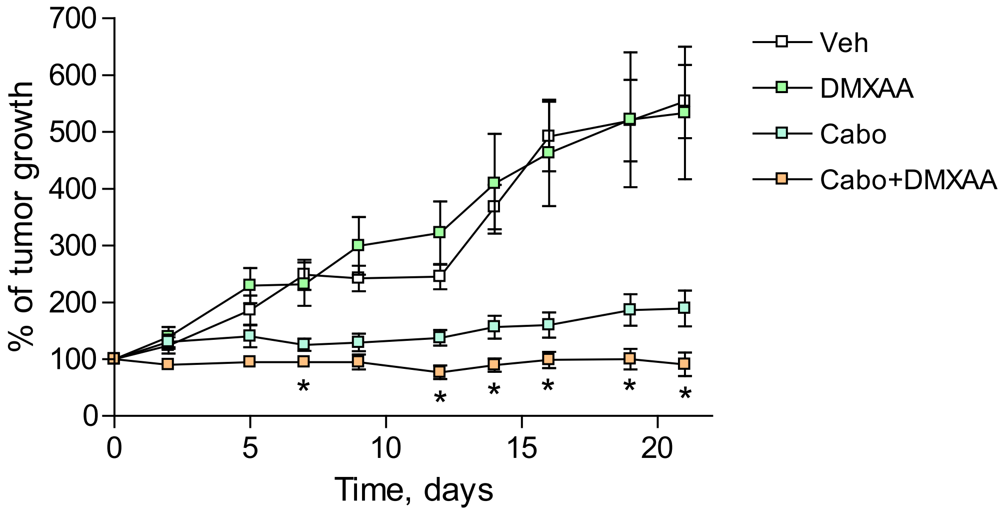

Supplemental Figure 6. H&E staining (left) and mRNA expression (right) of liver biopsies from mice treated with vehicle, DMXAA and/or cabozantinib during the immunocompetent murine model.

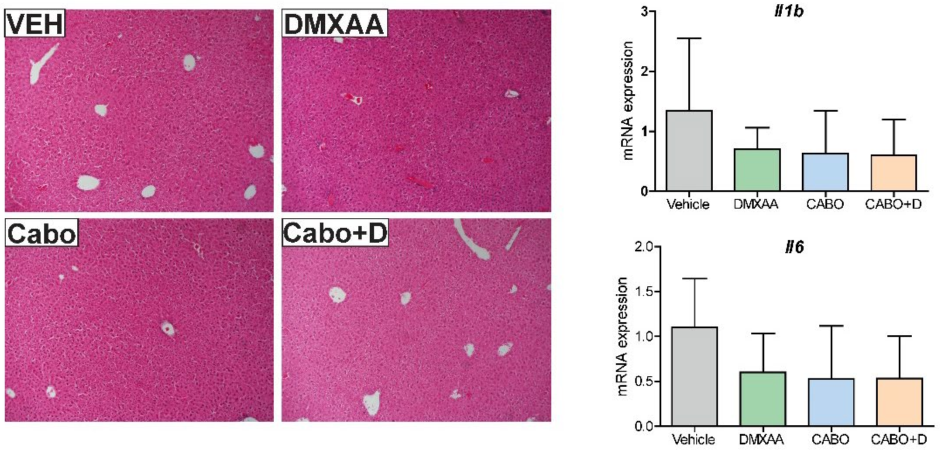

Supplemental Figure 7. Western Blot analysis of Hepa1-6 tumor samples from cabozantinib/DMXAA-treated mice. Images from each group were obtained and quantified using Image J software. (n=4), *p<0.05.

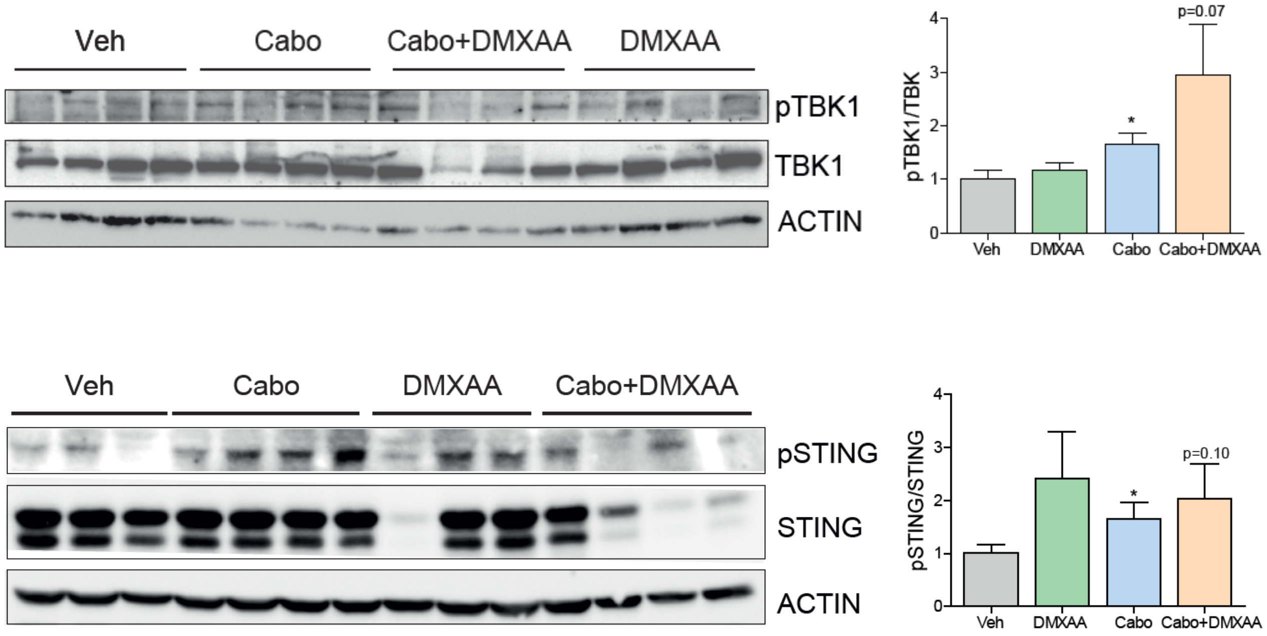

Supplemental Figure 8. Representative image of the gating strategy used for multiparametric spectral flow cytometric analysis.

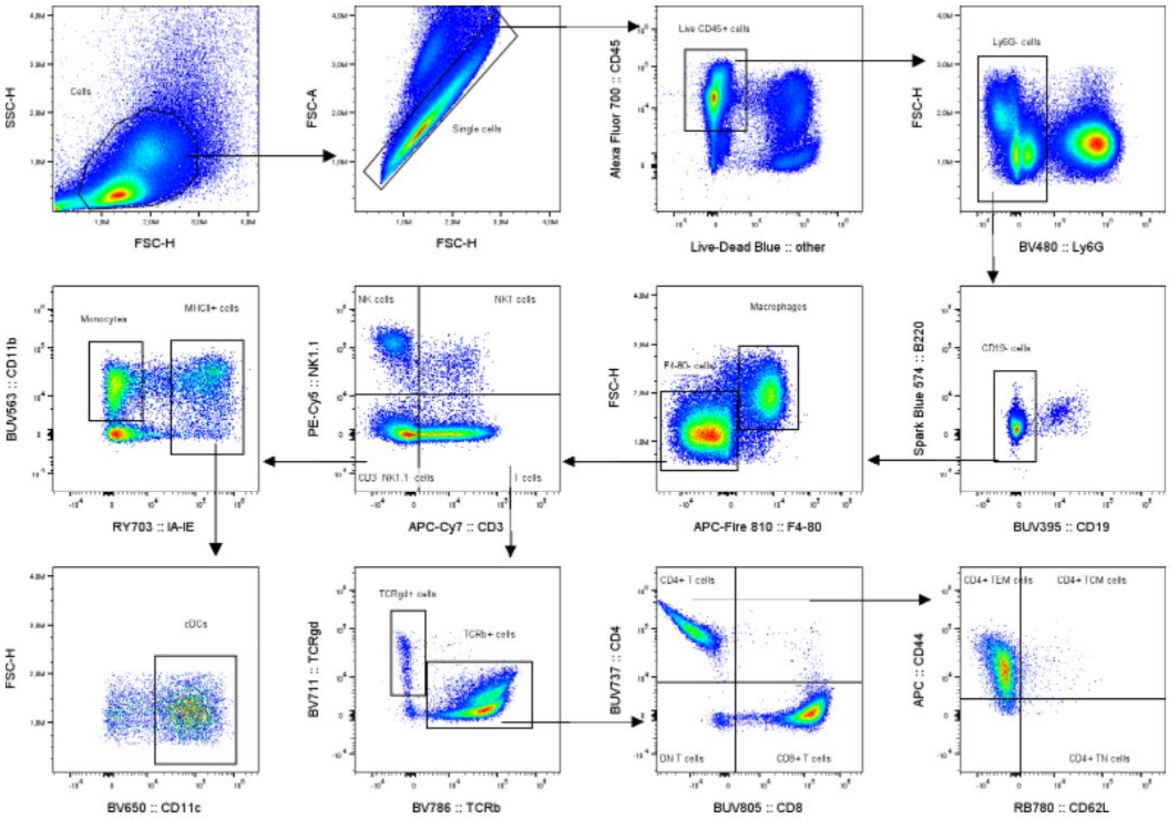

Supplemental Figure 9. Frequency of CD8⁺ or CD4+ central memory T cells. Data are presented as mean ± SEM; each point represents an individual mouse. Statistical significance was determined by one-way ANOVA followed by Tukey's post-hoc test (*$p < 0.05$).

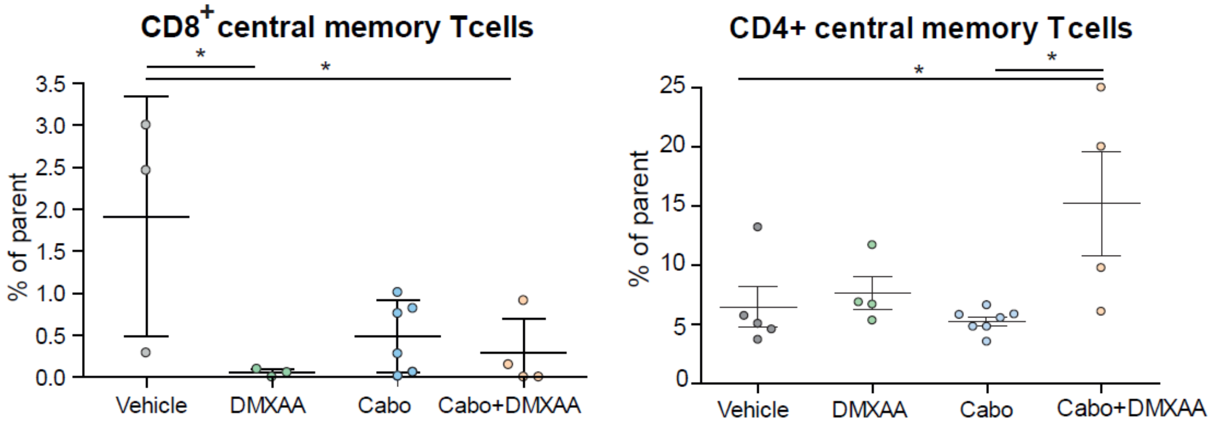

Supplemental Figure 10. Individual representation of CCL20, IL8, ANGPT2, HGF and TWEAK levels vs. survival for each analyzed patient.

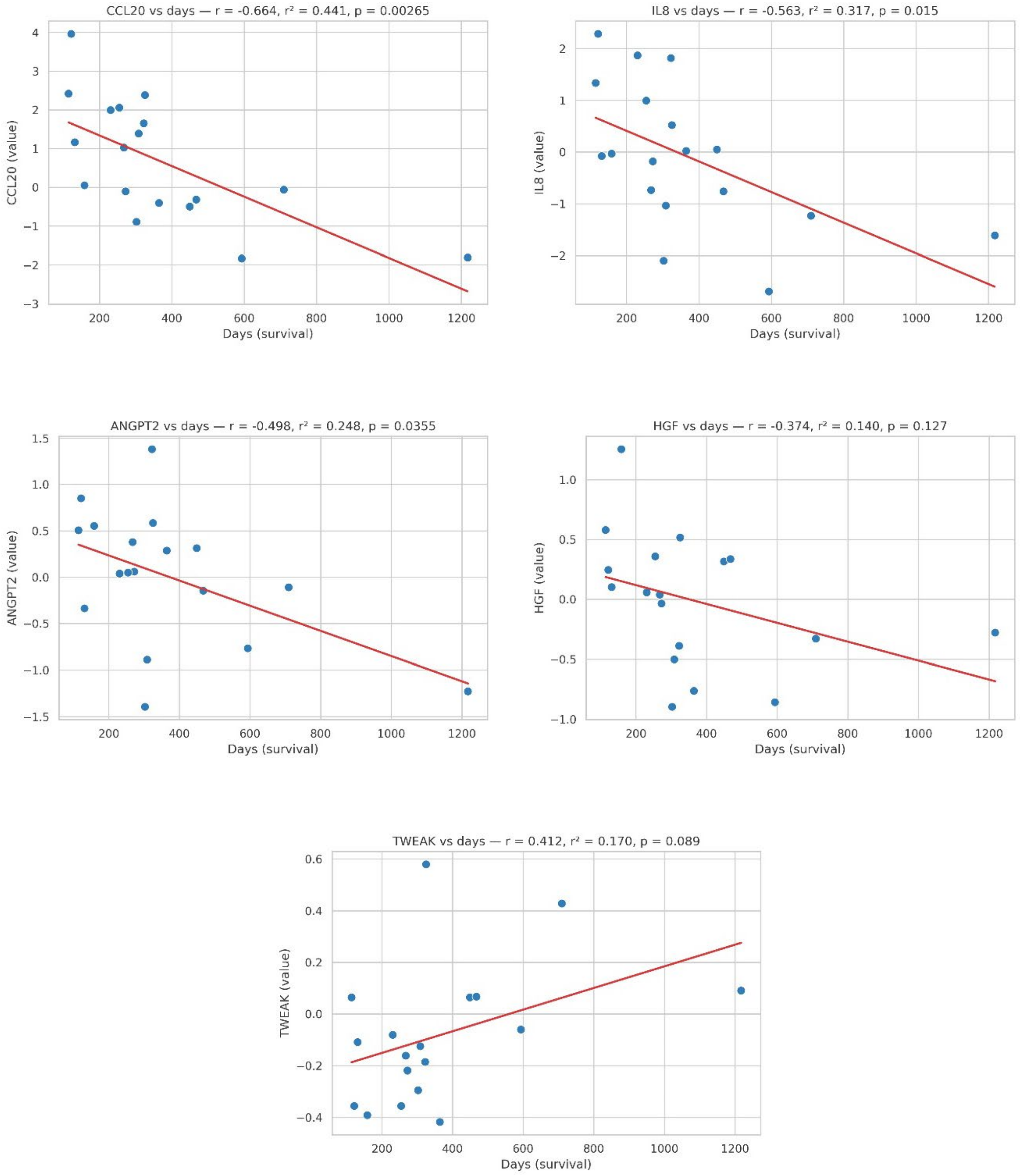

Supplemental Figure 11. CCL20, IL8, ANGPT2, HGF and TWEAK data for HCC patients obtained from The Human Protein Atlas webpage.

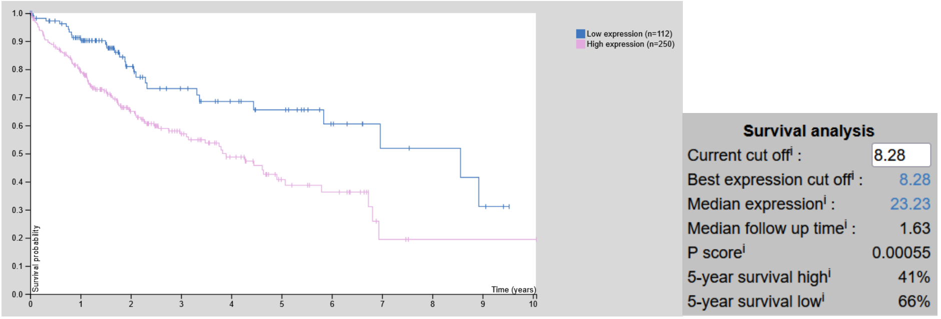

CCL20

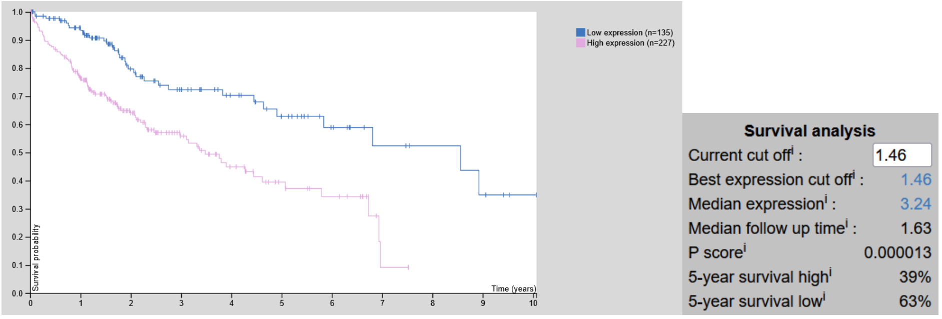

IL8

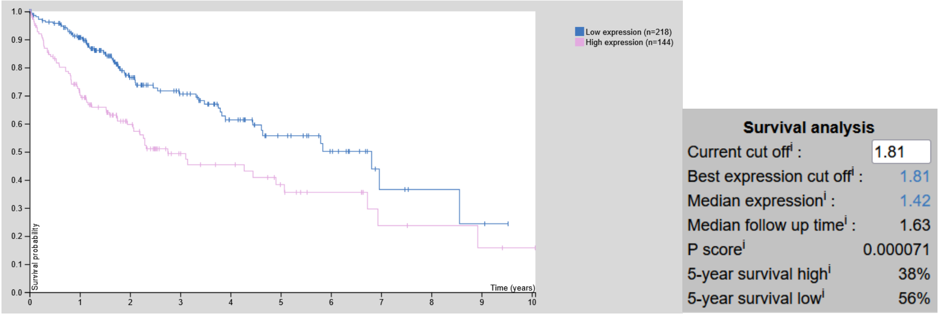

ANGPT2

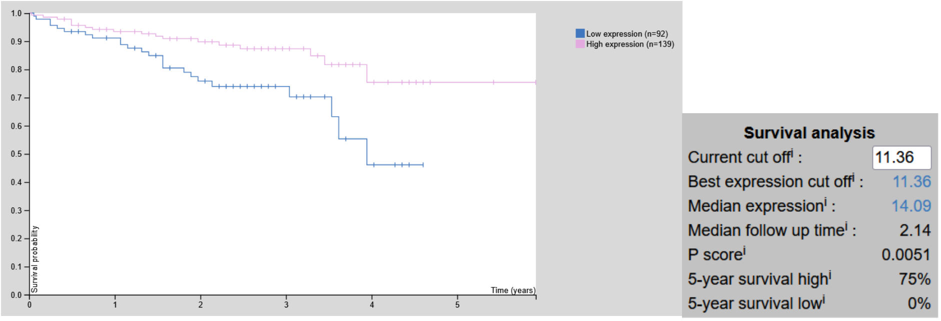

TWEAK

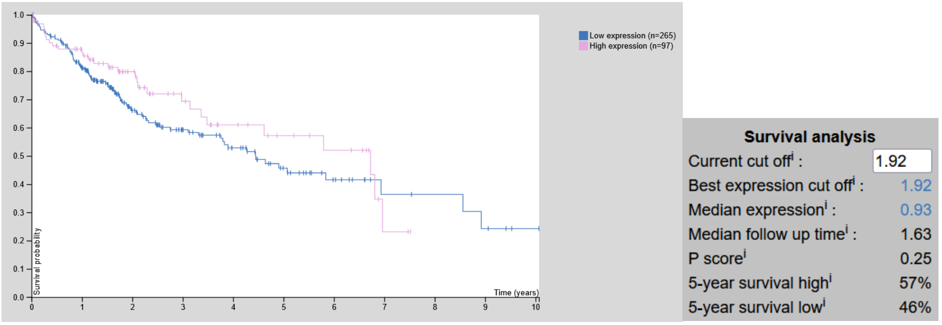

HGF

Supplemental Table 1.

Antigen	Conjugate	Clone	Cat. number (manufacturer)	Dilution
**CD69**	SuperBright 600	clone H1.2F3	#63-0691-82 (Invitrogen)	100
**CD19**	BUV395	1D3	#363-0193-82 (Invitrogen)	400
**CD11b**	BUV563	M1/70	#365-0112-82 (Invitrogen)	2000
**CD4**	BUV737	RM4-5	#367-0042-82 (Invitrogen)	400
**CD8a**	BUV805	53-6.7	#612898 (BD BioSciences)	400
**Ly6G**	BV480	1A8	#414-9668-82 (Invitrogen)	1000
**CD11c**	BV650	N418	#117339 (BioLegend)	400
**TCRgd**	BV711	GL3	#118149 (BioLegend)	400
**TCRb**	BV786	H57-597	#568222 (BD Biosciences)	400
**B220**	SparkBlue 574	RA3-6B2	#103289 (BioLegend)	800
**CD86**	RB613	GL-1	#759498 (BD Biosciences)	1600
**Ly6C**	PerCP-Cy5.5	HK1.4	#128011 (BioLegend)	1000
**CD62L**	RB780	MEL-14	#569210 (BD Biosciences)	1600
**CD161 (NK1.1)**	PE-Cy5	PK136	#108715 (BioLegend)	400
**CD44**	APC	IM7	#10-0441 (Tonbo)	1600
**CD45**	AlexaFluor 700	30-F11	#56-0451-82 (Invitrogen)	400
**CD3**	APC-Cy7	17A2	#25-0032 (Tonbo)	10000
**F4-80**	APC-Fire810	BM8.1	#123165 (Biolegend)	800
