# Supplementary material for: Immune remodeling via mitochondria-dependent STING activation enhances cabozantinib response in hepatocellular carcinoma
Source: J Exp Clin Cancer Res. 2026 Jan 9;45:42. doi: 10.1186/s13046-025-03632-z (PMC12882139; doi:10.1186/s13046-025-03632-z)
Supplement: Supplementary file 2 — Supplementary Material 2. [file 13046_2025_3632_MOESM2_ESM.pdf]

Figure 1B: Hep3B -GAPDH

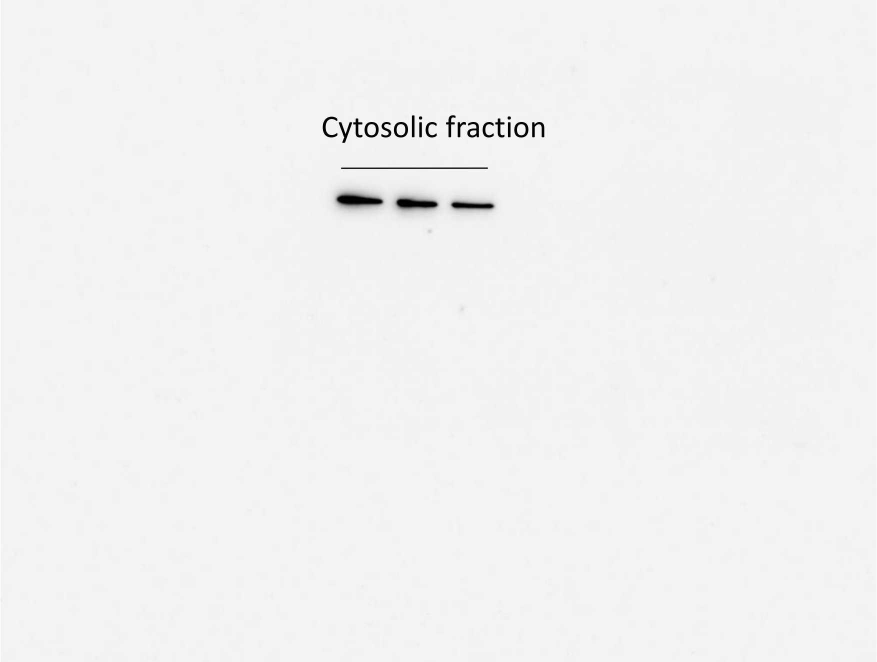

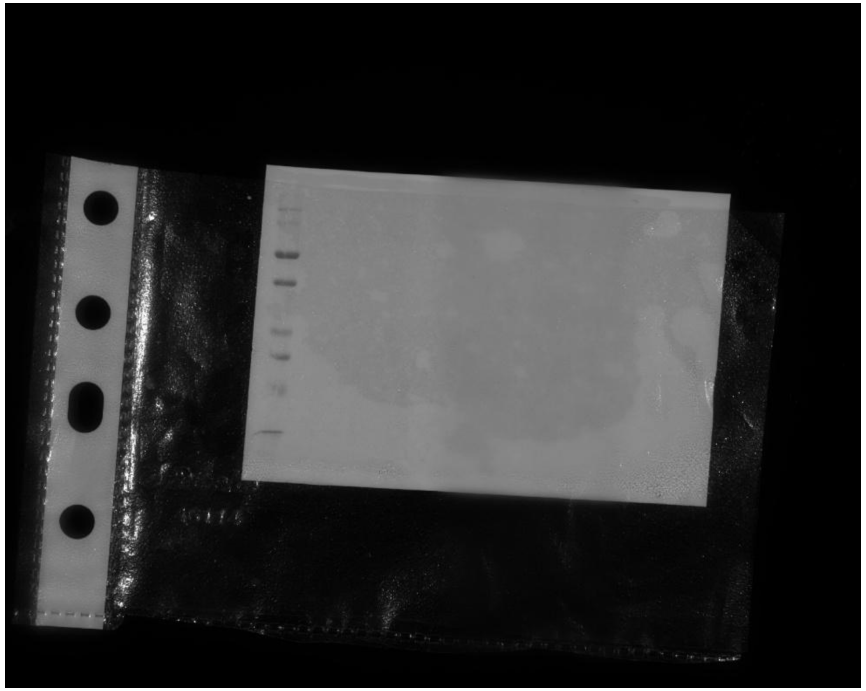

Figure 1B: Hep3B –TOM20

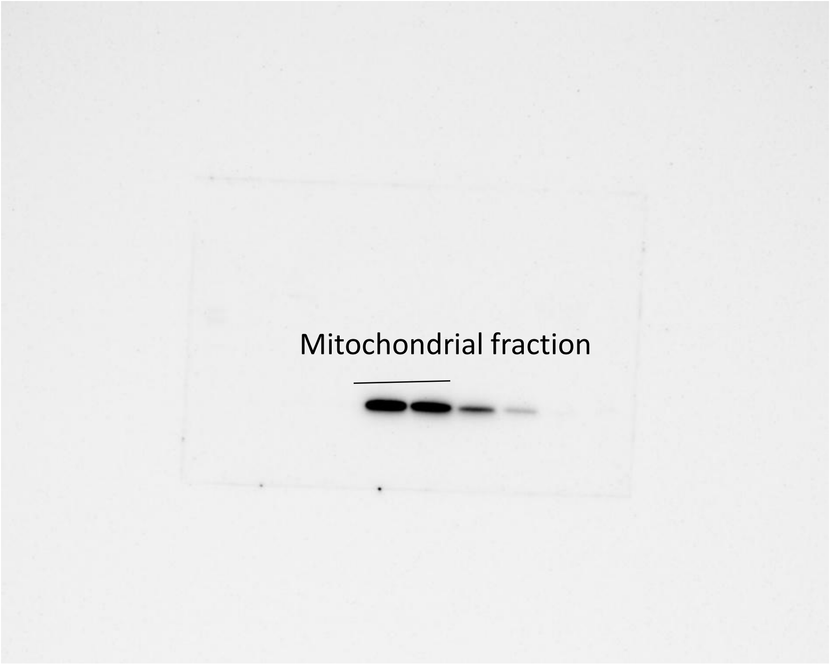

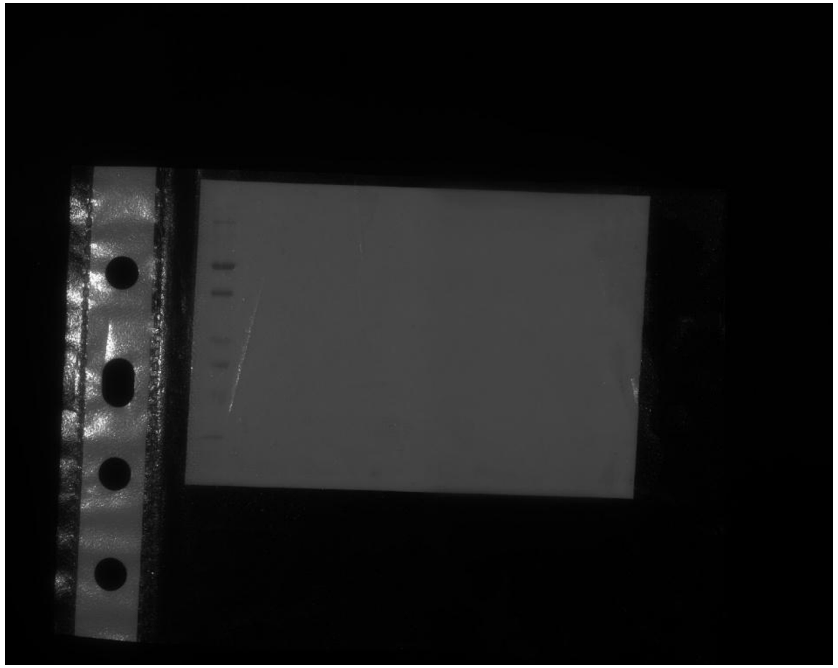

Figure 2A/C: WB Cabo Sting Hep3B –pTBK1

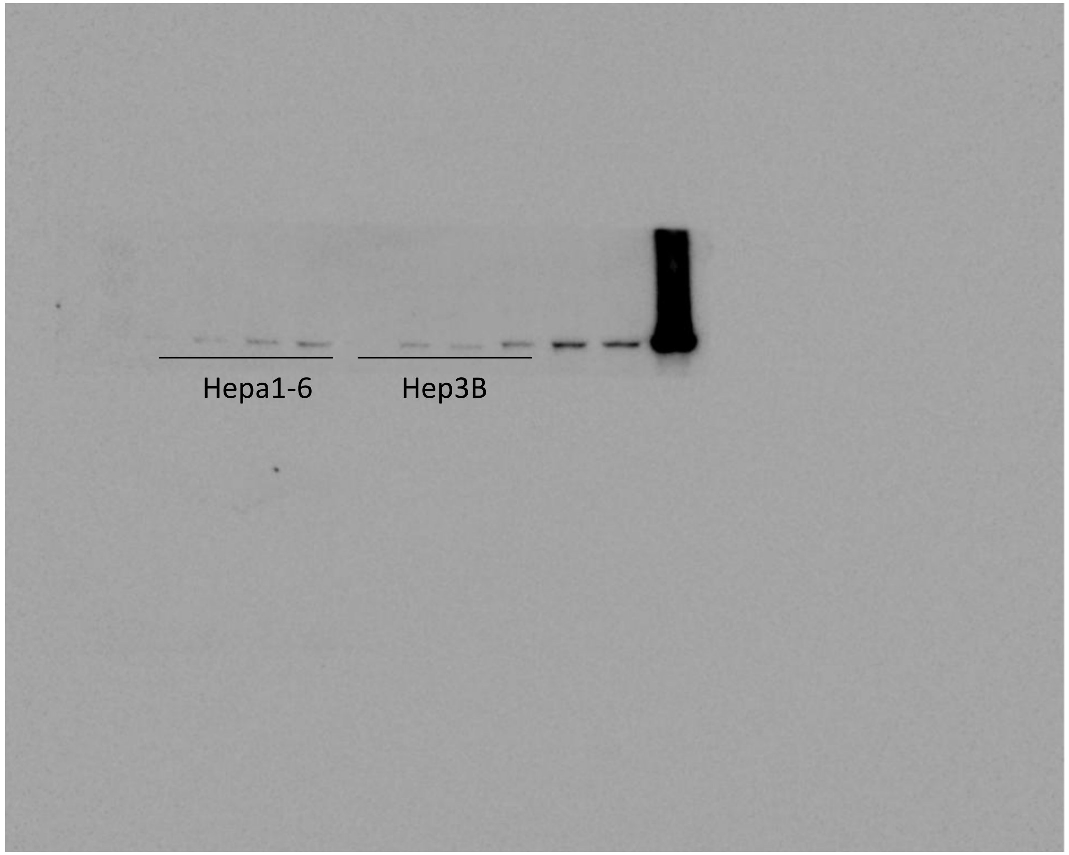

Gel order	Sample
1	**Marker**
2	Hepa1-6 control
3	Hepa1-6 cabo 50uM 2h
4	Hepa1-6 cabo 50uM 4h
5	Hepa1-6 cabo 50uM 8h
6	Hep3b Control
7	Hep3b Cabo 50uM 2h
8	Hep3b Cabo 50uM 4h
9	Hep3b Cabo 50uM 8h
10	thp1 control
11	thp1 Cabo20uM 2h
12	thp1 Vadimezan 100uM 2h

Figure 2A/C : WB Cabo Sting Hep3B –pTBK1

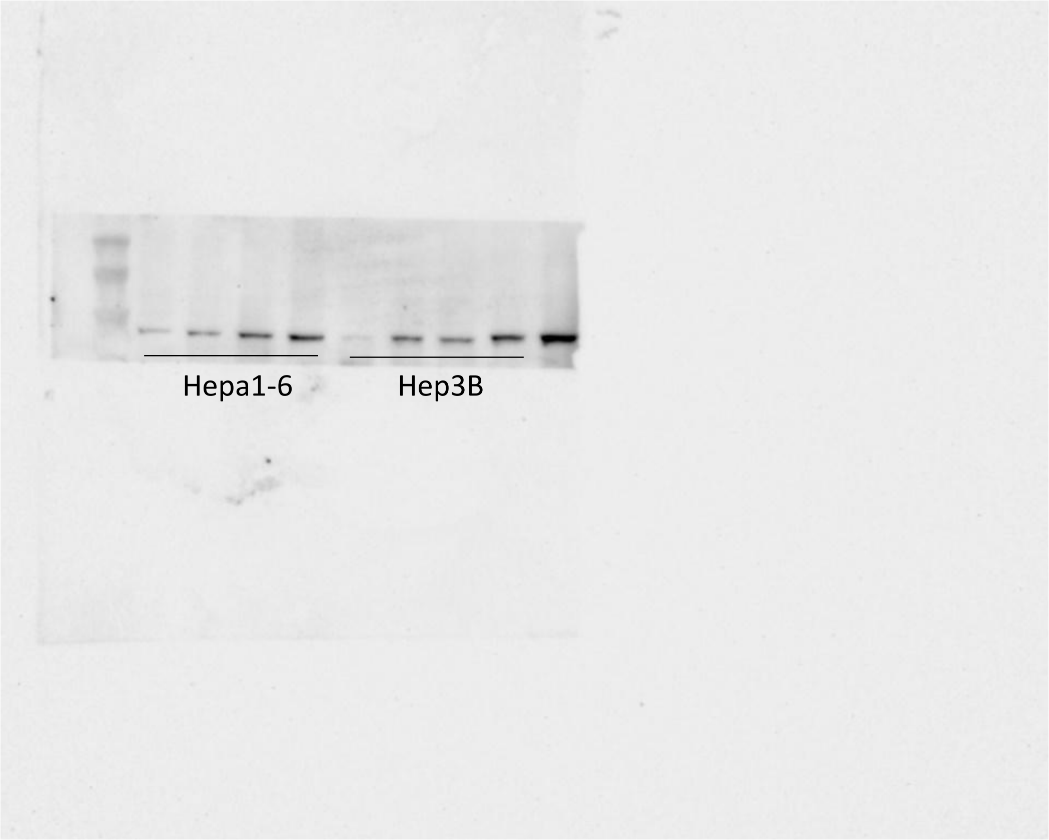

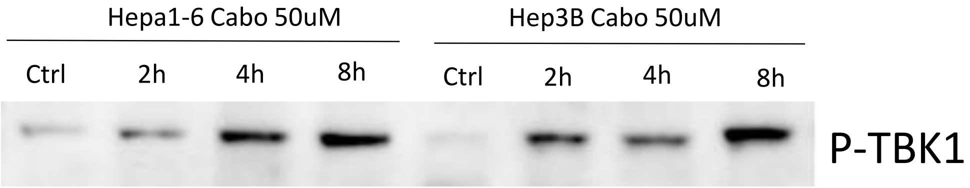

Gel order	Sample
1	**Marker**
2	Hepa1-6 control
3	Hepa1-6 cabo 50uM 2h
4	Hepa1-6 cabo 50uM 4h
5	Hepa1-6 cabo 50uM 8h
6	Hep3b Control
7	Hep3b Cabo 50uM 2h
8	Hep3b Cabo 50uM 4h
9	Hep3b Cabo 50uM 8h
10	thp1 control
11	thp1 Cabo20uM 2h
12	thp1 Vadimezan 100uM 2h

Figure 2A/C : WB Cabo Sting Hep3B -TBK

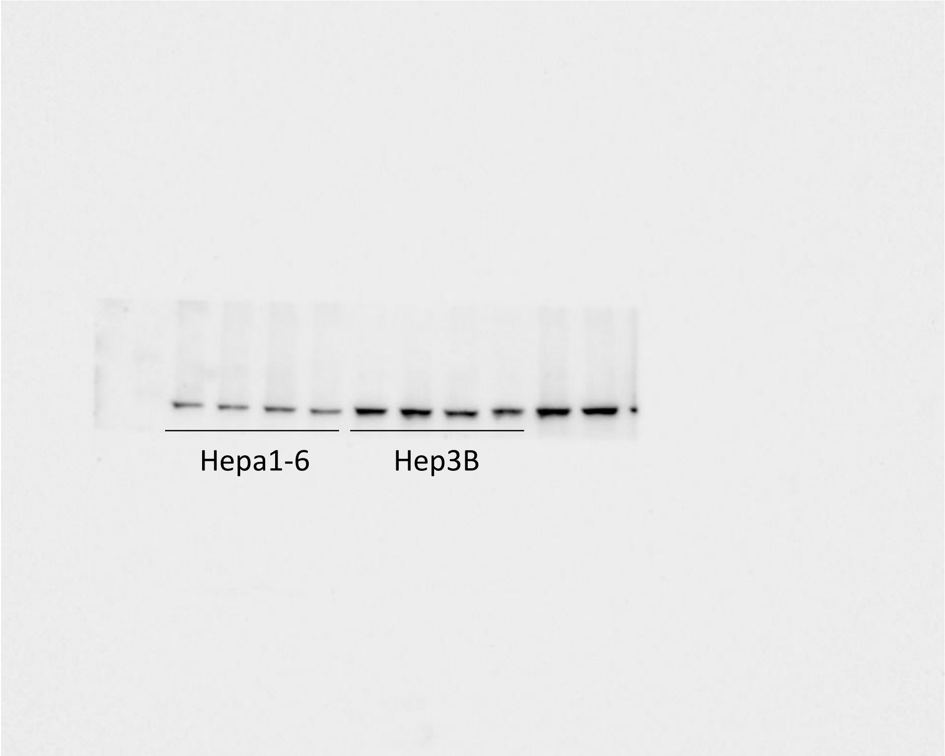

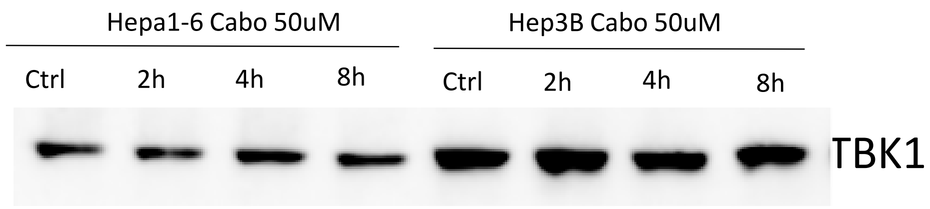

Gel order	Sample
1	**Marker**
2	Hepa1-6 control
3	Hepa1-6 cabo 50uM 2h
4	Hepa1-6 cabo 50uM 4h
5	Hepa1-6 cabo 50uM 8h
6	Hep3b Control
7	Hep3b Cabo 50uM 2h
8	Hep3b Cabo 50uM 4h
9	Hep3b Cabo 50uM 8h
10	thp1 control
11	thp1 Cabo20uM 2h
12	thp1 Vadimezan 100uM 2h

Figure 2A/C : WB Cabo Sting Hep3B –pIRF3

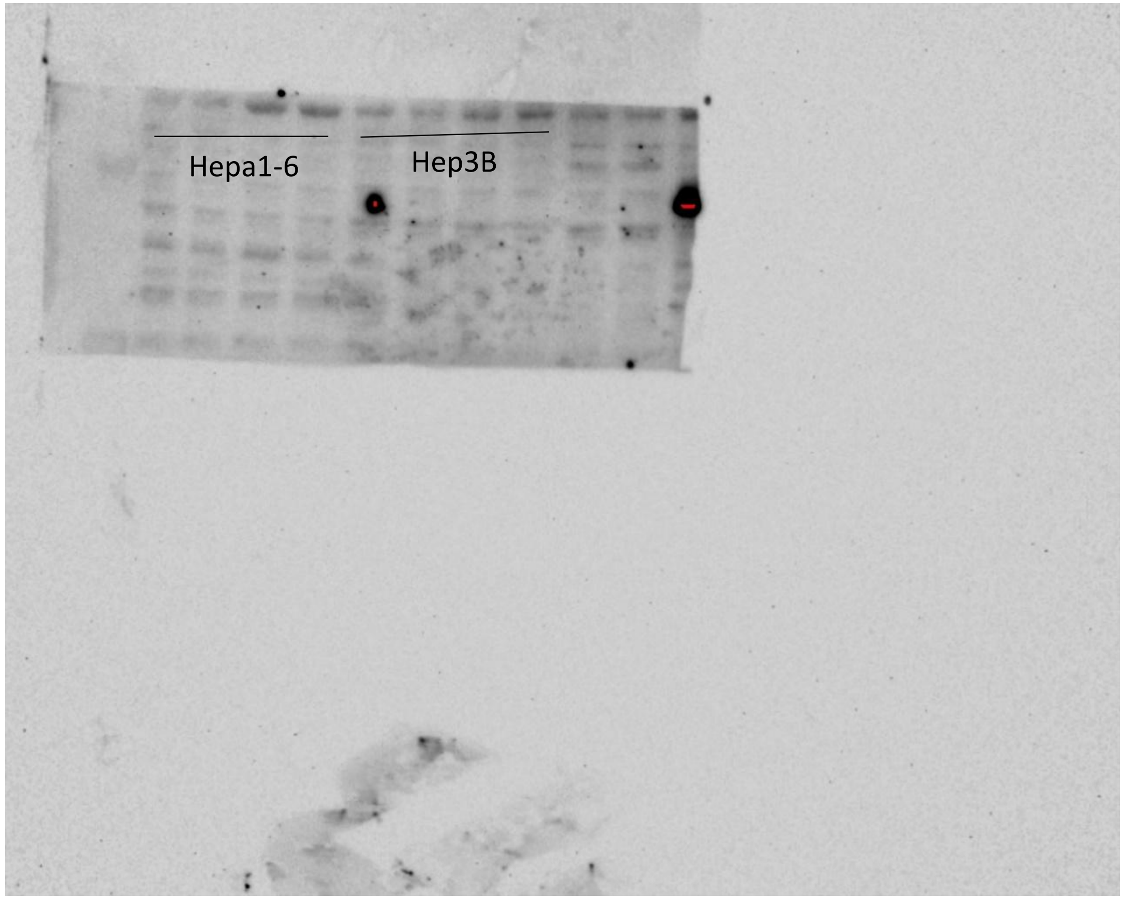

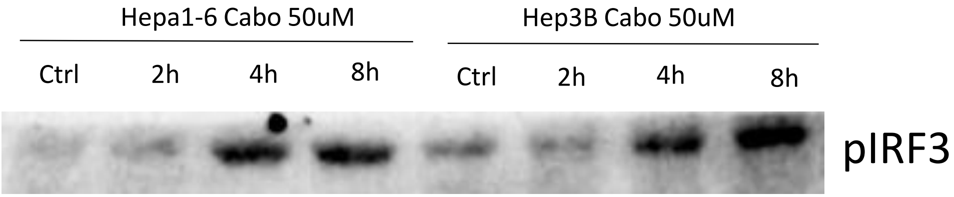

Gel order	Sample
1	**Marker**
2	Hepa1-6 control
3	Hepa1-6 cabo 50uM 2h
4	Hepa1-6 cabo 50uM 4h
5	Hepa1-6 cabo 50uM 8h
6	Hep3b Control
7	Hep3b Cabo 50uM 2h
8	Hep3b Cabo 50uM 4h
9	Hep3b Cabo 50uM 8h
10	thp1 control
11	thp1 Cabo20uM 2h
12	thp1 Vadimezan 100uM 2h

Figure 2A/C: WB Cabo Sting Hep3B –IRF3

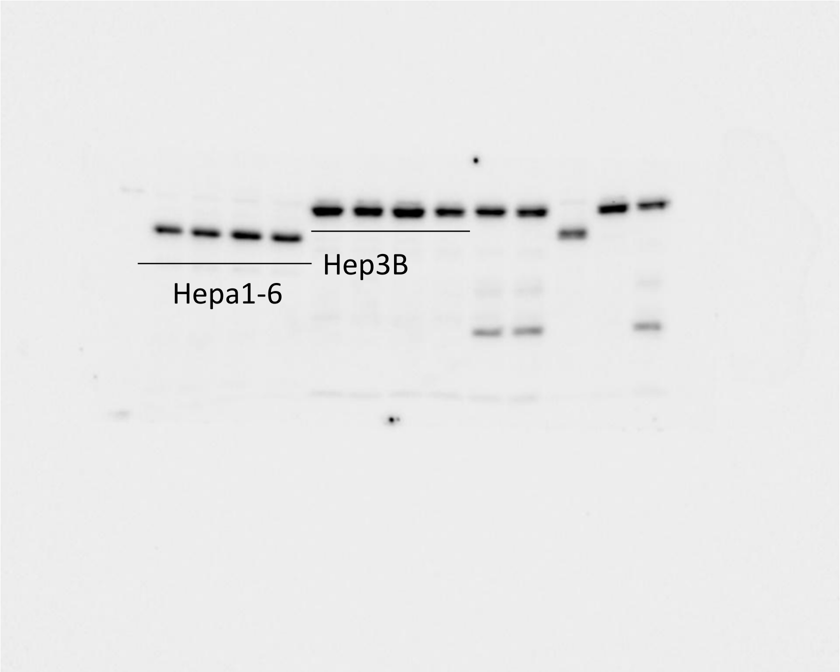

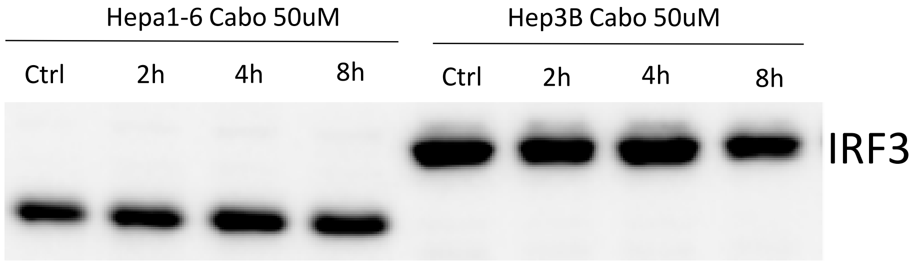

Gel order	Sample
1	**Marker**
2	Hepa1-6 control
3	Hepa1-6 cabo 50uM 2h
4	Hepa1-6 cabo 50uM 4h
5	Hepa1-6 cabo 50uM 8h
6	Hep3b Control
7	Hep3b Cabo 50uM 2h
8	Hep3b Cabo 50uM 4h
9	Hep3b Cabo 50uM 8h
10	thp1 control
11	thp1 Cabo20uM 2h
12	thp1 Vadimezan 100uM 2h

Figure 2A/C: WB Cabo Sting Hep3B –ACTIN

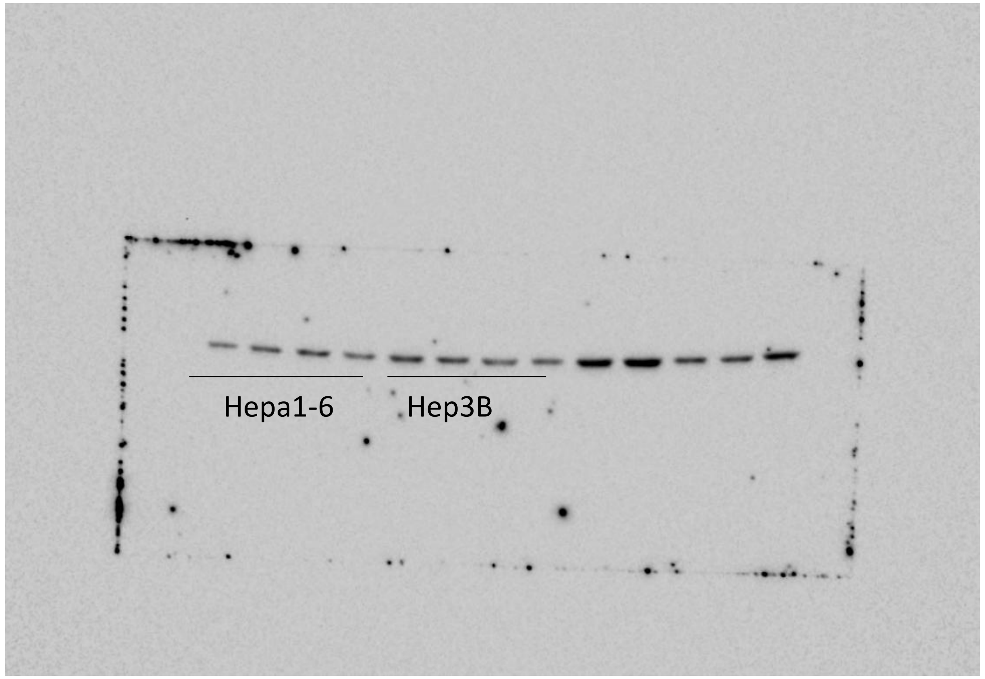

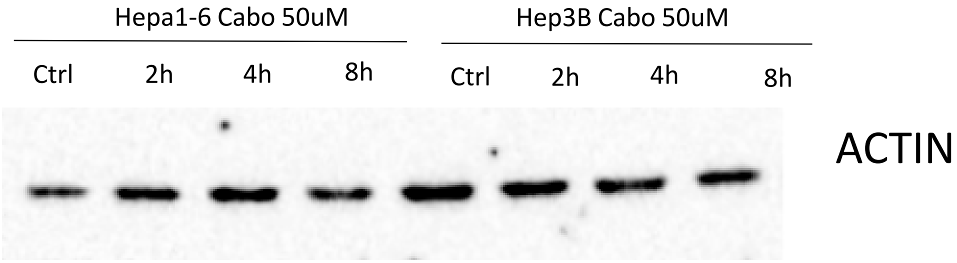

Gel order	Sample
1	**Marker**
2	Hepa1-6 control
3	Hepa1-6 cabo 50uM 2h
4	Hepa1-6 cabo 50uM 4h
5	Hepa1-6 cabo 50uM 8h
6	Hep3b Control
7	Hep3b Cabo 50uM 2h
8	Hep3b Cabo 50uM 4h
9	Hep3b Cabo 50uM 8h
10	thp1 control
11	thp1 Cabo20uM 2h
12	thp1 Vadimezan 100uM 2h

Figure 2F: WB Cabo Sting Hepa1-6-pTBK1

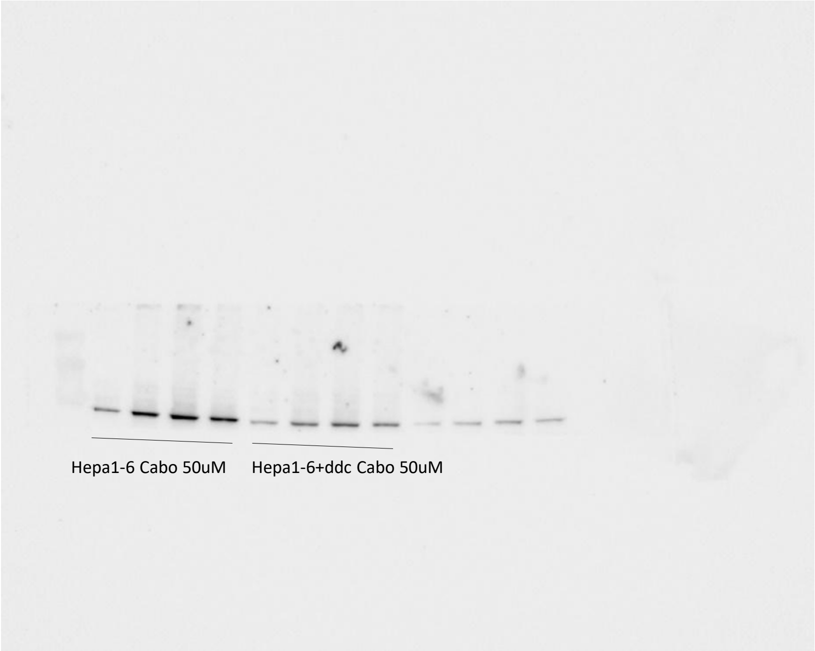

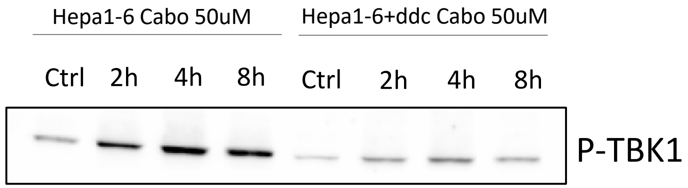

Gel order	Sample
1	**Marker**
2	Hepa1-6 control
3	Hepa1-6 cabo 50uM 2h
4	Hepa1-6 cabo 50uM 4h
5	Hepa1-6 cabo 50uM 8h
6	Hep3b Control
7	Hep3b Cabo 50uM 2h
8	Hep3b Cabo 50uM 4h
9	Hep3b Cabo 50uM 8h
10	thp1 control
11	thp1 Cabo20uM 2h
12	thp1 Vadimezan 100uM 2h

Figure 2F: WB Cabo Sting Hepa1-6-TBK1

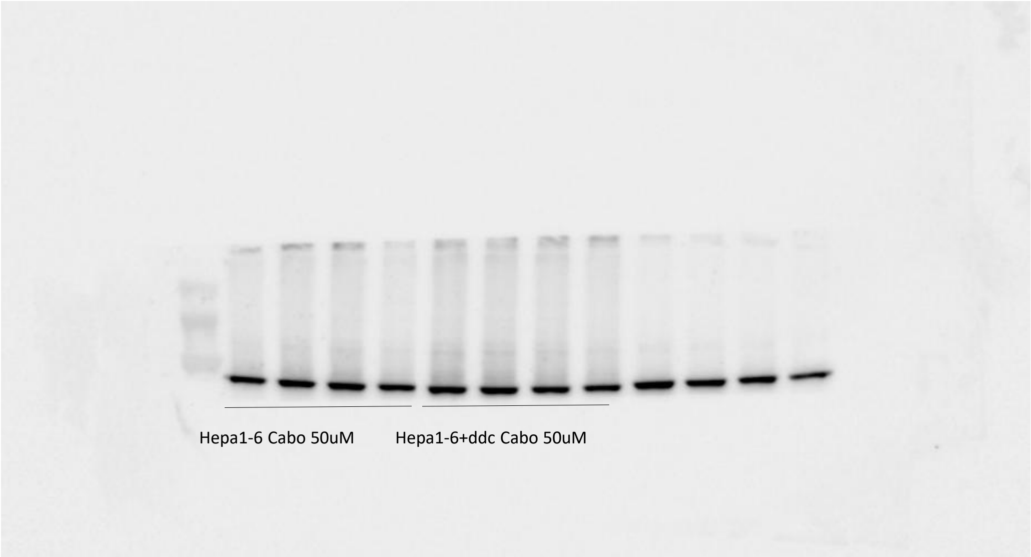

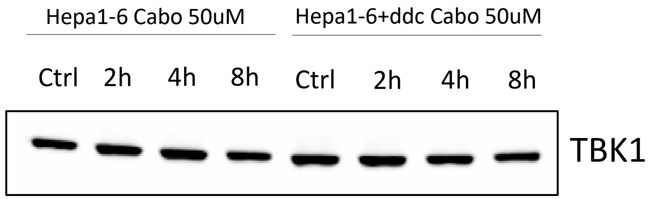

Gel order	Sample
1	**Marker**
2	Hepa1-6 control
3	Hepa1-6 cabo 50uM 2h
4	Hepa1-6 cabo 50uM 4h
5	Hepa1-6 cabo 50uM 8h
6	Hep3b Control
7	Hep3b Cabo 50uM 2h
8	Hep3b Cabo 50uM 4h
9	Hep3b Cabo 50uM 8h
10	thp1 control
11	thp1 Cabo20uM 2h
12	thp1 Vadimezan 100uM 2h

Figure 2F: WB Cabo Sting Hepa1-6-pIRF3

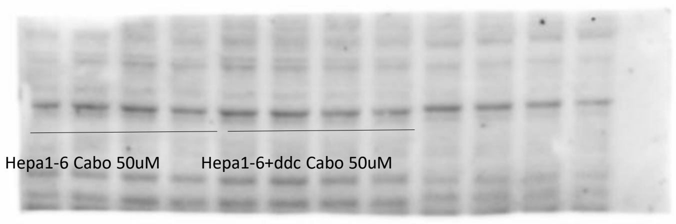

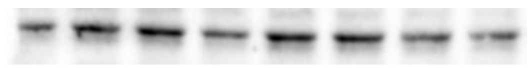

Gel order	Sample
1	**Marker**
2	Hepa1-6 control
3	Hepa1-6 cabo 50uM 2h
4	Hepa1-6 cabo 50uM 4h
5	Hepa1-6 cabo 50uM 8h
6	Hep3b Control
7	Hep3b Cabo 50uM 2h
8	Hep3b Cabo 50uM 4h
9	Hep3b Cabo 50uM 8h
10	thp1 control
11	thp1 Cabo20uM 2h
12	thp1 Vadimezan 100uM 2h

Figure 2F: WB Cabo Sting Hepa1-6-IRF3

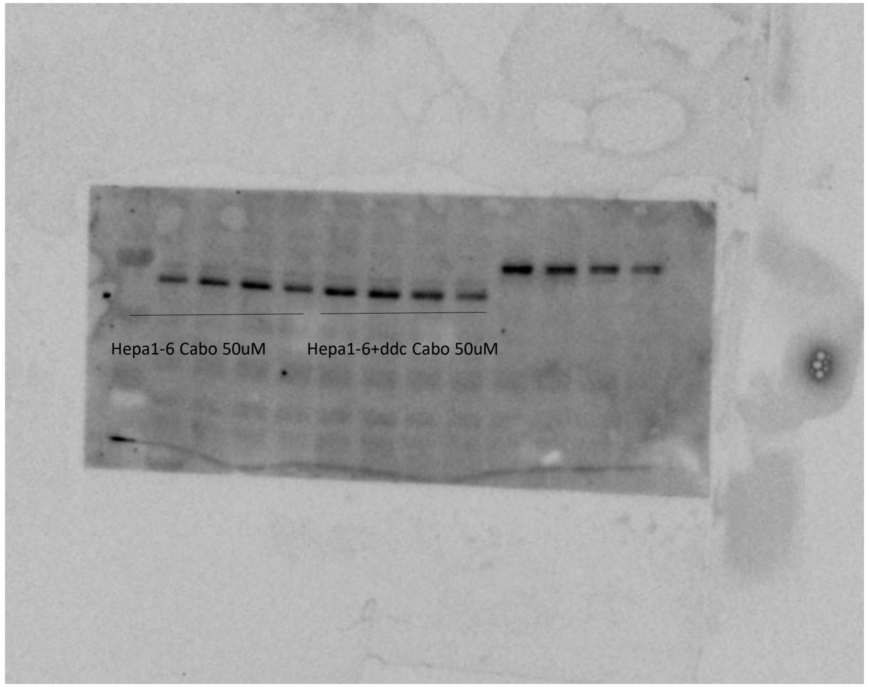

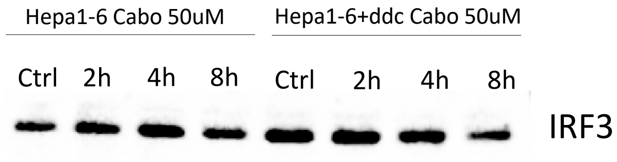

Gel order	Sample
1	**Marker**
2	Hepa1-6 control
3	Hepa1-6 cabo 50uM 2h
4	Hepa1-6 cabo 50uM 4h
5	Hepa1-6 cabo 50uM 8h
6	Hep3b Control
7	Hep3b Cabo 50uM 2h
8	Hep3b Cabo 50uM 4h
9	Hep3b Cabo 50uM 8h
10	thp1 control
11	thp1 Cabo20uM 2h
12	thp1 Vadimezan 100uM 2h

Figure 2F: WB Cabo Sting Hepa1-6-pSTING

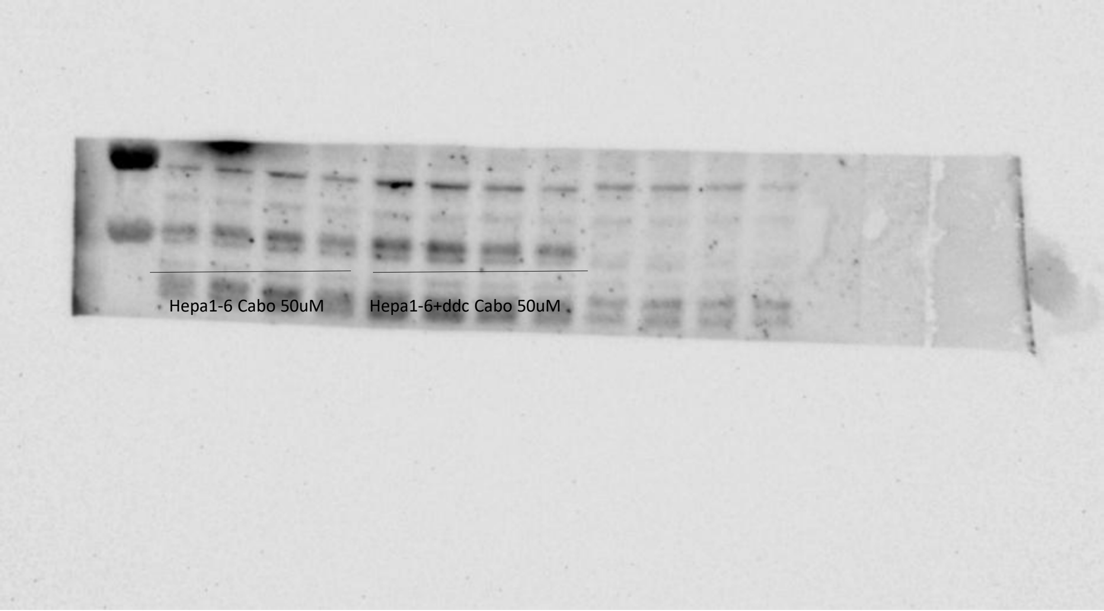

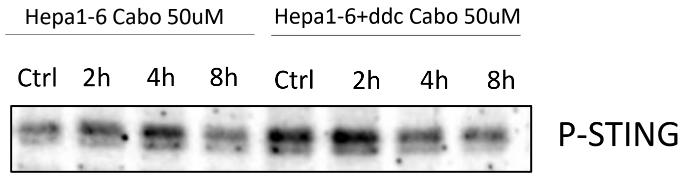

Gel order	Sample
1	**Marker**
2	Hepa1-6 control
3	Hepa1-6 cabo 50uM 2h
4	Hepa1-6 cabo 50uM 4h
5	Hepa1-6 cabo 50uM 8h
6	Hepa1-6 +ddc control
7	Hepa1-6 +ddc cabo 50uM 2h
8	Hepa1-6 +ddc cabo 50uM 4h
9	Hepa1-6 +ddc cabo 50uM 8h
10	Hep3B control
11	Hep3B cabo 50uM 2h
12	Hep3B cabo 50uM 4h
13	Hep3B cabo 50uM 8h

Figure 2F: WB Cabo Sting Hepa1-6-STING

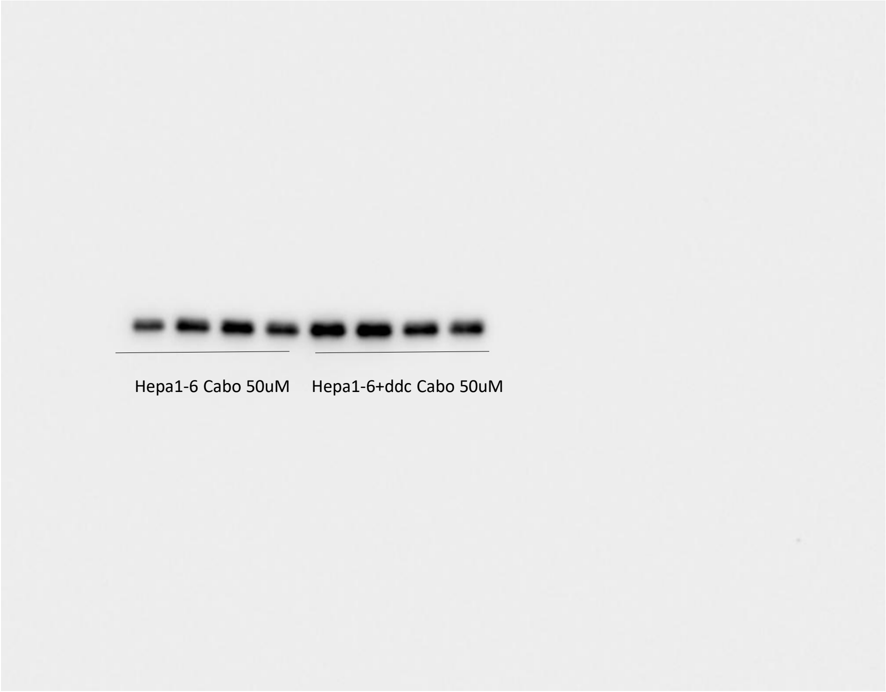

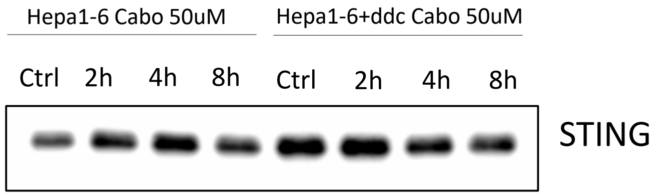

Gel order	Sample
1	**Marker**
2	Hepa1-6 control
3	Hepa1-6 cabo 50uM 2h
4	Hepa1-6 cabo 50uM 4h
5	Hepa1-6 cabo 50uM 8h
6	Hepa1-6 +ddc control
7	Hepa1-6 +ddc cabo 50uM 2h
8	Hepa1-6 +ddc cabo 50uM 4h
9	Hepa1-6 +ddc cabo 50uM 8h
10	Hep3B control
11	Hep3B cabo 50uM 2h
12	Hep3B cabo 50uM 4h
13	Hep3B cabo 50uM 8h

Figure 2F: WB Cabo Sting Hepa1-6-ACTIN

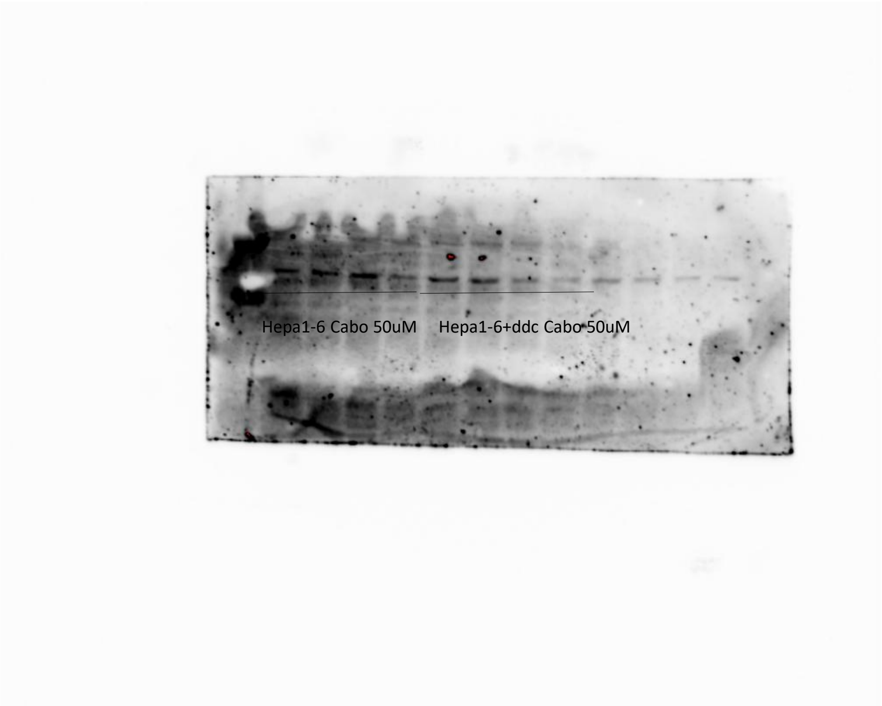

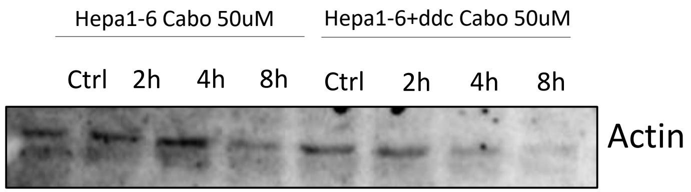

Gel order	Sample
1	**Marker**
2	Hepa1-6 control
3	Hepa1-6 cabo 50uM 2h
4	Hepa1-6 cabo 50uM 4h
5	Hepa1-6 cabo 50uM 8h
6	Hepa1-6 +ddc control
7	Hepa1-6 +ddc cabo 50uM 2h
8	Hepa1-6 +ddc cabo 50uM 4h
9	Hepa1-6 +ddc cabo 50uM 8h
10	Hep3B control
11	Hep3B cabo 50uM 2h
12	Hep3B cabo 50uM 4h
13	Hep3B cabo 50uM 8h

Figure 2B: WB Nuclear Fraction- p65

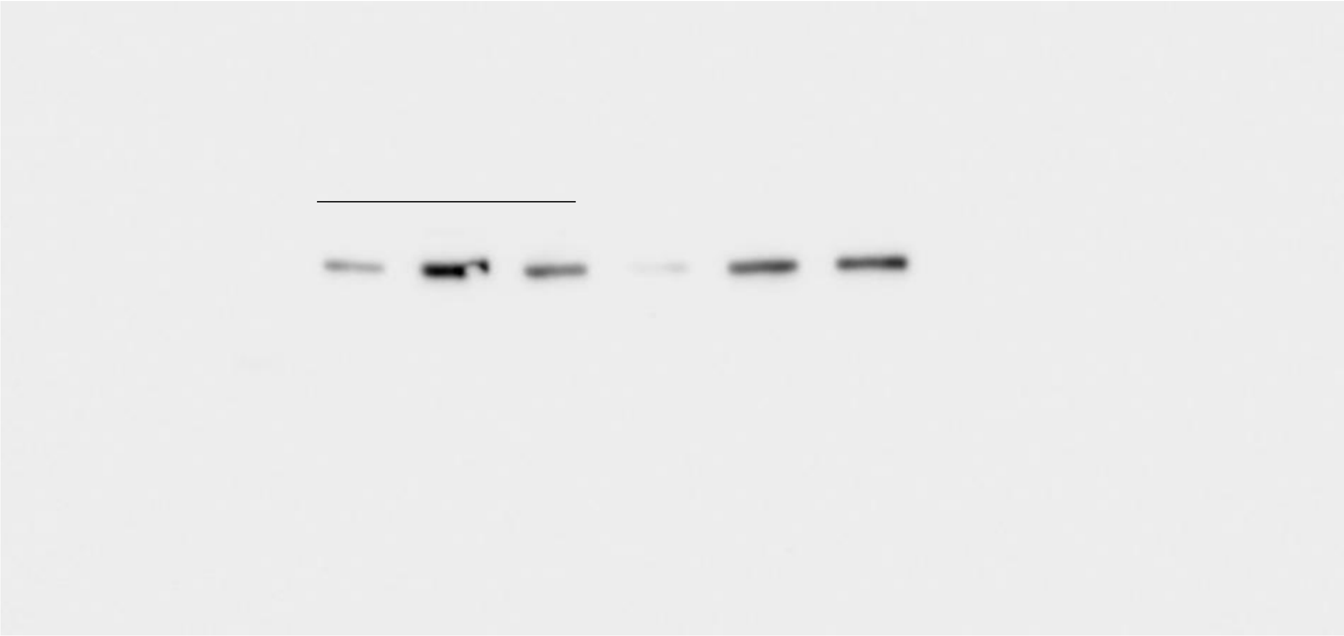

Gel order	Sample
1	Marker
2	1 Hep3B Ctrl
3	3 Hep3B Cabo 30 min
4	4 Hep3B Cabo 1h

Figure 2B: WB Nuclear Fraction- IRF3

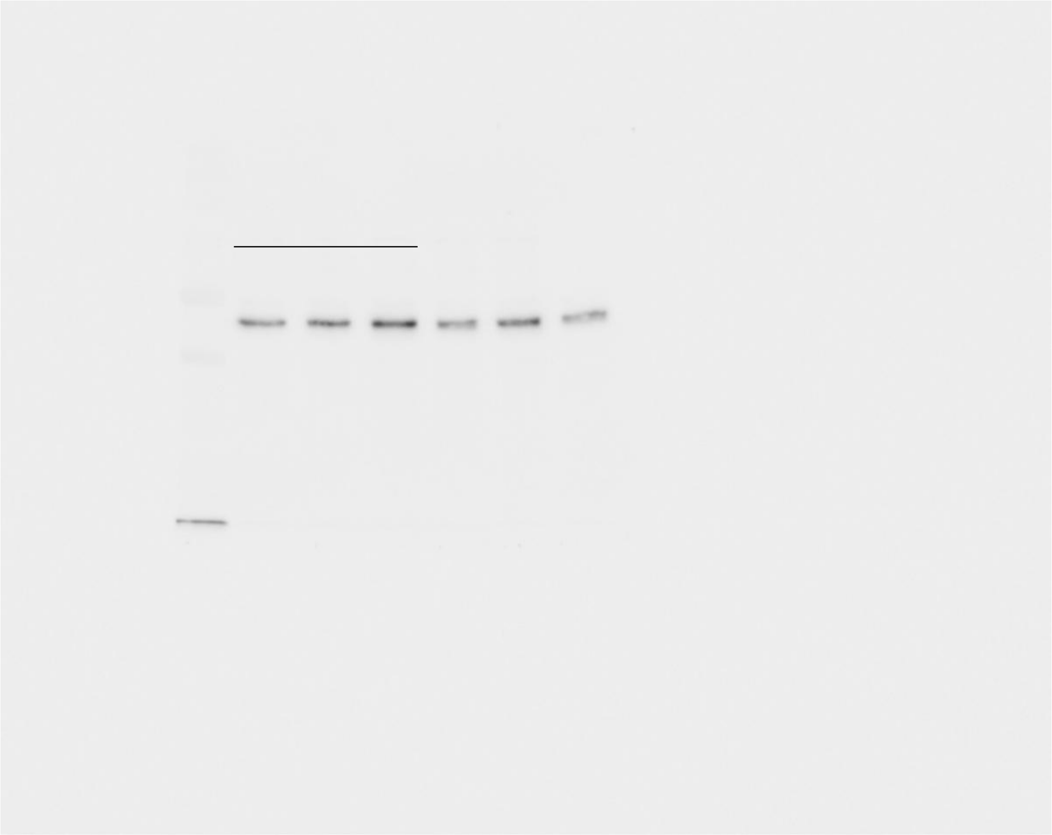

Gel order	Sample
1	Marker
2	1 Hep3B Ctrl
3	3 Hep3B Cabo 30 min
4	4 Hep3B Cabo 1h

Figure 3: WB Cabo Sting KO Hepa1-6 –pTBK1

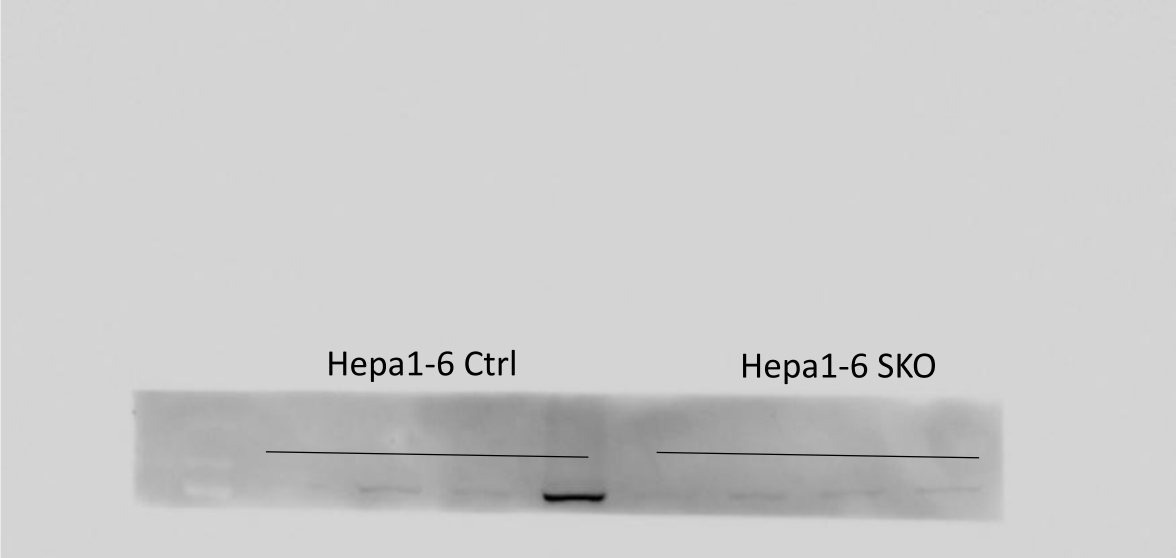

Gel order	Sample
1	Marker
2	1 Hepa1-6 CNEG1 Ctrl
3	2 Hepa1-6 CNEG1 Cabo 50uM 4h
4	3 Hepa1-6 CNEG1 Cabo 50uM 8h
5	4 Hepa1-6 CNEG1 Vad100uM 2h
6	1Hepa1-6 STING KO 1 Ctrl
7	2 Hepa1-6 STING KO 1 Cabo 50uM 4h
8	3 Hepa1-6 STING KO 1 Cabo 50uM 8h
9	4 Hepa1-6 STING KO 1 Vad100uM 2h

Figure 3: WB Cabo Sting KO Hepa1-6 –TBK1

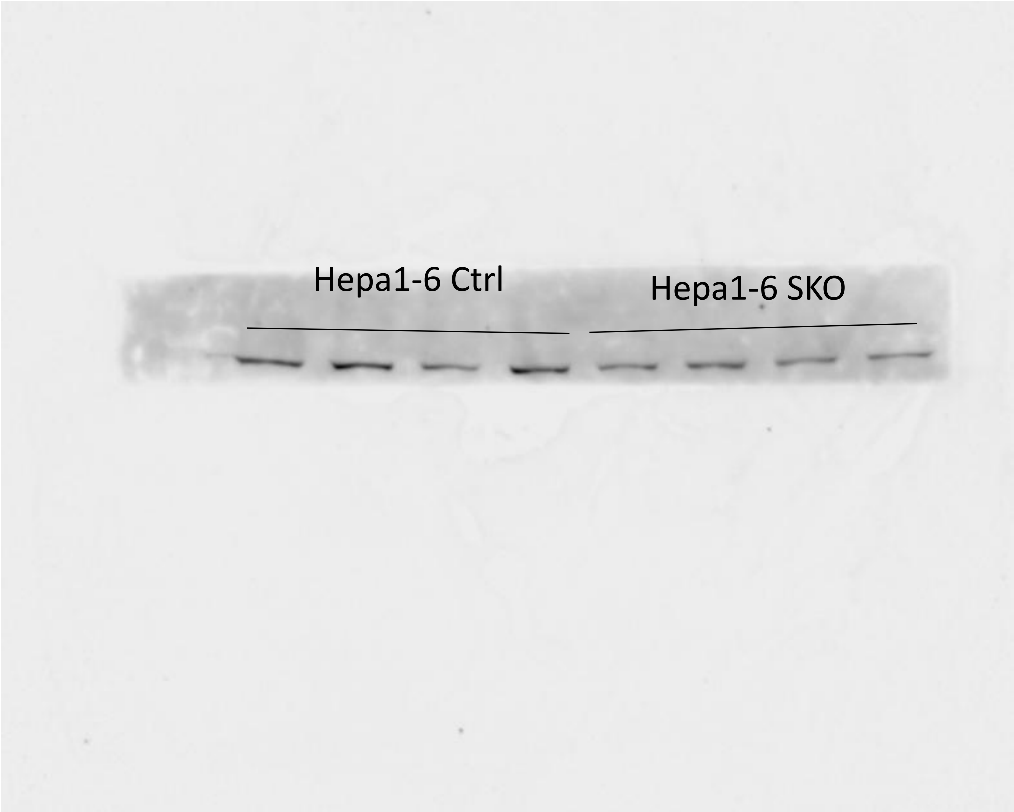

Gel order	Sample
1	Marker
2	1 Hepa1-6 CNEG1 Ctrl
3	2 Hepa1-6 CNEG1 Cabo 50uM 4h
4	3 Hepa1-6 CNEG1 Cabo 50uM 8h
5	4 Hepa1-6 CNEG1 Vad100uM 2h
6	1Hepa1-6 STING KO 1 Ctrl
7	2 Hepa1-6 STING KO 1 Cabo 50uM 4h
8	3 Hepa1-6 STING KO 1 Cabo 50uM 8h
9	4 Hepa1-6 STING KO 1 Vad100uM 2h

Figure 3: WB Cabo Sting KO Hepa1-6 –STING

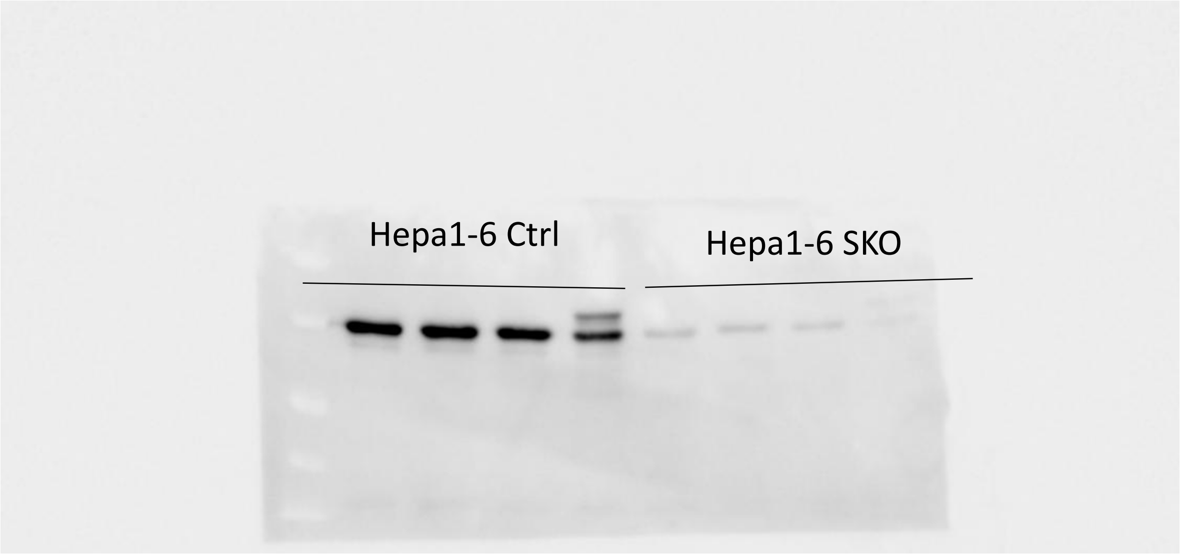

Gel order	Sample
1	Marker
2	1 Hepa1-6 CNEG1 Ctrl
3	2 Hepa1-6 CNEG1 Cabo 50uM 4h
4	3 Hepa1-6 CNEG1 Cabo 50uM 8h
5	4 Hepa1-6 CNEG1 Vad100uM 2h
6	1Hepa1-6 STING KO 1 Ctrl
7	2 Hepa1-6 STING KO 1 Cabo 50uM 4h
8	3 Hepa1-6 STING KO 1 Cabo 50uM 8h
9	4 Hepa1-6 STING KO 1 Vad100uM 2h

Figure 4A: WB HMGB1 MEDIA

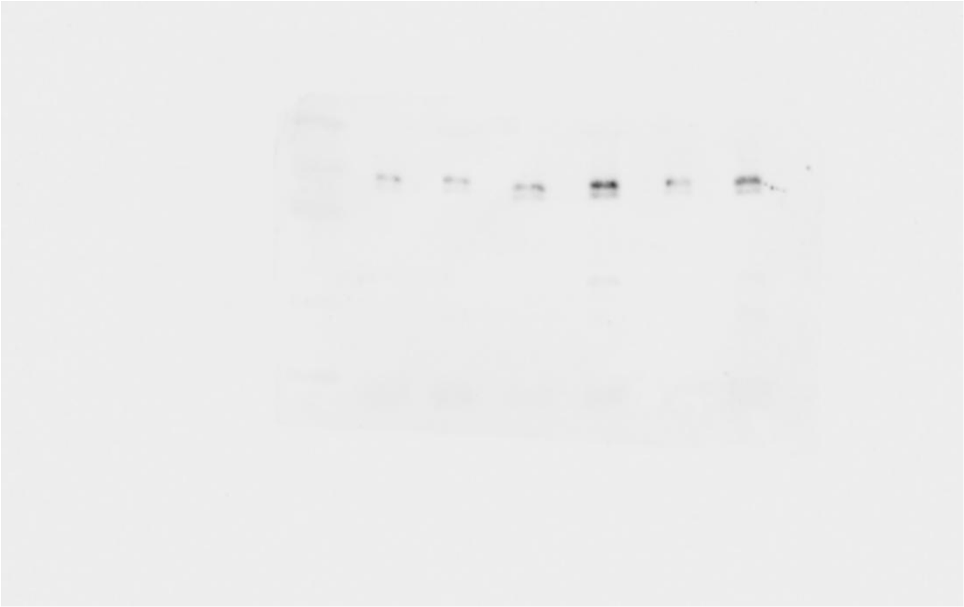

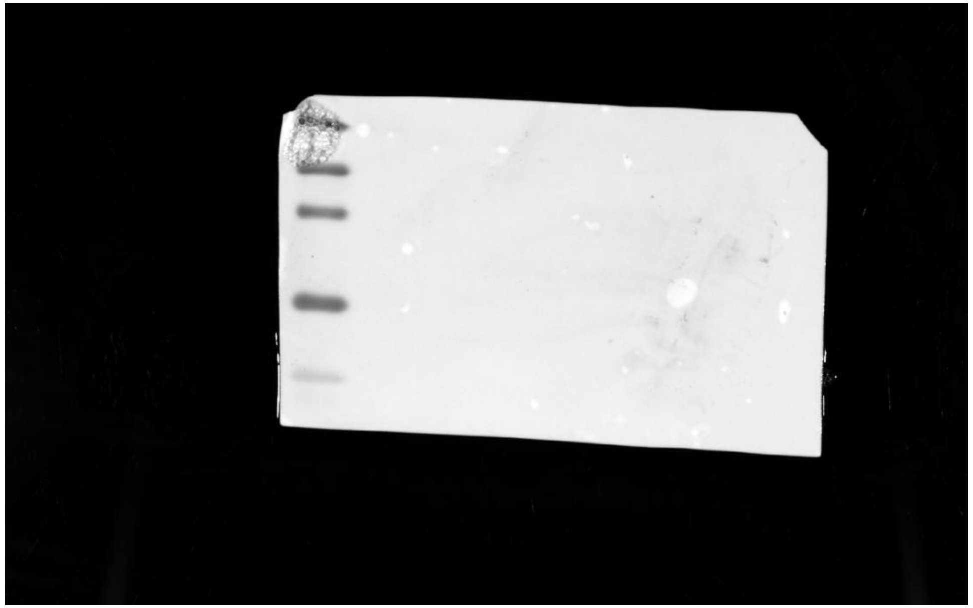

Orden gel	Muestra fraccion celular
1	Marker
2	1 Hep3B Ctrl
3	3 Hep3B Cabo20uM 4h
4	5 Hep3B Cabo20uM 8h
5	7 Hep3B Cabo20uM ON
6	9 Hep3B Cabo10uM 8h
7	11 Hep3B Cabo10uM ON

Figure 4A: WB HMGB1 TOTAL CELL EXTRACT

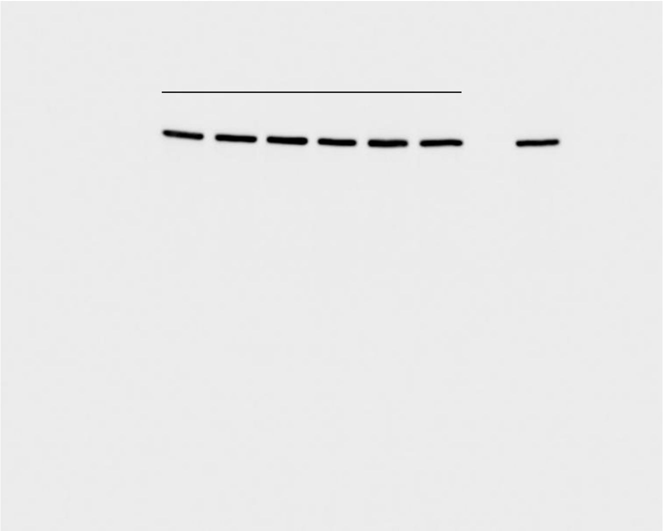

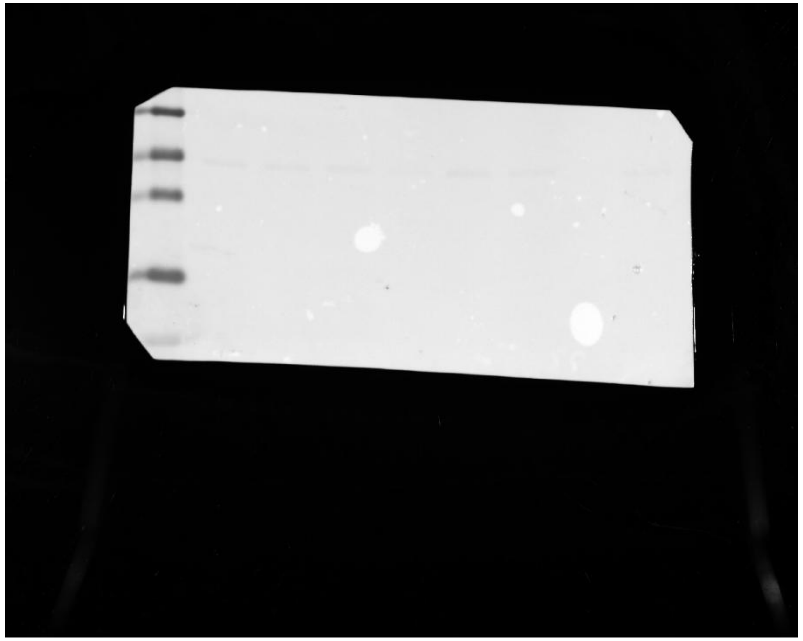

Orden gel	Muestra fraccion celular
1	**Marker**
2	**1 Hep3B Ctrl**
3	**3 Hep3B Cabo20uM 4h**
4	**5 Hep3B Cabo20uM 8h**
5	**7 Hep3B Cabo20uM ON**
6	**9 Hep3B Cabo10uM 8h**
7	**11 Hep3B Cabo10uM ON**

Figure 4A: WB ACTIN TOTAL CELL EXTRACT

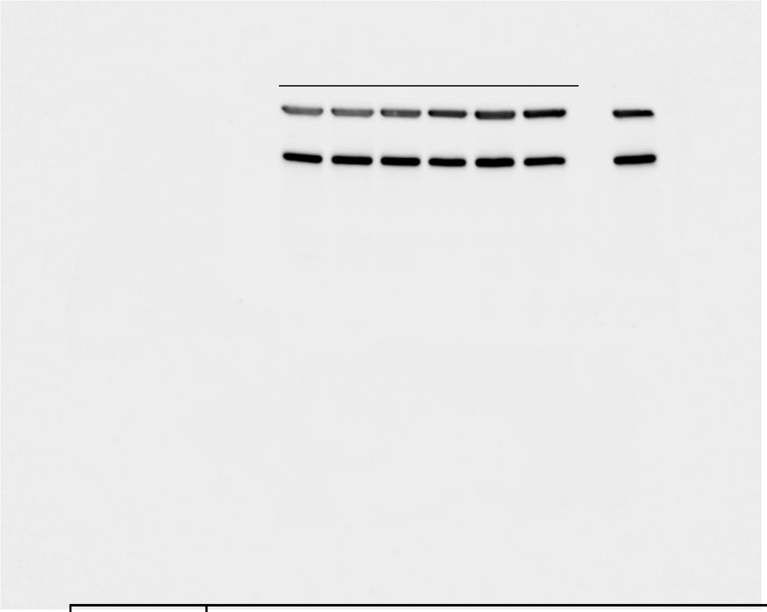

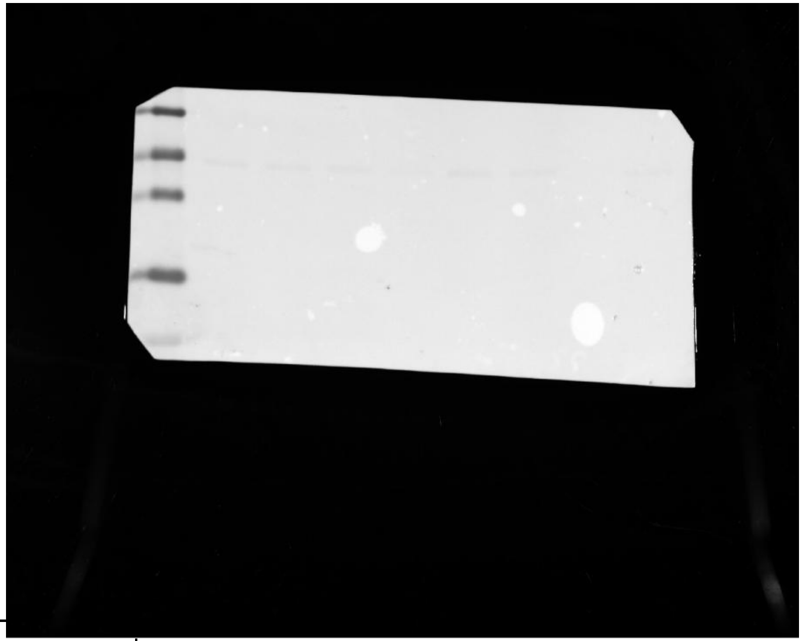

Orden gel	Muestra fraccion celular
1	Marker
2	1 Hep3B Ctrl
3	3 Hep3B Cabo20uM 4h
4	5 Hep3B Cabo20uM 8h
5	7 Hep3B Cabo20uM ON
6	9 Hep3B Cabo10uM 8h
7	11 Hep3B Cabo10uM ON

Figure 4D/F: WB HEP3B LENVATINIB SORAFENIB–pTBK1

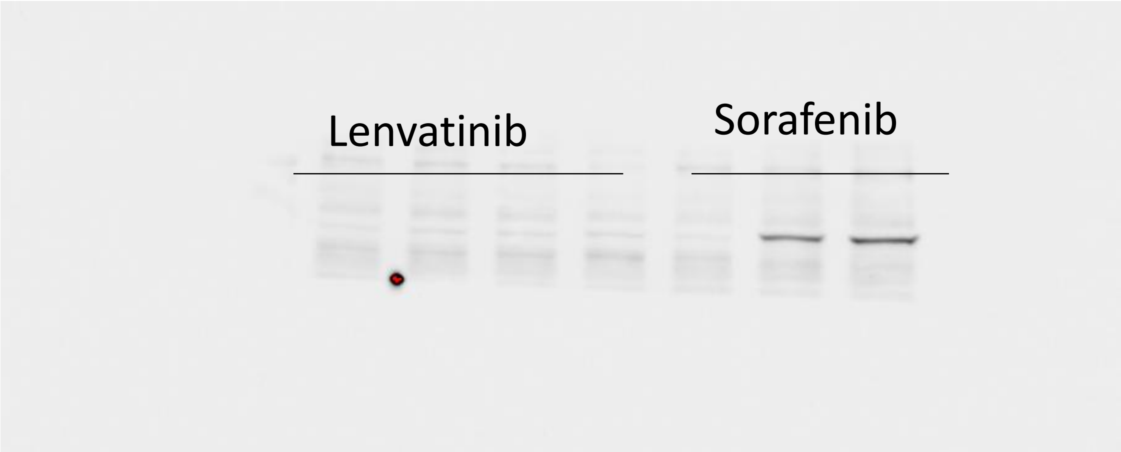

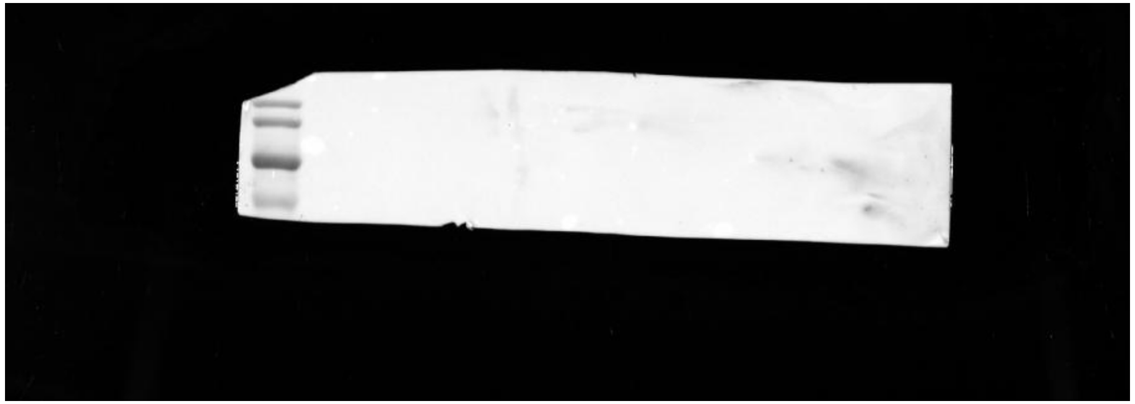

Gel order	Sample
1	Marker
2	1 Hep3B Ctrl
3	3 Hep3B Lenva 20uM 4h
4	5 Hep3B Lenva 20uM 8h
5	6 Hep3B Lenva 20uM on
6	1 Hep3B Ctrl
7	3 Hep3B Sor20uM 4h
	3 Hep3B Sor20uM 8h

Figure 4D/F: WB HEP3B LENVATINIB SORAFENIB–TBK1

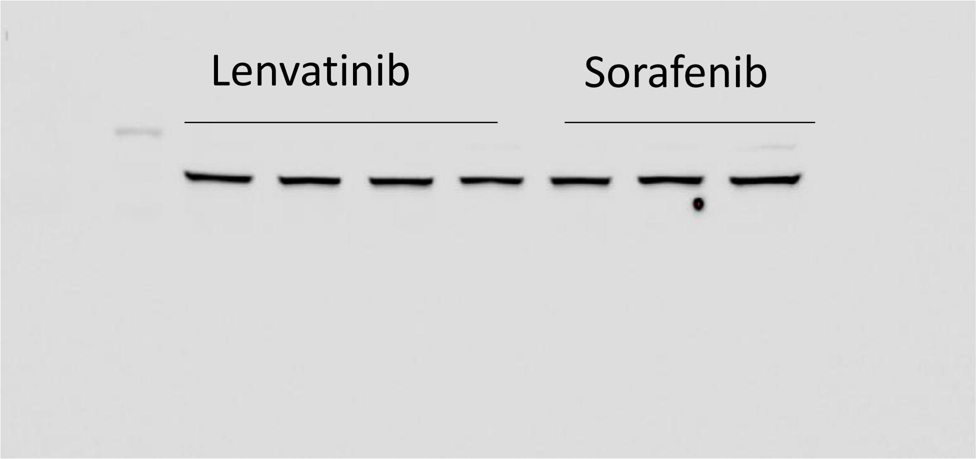

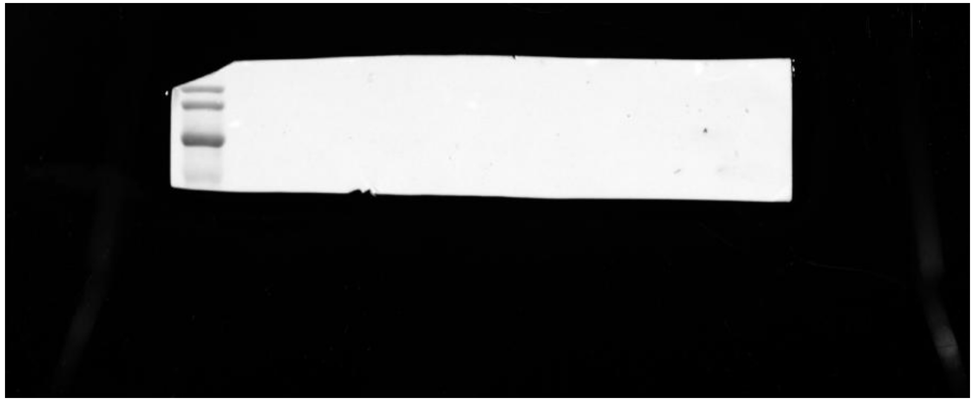

Gel order	Sample
1	Marker
2	1 Hep3B Ctrl
3	3 Hep3B Lenva 20uM 4h
4	5 Hep3B Lenva 20uM 8h
5	6 Hep3B Lenva 20uM on
6	1 Hep3B Ctrl
7	3 Hep3B Sor20uM 4h
	3 Hep3B Sor20uM 8h

Figure 4D/F: WB HEP3B LENVATINIB SORAFENIB –Actin

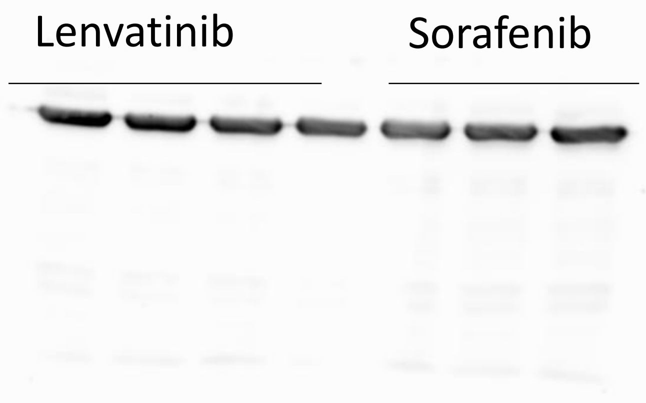

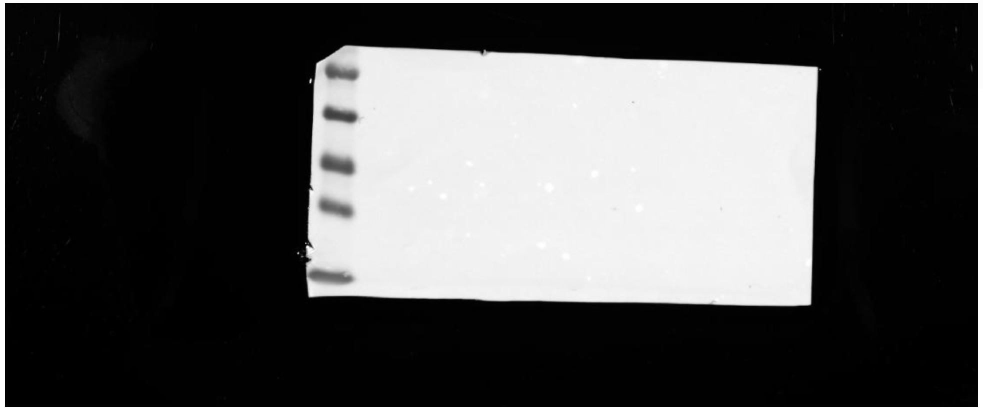

Gel order	Sample
1	Marker
2	1 Hep3B Ctrl
3	3 Hep3B Lenva 20uM 4h
4	5 Hep3B Lenva 20uM 8h
5	6 Hep3B Lenva 20uM on
6	1 Hep3B Ctrl
7	3 Hep3B Sor20uM 4h
	3 Hep3B Sor20uM 8h
