# Supplementary material for: Immune remodeling via mitochondria-dependent STING activation enhances cabozantinib response in hepatocellular carcinoma
Source: J Exp Clin Cancer Res. 2026 Jan 9;45:42. doi: 10.1186/s13046-025-03632-z (PMC12882139; doi:10.1186/s13046-025-03632-z)
Supplement: Supplementary file 3 — Supplementary Material 3. [file 13046_2025_3632_MOESM3_ESM.pdf]

WB Suplemental figures

- Figure 1.A BCLC5 Cells TBK1

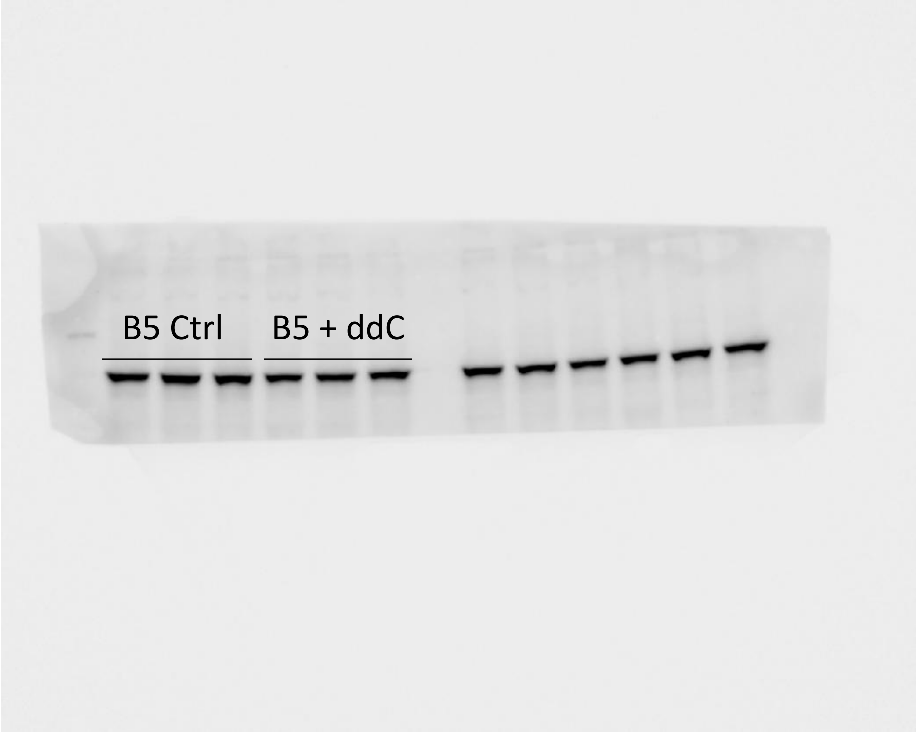

Gel order	Sample
1	**Marker**
2	Bclc5 control
3	Bclc5 Cabo 20uM 4h
4	Bclc5 Cabo 20uM 8h
5	Bclc5 control +ddc
6	Bclc5 +ddc Cabo 20uM 4h
7	Bclc5 +ddc Cabo 20uM 8h

WB Suplemental figures

- Figure 1.A BCLC5 Cells pTBK1

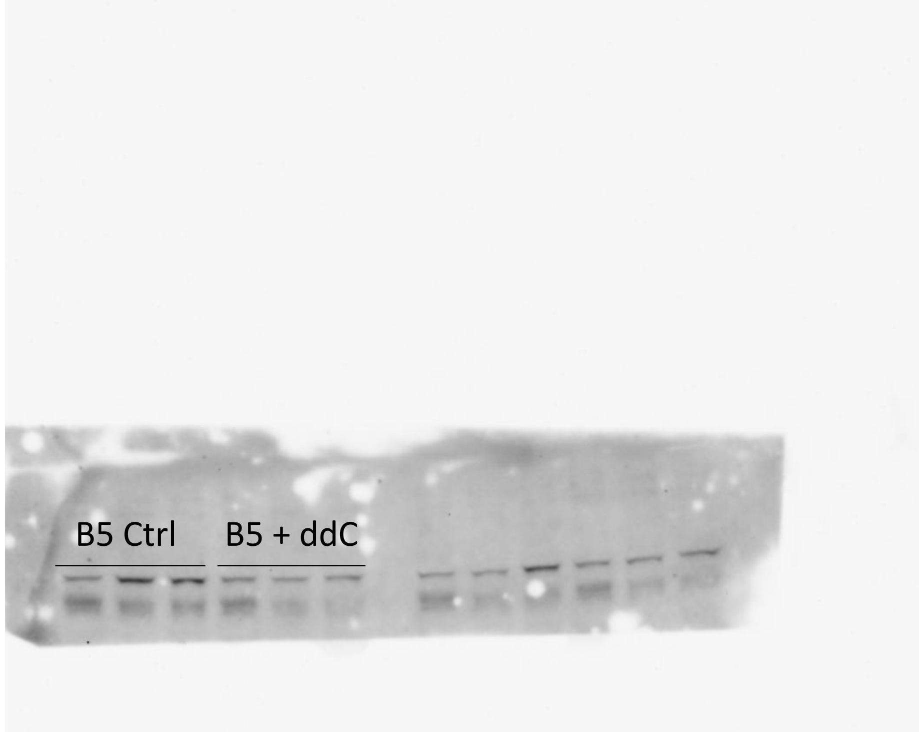

Gel order	Sample
1	**Marker**
2	Bclc5 control
3	Bclc5 Cabo 20uM 4h
4	Bclc5 Cabo 20uM 8h
5	Bclc5 control +ddc
6	Bclc5 +ddc Cabo 20uM 4h
7	Bclc5 +ddc Cabo 20uM 8h

WB Suplemental figures

- Figure 1.B PLC/PRF/5 Cells pTBK1

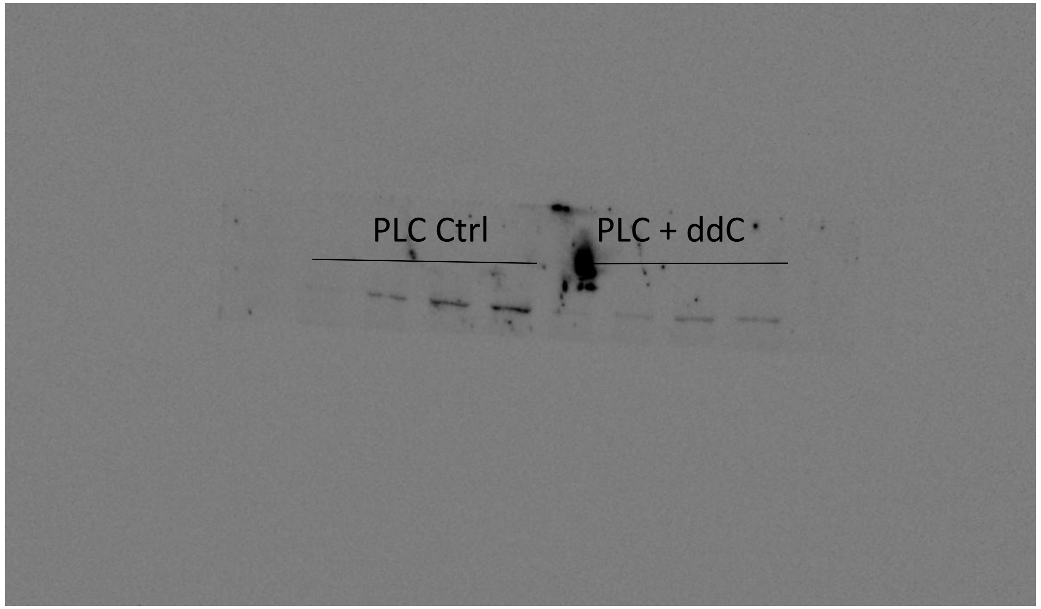

Gel order	Sample
1	**Marker**
2	PLC control
3	PLC Cabo 10uM 2h
4	PLC Cabo 10uM 4h
5	PLC Cabo 10uM 8h
7	PLC control +ddc
8	PLC +ddc Cabo15uM 2h
9	PLC +ddc Cabo15uM 4h
6	PLC +ddc Cabo15uM 8h

WB Suplemental figures

- Figure 1.B PLC/PRF/5 Cells TBK1

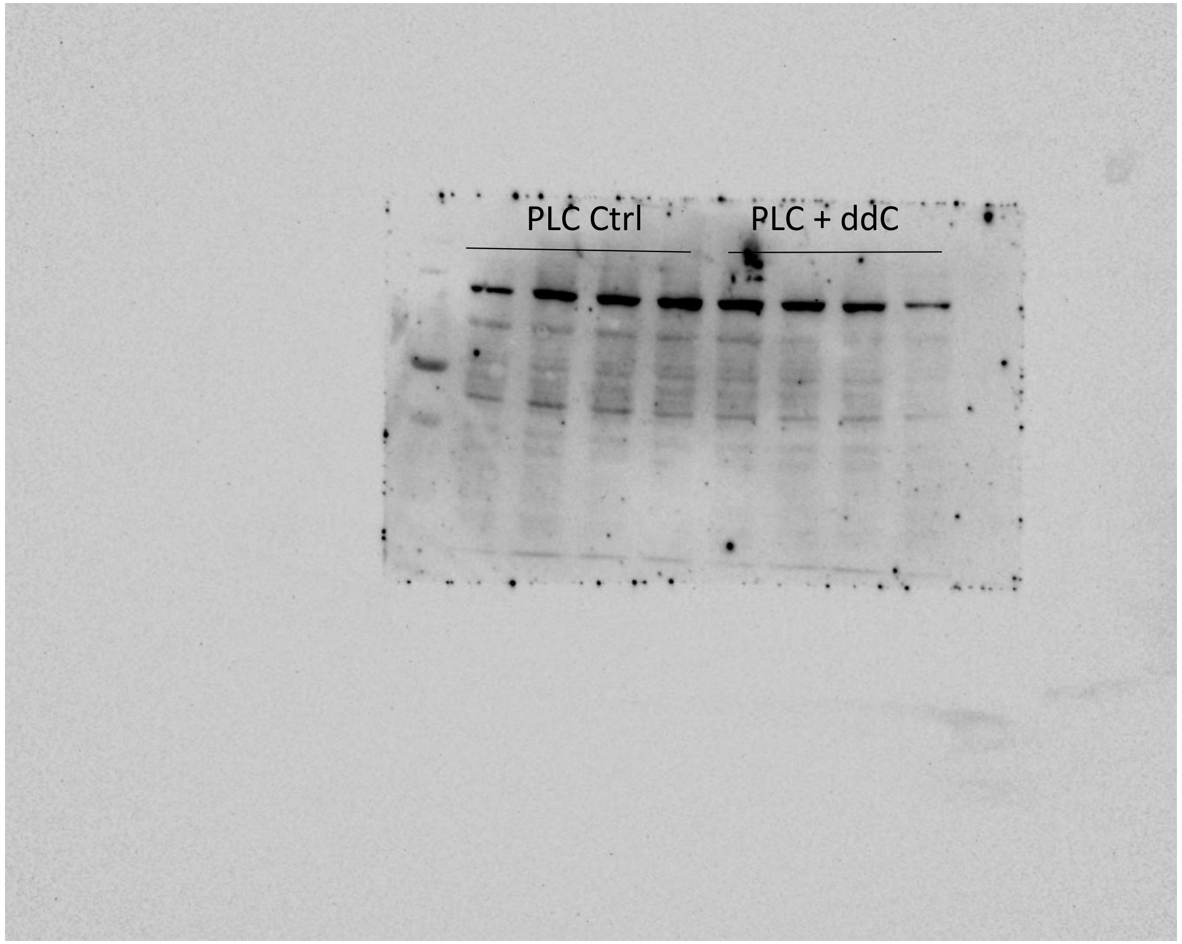

Gel order	Sample
1	**Marker**
2	PLC control
3	PLC Cabo 10uM 2h
4	PLC Cabo 10uM 4h
5	PLC Cabo 10uM 8h
7	PLC control +ddc
8	PLC +ddc Cabo15uM 2h
9	PLC +ddc Cabo15uM 4h
6	PLC +ddc Cabo15uM 8h

WB Suplemental figures

- Figure 2 (upper panel) Hep3B cells CRISPR – STING

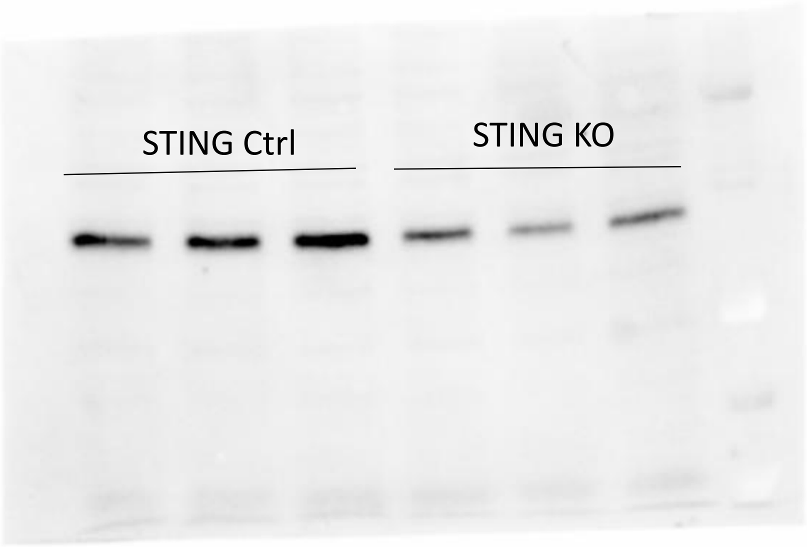

Gel order	Sample
1	Marker
2	1 Hep3B STING KO 1
3	5 Hep3B STING KO 2
4	3 Hep3B STING KO 3
5	7 Hep3B CNEG 1
6	11 Hep3B CNEG 2
7	9 Hep3B CNEG 3

WB Suplemental figures

- Figure 2 (upper panel) Hep3B cells CRISPR – STING

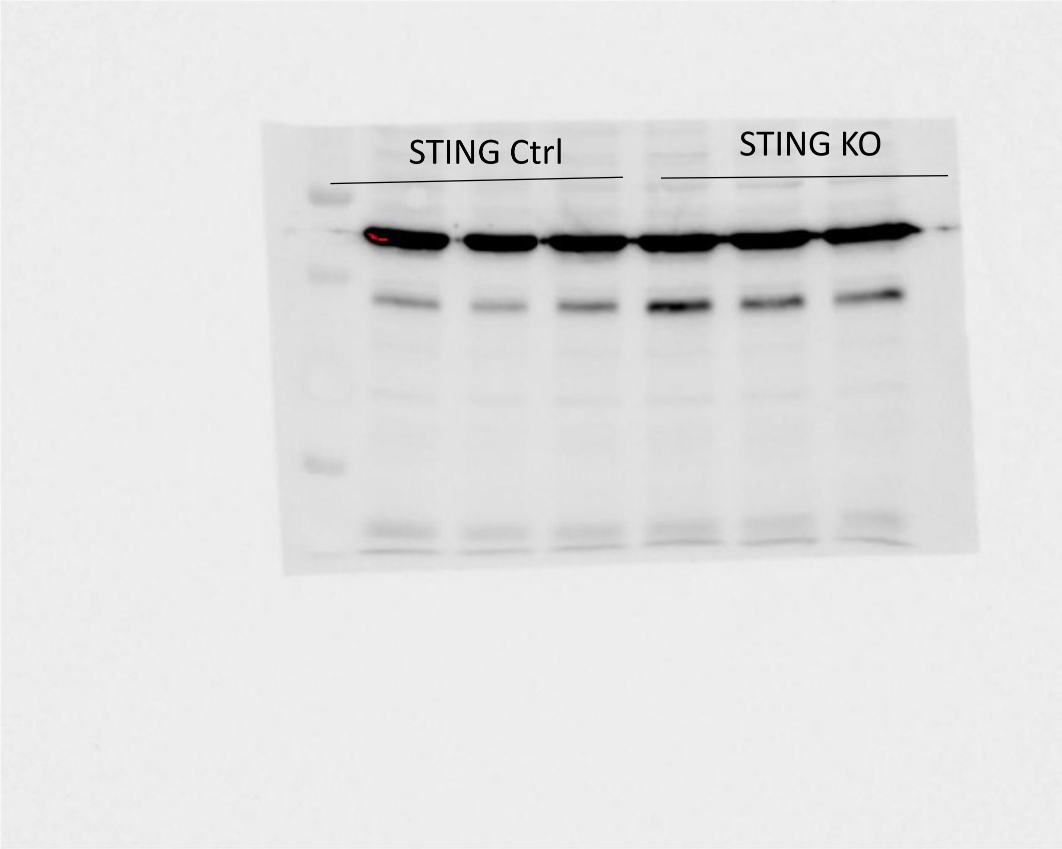

Gel order	Sample
1	Marker
2	1 Hep3B STING KO 1
3	5 Hep3B STING KO 2
4	3 Hep3B STING KO 3
5	7 Hep3B CNEG 1
6	11 Hep3B CNEG 2
7	9 Hep3B CNEG 3

WB Suplemental figures

- Figure 2 (lower panel) Hep3B cells CRISPR – TBK1

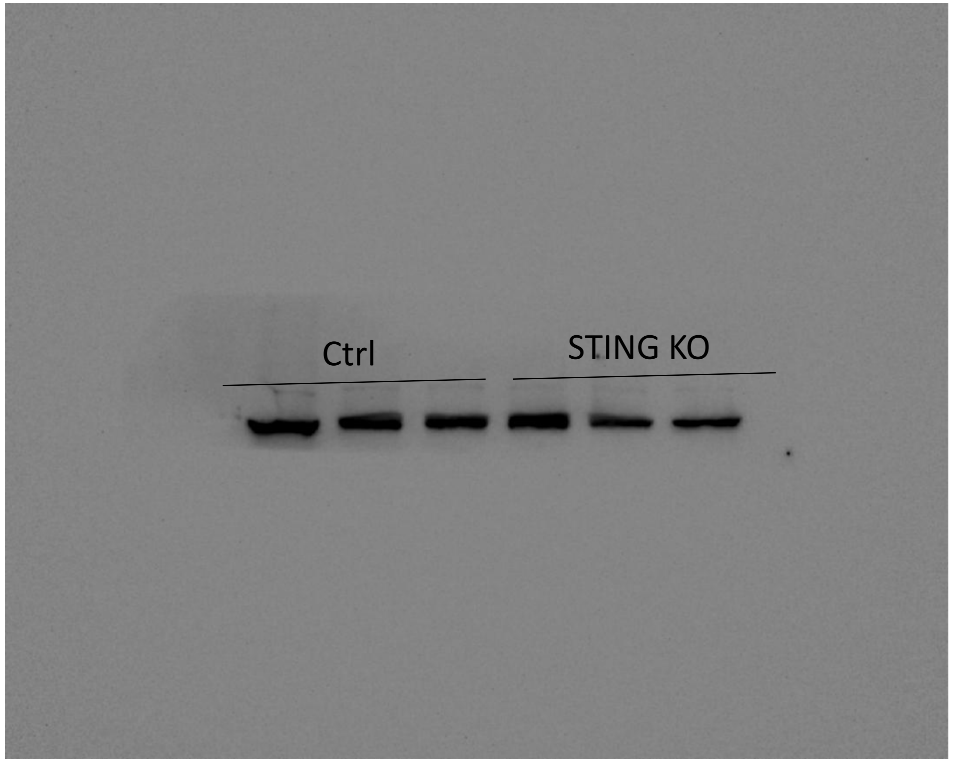

Gel order	Sample
1	Marker
2	1 Hep3B CNEG 1
3	2 Hep3B CNEG CABO 50uM 4H
4	3 Hep3B CNEG 3 CABO 50uM 4H
5	4 Hep3B STING KO 1
6	5 Hep3B STING KO 2 CABO 50uM 4H
7	6 Hep3B STING KO 3 CABO 50uM 4H

WB Suplemental figures

- Figure 2 (lower panel) Hep3B cells CRISPR – PTBK1

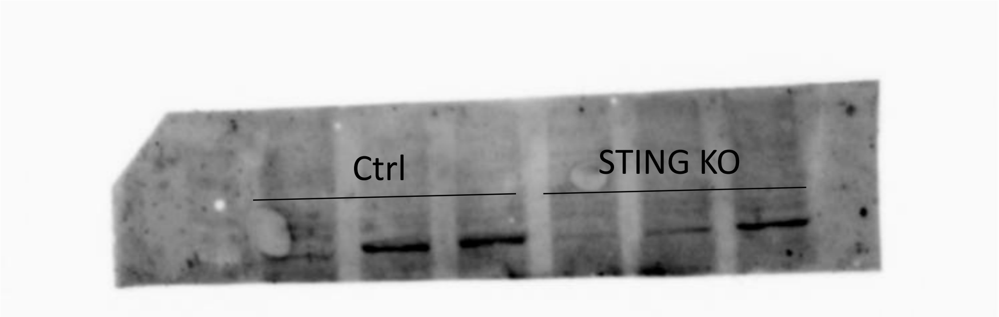

Gel order	Sample
1	**Marker**
2	**1 Hep3B CNEG 1**
3	**2 Hep3B CNEG CABO 50uM 4H**
4	**3 Hep3B CNEG 3 CABO 50uM 4H**
5	**4 Hep3B STING KO 1**
6	**5 Hep3B STING KO 2 CABO 50uM 4H**
7	**6 Hep3B STING KO 3 CABO 50uM 4H**

WB Supplemental figures

- Figure 4 RAW264.7 macrophages – pTBK1

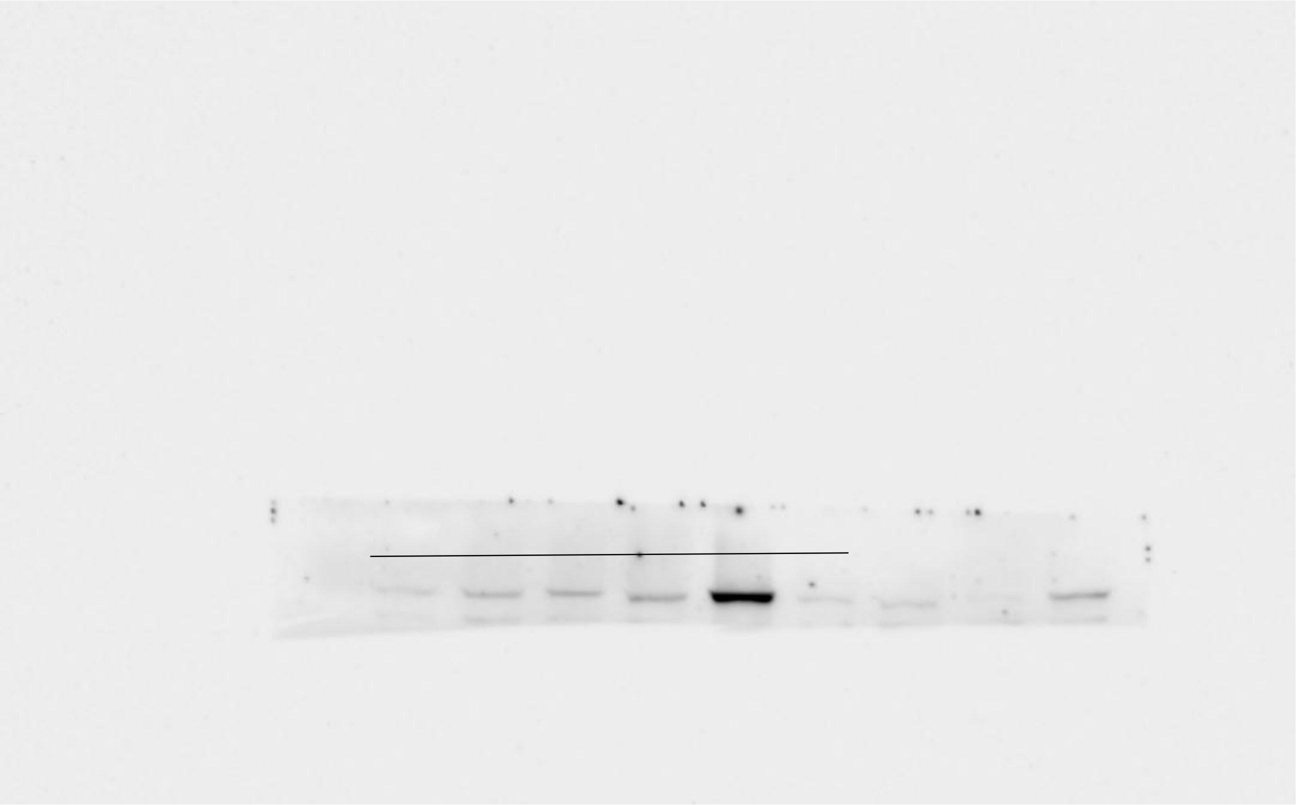

Gel Order	Sample
1	Marker
2	#1 RAW CONTROL
3	#1RAW CABO 50uM 2H
4	#1 RAW CABO 50uM 4H
5	#1 RAW CABO 50uM 8H
5	#1 RAW VAD 100uM 30'
6	#1 RAW CONTROL

WB Supplemental figures

- Figure 4 RAW264.7 macrophages – TBK1

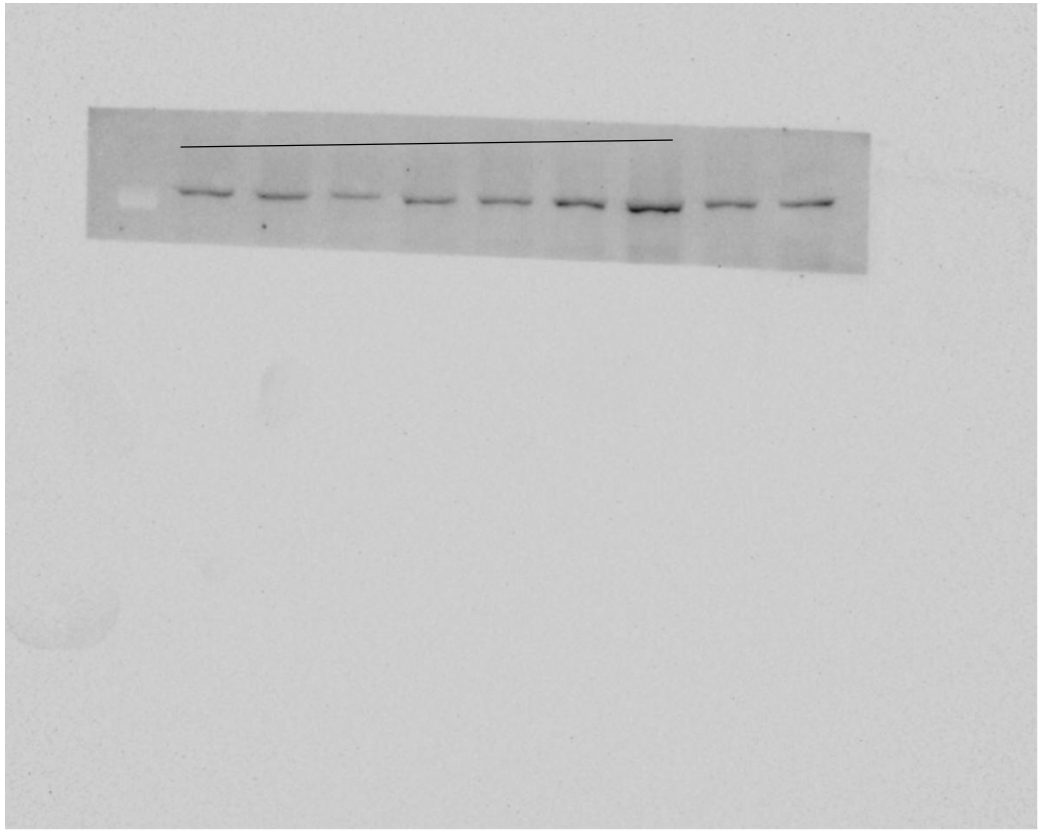

Gel Order	Sample
1	Marker
2	#1 RAW CONTROL
3	#1RAW CABO 50uM 2H
4	#1 RAW CABO 50uM 4H
5	#1 RAW CABO 50uM 8H
5	#1 RAW VAD 100uM 30'
6	#1 RAW CONTROL
7	#1RAW CABO 50uM 2H
8	#1 RAW CABO 50uM 4H
9	#1 RAW CABO 50uM 8H

WB Supplemental figures

- Figure 7- IN VIVO TUMORS pTBK1

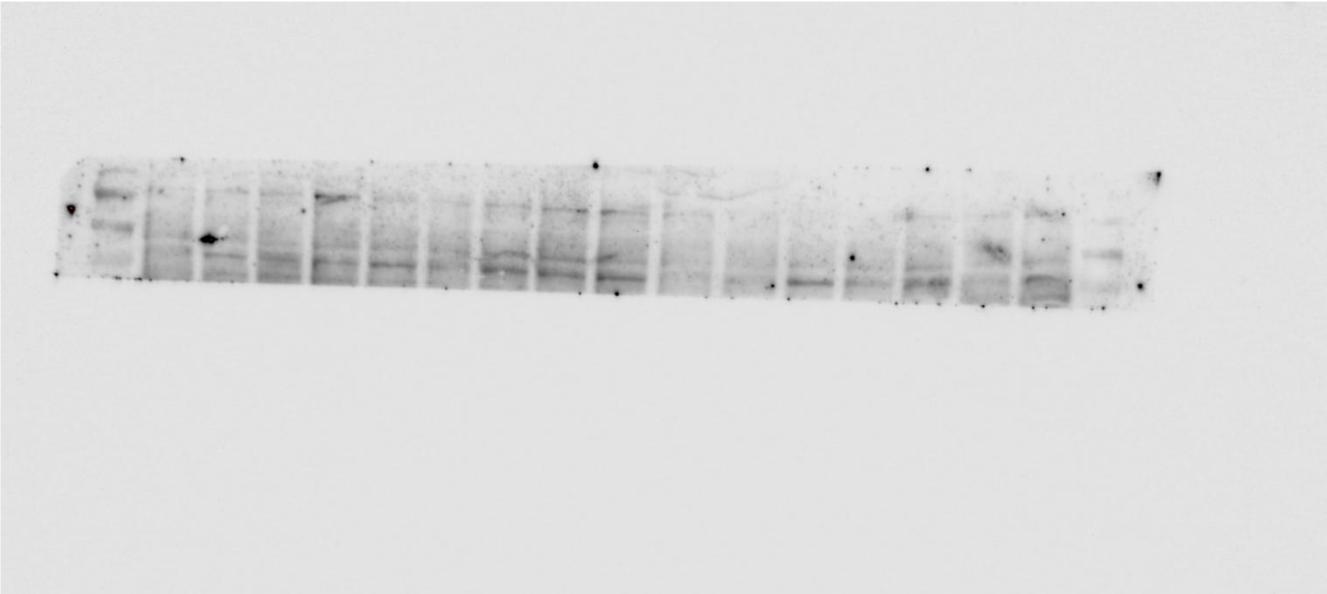

Gel order	Sample
1	**Marker**
2	T3D CTRL
3	T13E CTRL
4	T14E CTRL
5	T14D CTRL
6	T20E CABO
7	T20D CABO
8	T21E CABO
9	T21D CABO
10	T6D CABO+VAD
11	T8D CABO+VAD
12	T22E CABO+VAD
13	T22D CABO+VAD
14	T5E VAD
15	T4E VAD
16	T4D VAD
17	T12D VAD

WB Supplemental figures

- Figure 7- IN VIVO TUMORS TBK1

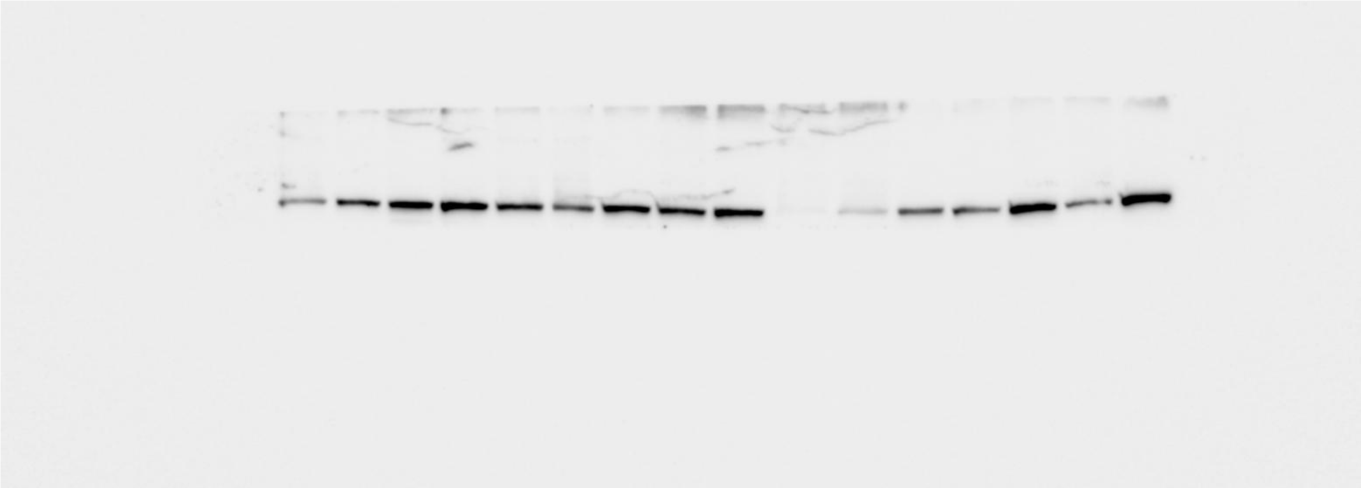

Gel order	Sample
1	**Marker**
2	T3D CTRL
3	T13E CTRL
4	T14E CTRL
5	T14D CTRL
6	T20E CABO
7	T20D CABO
8	T21E CABO
9	T21D CABO
10	T6D CABO+VAD
11	T8D CABO+VAD
12	T22E CABO+VAD
13	T22D CABO+VAD
14	T5E VAD
15	T4E VAD
16	T4D VAD
17	T12D VAD

WB Supplemental figures

- Figure 7- IN VIVO TUMORS ACTIN

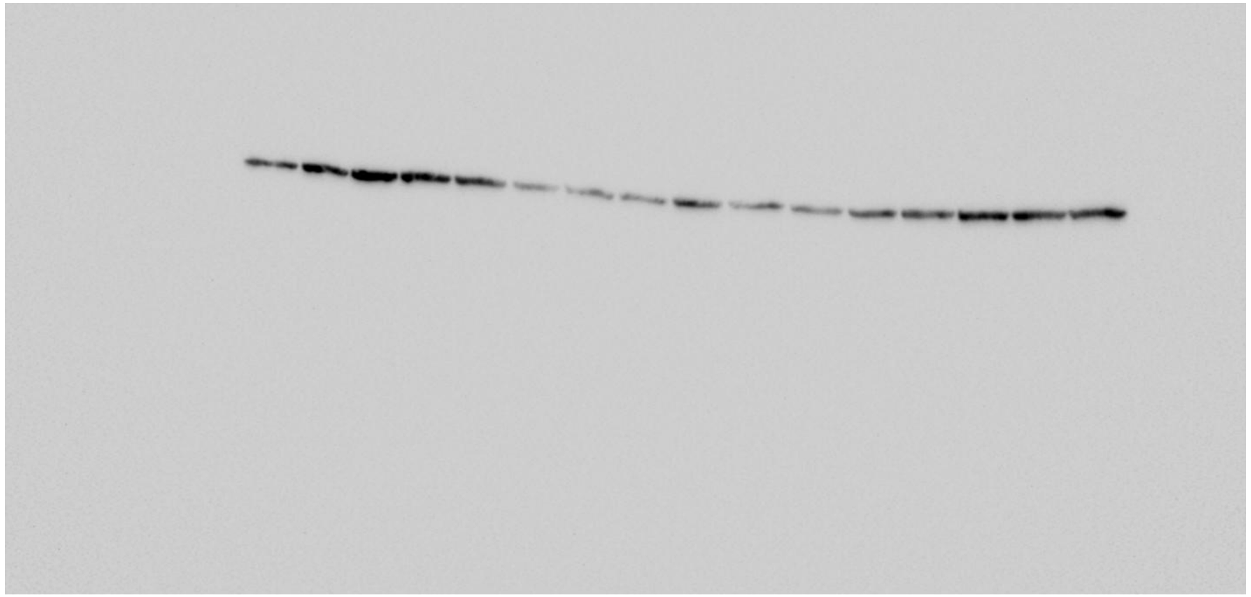

Gel order	Sample
1	**Marker**
2	T3D CTRL
3	T13E CTRL
4	T14E CTRL
5	T14D CTRL
6	T20E CABO
7	T20D CABO
8	T21E CABO
9	T21D CABO
10	T6D CABO+VAD
11	T8D CABO+VAD
12	T22E CABO+VAD
13	T22D CABO+VAD
14	T5E VAD
15	T4E VAD
16	T4D VAD
17	T12D VAD

WB Supplemental figures

- Figure 7- IN VIVO TUMORS pSTING

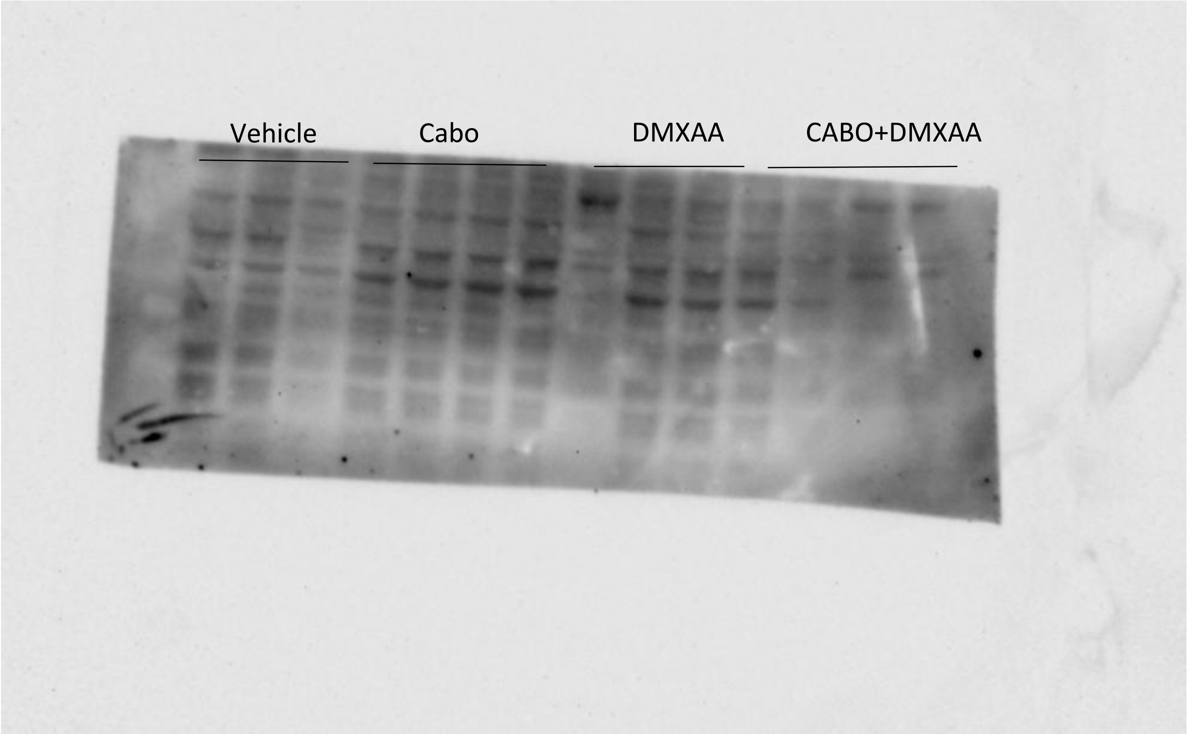

Gel order	Sample
1	**Marker**
2	V1E
3	V1D
4	V2D
5	C1E
6	C1D
7	C2E
8	C2D
9	D1D
10	D2E
11	D2D
12	CD3E
13	CD1D
14	CD2E
15	CD2D

WB Supplemental figures

- Figure 7- IN VIVO TUMORS STING

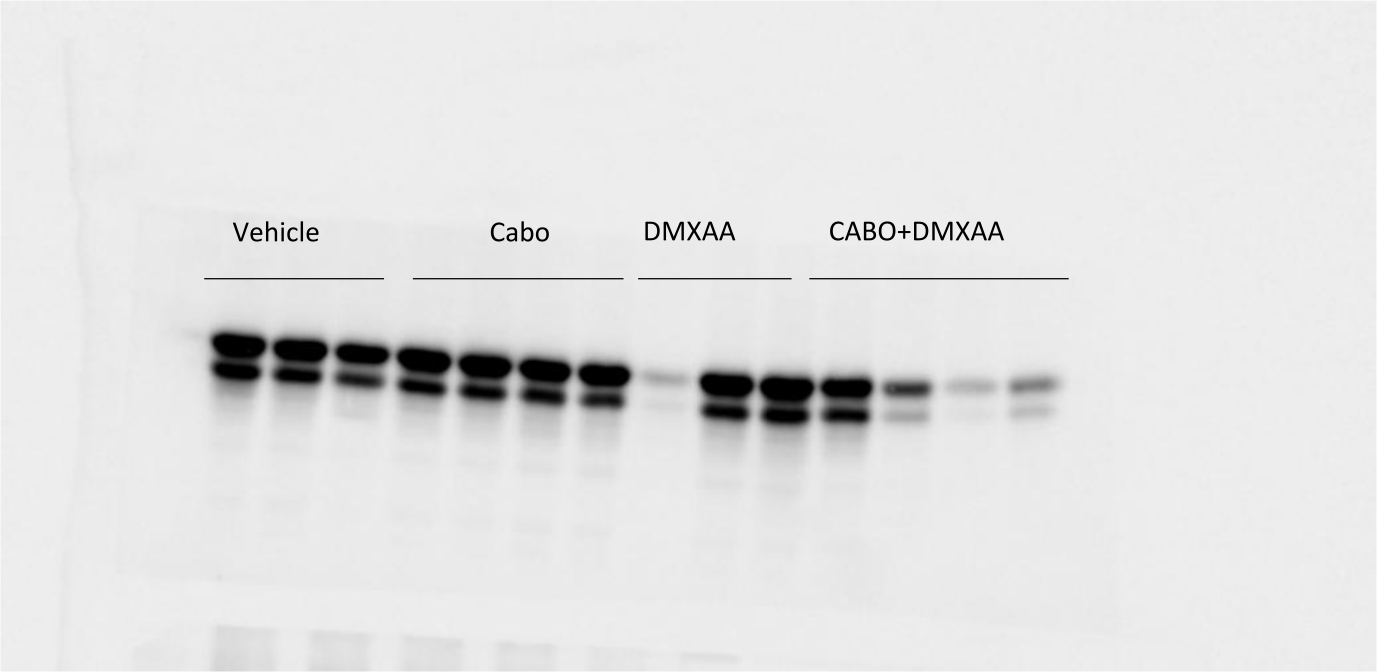

Gel order	Sample
1	**Marker**
2	V1E
3	V1D
4	V2D
5	C1E
6	C1D
7	C2E
8	C2D
9	D1D
10	D2E
11	D2D
12	CD3E
13	CD1D
14	CD2E
15	CD2D

WB Supplemental figures

- Figure 7- IN VIVO TUMORS ACTIN

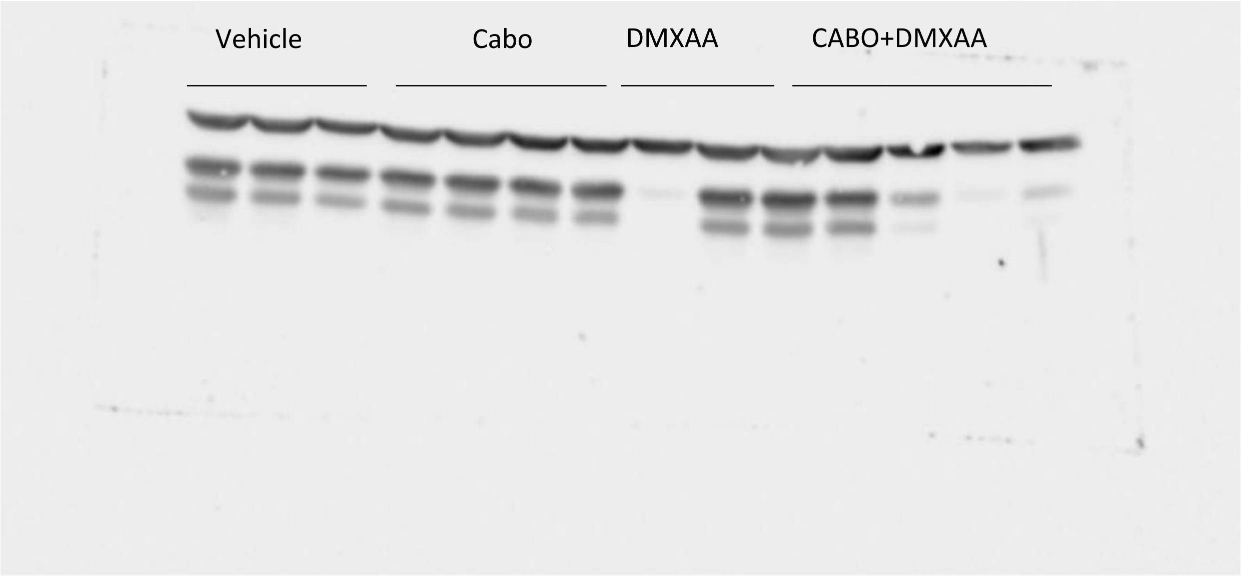

Gel order	Sample
1	**Marker**
2	V1E
3	V1D
4	V2D
5	C1E
6	C1D
7	C2E
8	C2D
9	D1D
10	D2E
11	D2D
12	CD3E
13	CD1D
14	CD2E
15	CD2D
